# Interleukin (IL)-2 Is a Key Regulator of T Helper 1 and T Helper 2 Cytokine Expression in Fish: Functional Characterization of Two Divergent *IL2* Paralogs in Salmonids

**DOI:** 10.3389/fimmu.2018.01683

**Published:** 2018-07-26

**Authors:** Tiehui Wang, Yehfang Hu, Eakapol Wangkahart, Fuguo Liu, Alex Wang, Eman Zahran, Kevin R. Maisey, Min Liu, Qiaoqing Xu, Mónica Imarai, Christopher J. Secombes

**Affiliations:** ^1^Scottish Fish Immunology Research Centre, School of Biological Sciences, University of Aberdeen, Aberdeen, United Kingdom; ^2^Division of Fisheries, Department of Agricultural Technology, Faculty of Technology, Mahasarakham University, Kantharawichai, Thailand; ^3^Department of Internal Medicine, Infectious and Fish Diseases, Faculty of Veterinary Medicine, Mansoura University, Mansoura, Egypt; ^4^Laboratorio de Immunologia, Centro de Biotecnología Acuícola, Departamento de Biología, Facultad de Química y Biología, Universidad de Santiago de Chile, Santiago, Chile; ^5^College of Animal Science and Technology, Northeast Agriculture University, Harbin, China; ^6^School of Animal Science, Yangtze University, Jingzhou, China

**Keywords:** salmonids, interleukin-2, expression, bioactivity, T cell growth factor, T helper 1, T helper 2, phagocytosis

## Abstract

Mammalian interleukin (IL)-2 is a cytokine centrally involved in the differentiation and survival of CD4+ T helper subsets and CD4+ T regulatory cells and in activation of cytotoxic effector lymphocytes. In bony fish, *IL2* orthologs have been identified with an additional divergent *IL2-Like* gene on the same locus present in several fish species. We report here two divergent *IL2* paralogs, *IL2A* and *IL2B*, in salmonids that originated from the whole genome duplication event in this fish lineage. The salmonid *IL2* paralogs differ not only in sequence but also in exon sizes. The IL-2 isoforms that are encoded have disparate pI values and may have evolved to preferentially bind specific IL-2 receptors. Rainbow trout *IL2* paralogs are highly expressed in thymus, spleen, gills, kidney and intestine, important tissues/organs in fish T cell development and function. Their expression in peripheral blood leukocytes (PBL) is low constitutively but can be upregulated by the mixed leukocyte reaction, by the T cell mitogen phytohemagglutinin and by signal mimics of T cell activation (phorbol 12-myristate 13-acetate and calcium ionophore). Both trout IL-2 isoforms promoted PBL proliferation and sustained high-level expression of *CD4* and *CD8*, suggesting that trout IL-2 isoforms are T cell growth/survival factors mainly expressed by activated T cells. The recombinant proteins for these two trout *IL2* paralogs have been produced in *E. coli* and possess shared but also distinct bioactivities. IL-2A, but not IL-2B, induced *IL12P35A1* and *CXCR1* expression in PBL. IL-2B had a stronger effect on upregulation of the T helper 1 (Th1) cytokine *interferon-γ* (*IFNγ*) and could sustain *CD8α* and *CD8β* expression levels. Nevertheless, both cytokines upregulated key Th1 (*IFNγ1, IFNγ2, TNFα2* and *IL12*) and T helper 2 (Th2) cytokines (*IL4/13B1* and *IL4/13B2*), cytokine and chemokine receptors and the antimicrobial peptide *cathelicidin-1* but had limited effects on T helper 17 cytokines and *TGFβ1* in PBL. They could also enhance PBL phagocytosis. These results suggest, for the first time in fish, that IL-2 isoforms may have an important role in regulating Th1 and Th2 cell development, and innate and adaptive host defenses in fish, and shed light on lineage-specific expansion, evolution, and functional diversification of *IL2* in vertebrates.

## Introduction

The T cell growth factor interleukin (IL)-2 was discovered in 1976 (1) and its cDNA cloned in 1983 in humans (2). It has a wide range of actions, including the ability to boost the cytolytic activity of natural killer (NK) cells and T cells, augment immunoglobulin production by activated B cells, maintain homeostatic proliferation of regulatory T cells (Treg cells), induce innate lymphoid cells (ILCs) and effector T cell differentiation, as well as influence memory T cells, effector T cells and monocytes (3–5). In T helper (Th) cell differentiation, IL-2 modulates the expression of receptors for other cytokines and transcription factors, thereby either promoting or inhibiting cytokine cascades that correlate with each Th cell development state (6). Thus, IL-2 promotes naïve T cell differentiation into T helper 1 (Th1) and T helper 2 (Th2) cells while inhibiting T helper 17 (Th17) cell development (5–7).

The production of IL-2 in mammals is tightly regulated and largely restricted to activated CD4+ T cells (6). Other populations of cells, including activated CD8+ T cells, NK cells and NKT cells, dendritic cells and mast cells have also been reported to secrete IL-2, albeit at much lower levels than activated CD4+ T cells (8, 9). At resting conditions, CD4 Th cells are the main source of the constant but low levels of IL-2. On immune activation of the T cell receptor (TCR) by antigens presented by antigen-presenting cells (APCs) and costimulatory signals, T cells (CD4+ and CD8+ T cells) start to secrete large amounts of IL-2 (3, 4). *In vitro*, IL-2 can be induced in T cells by T cell mitogens and co-mitogens, such as phytohemagglutinin (PHA), phorbol 12-myristate 13-acetate (PMA), and calcium ionophore (CI) (10).

Structurally, IL-2 is a short-chain type I cytokine with a four α-helical bundle (helices A–D) “up-up-down-down” configuration typical of this family of cytokines. In this configuration, helices are aligned in a fashion that allows interaction with its receptor chains (11, 12). There are three IL-2 receptor components, IL-2Rα (CD25), IL-2Rβ (CD122) and IL-2Rγ (CD132, also known as γC), forming three classes of IL-2 receptors. The low-affinity receptor contains only IL-2Rα, the intermediate-affinity receptor contains IL-2Rβ and γC, and the high-affinity receptor contains all three chains (3, 4). IL-2 activates three main signaling pathways, including the JAK–STAT pathway, the RAS–MAP kinase pathway, and the PI3-kinase/AKT pathway. Kinetically, IL-2 first interacts with IL-2Rα, resulting in a conformational change in IL-2 that then allows it to efficiently interact with IL-2Rβ, with γC subsequently recruited (3, 4, 11, 12).

The γC receptor chain is shared by receptors for IL-2, IL-4, IL-7, IL-9, IL-15, and IL-21 ligands that form the γC cytokine family (or IL-2 family). In mammals, each cytokine in this family has a unique private α receptor chain in addition to γC. Furthermore, IL-2 and IL-15 share IL-2Rβ as well as γC. The receptor chains of known fish γC cytokines are present in fish (13) with the apparent exception of the IL-2Rα. It has been suggested that in fish IL-2 and IL-15 may both bind to IL-15Rα (also termed CD25-like, CD25L) (14). A second *IL15*-related gene (*IL15L*) identified in fish was later found to be present in other vertebrate groups but is a pseudogene in man and mouse (15). The cow IL-15L binds to the IL-15Rα but not the IL-2Rα, as seen with IL-15 itself (15). It is noteworthy that mammalian IL-2Rα and IL-15Rα are distinctive cytokine receptor proteins that contain sushi domains at the N-terminal but lack domains typical of class I cytokine receptor proteins (13). The genes encoding these two proteins have a similar intron–exon organization and are very closely linked on human chromosome (CH)10 and mouse CH2, suggesting duplication of an ancient precursor of both genes (16). The lack of fish IL-2Rα may suggest that fish IL-2, IL-15, and IL-15L may share three receptor chains, the CD25L, IL-2Rβ, and γC.

Mammalian IL-2 functionally signals *via* either high or intermediate-affinity IL-2 receptors. In mammals, the three receptor chains are located on different CHs and differentially expressed and modulated (17). Many immune cells can respond to IL-2, but their sensitivity to IL-2 varies based on the types of IL-2 receptors expressed and the induction vs constitutive expression of the different IL-2 receptor chains. *IL2Rα* is constitutively expressed on Treg cells and ILC2 cells, whereas it is more transiently induced on activated lymphocytes (7). Although *IL2Rβ* is constitutively expressed, it is also induced in T cells by activation *via* TCR and IL-2 stimulation, albeit to a lesser extent than *IL2Rα*. *γC* is constitutively expressed in the lympho-hematopoietic lineage (17). Thus, on resting lymphocytes and NK cells, IL-2 signals *via* intermediate-affinity IL-2 receptors, whereas activated lymphocytes, Treg, and ILC2 cells additionally express IL-2Rα and therefore have both high- and low-affinity receptors. Interestingly, activated dendritic cells have been reported to express IL-2Rα and to be capable of binding secreted IL-2 and trans-presenting it to neighboring cells expressing IL-2Rβ and γC (18). Following receptor binding, IL-2 activates multiple signaling pathways to activate the expression of genes essential for effector cell function, differentiation, and T cell growth (7).

Two *IL2*-related genes, *IL2* and *IL2L*, have been described in fish (19). *IL2* was originally discovered by analysis of the fugu *Takifugu rubripes* genome sequence that also identified *IL21* as a neighboring gene as in mammals (20). An *IL2L* gene has also been discovered in several percomorph fish genomes (e.g., fugu, tetraodon *Tetraodon nigroviridis*, and stickleback *Gasterosteus aculeatus*) next to *IL2*. Both fish genes had a 4 exon/3 intron organization, as seen in mammals, but only shared 22–25% amino acid (aa) identity in the same species. *IL2* has since been cloned in rainbow trout *Oncorhynchus mykiss* (21, 22), and other species, but the bioactivity of fish IL-2 has only been reported in rainbow trout (23). The trout IL-2 recombinant protein induces expression of *interferon-γ* (*IFNγ*), the CXC chemokine *γIP, IL4/13B*, and to a lesser extent *IL2, STAT5* and *Blimp-1*, and *IL17A/F* genes in head kidney (HK) cells (21, 24, 25).

In this report, a second *IL2*-related gene, *IL2B* (with the previous one now termed *IL2A*), has been cloned in Atlantic salmon *Salmo salar*, and rainbow trout. These two *IL2* genes have also been identified in other available salmonid genomes, including coho salmon *Oncorhynchus kisutch*, chinook salmon *Oncorhynchus tshawytscha* and Arctic char *Salvelinus alpinus*, and arose *via* the salmonid whole genome duplication [WGD; (26)] event. IL-2A and IL-2B share only 39–43% aa sequence identity, suggesting that they may have changed functionally. The expression of both genes in rainbow trout was activated by the mixed leukocyte reaction (MLR), by the T cell mitogen PHA, and was synergistically induced by PMA and CI in peripheral blood leukocytes (PBL). Recombinant proteins for trout *IL2A* and *IL2B* have been produced in *E. coli* and tested functionally in PBL. Both cytokines upregulated the expression of genes involved in Th1 and Th2 pathways, sustained high-level expression of T cell markers but had limited ability to modulate the pro-inflammatory (Th17) and Treg cell pathways. They also promoted the proliferation of PBL *in vitro* and enhanced phagocytosis. This study suggests that fish IL-2 molecules are important T cell cytokines that regulate the Th1 and Th2 pathways and antimicrobial defense in fish.

## Materials and Methods

### Fish

Juvenile rainbow trout were purchased from College Mill Trout Farm (Perthshire, UK) and maintained in aerated fiberglass tanks supplied with a continuous flow of recirculating freshwater at 14°C. Fish were fed twice daily on a commercial pellet diet (EWOS) and were given at least 2 weeks of acclimatization prior to treatment. All the experiments described comply with the Guidelines of the European Union Council (2010/63/EU) for the use of laboratory animals and were carried out under UK Home Office project license PPL 60/4013, approved by the ethics committee at the University of Aberdeen.

### Cloning of Salmonid *IL2B* cDNA in Atlantic Salmon and Rainbow Trout

The *IL2B* cloning was performed in 2011 when no trout whole genome shotgun sequences (WGS) were available. BLAST (27) search using know salmonid and other fish *IL2* at the National Center for Biotechnology Information (NCBI) identified a salmon WGS contig (acc. no. AGKD04000795) that could encode for a second *IL2* (termed *IL2B* thereafter). Exons were predicted and primers were designed to the 5′-untranslated region (UTR) (sIL2BF1–2, Table S1 in Supplementary Material) and used for 3′-RACE (rapid amplification of cDNA ends) using salmon SMART cDNA prepared from PMA and CI stimulated blood samples, as described previously (28). A 0.9 kb 3′-RACE product was obtained, cloned, sequenced, and encoded the salmon IL-2B (acc. no. HE805272). A cDNA fragment of 0.75 kb was also amplified from trout cDNA using salmon primers sIL2BF1 and R1 (Table S1 in Supplementary Material) and encoded trout IL-2B (acc. no. HE805273).

### Sequence Analysis

The nucleotide sequences generated were assembled and analyzed with the AlignIR program (LI-COR, Inc.). Homology search was performed at NCBI using the BLAST program^1^ (27). The gene organization was predicted using the Spidey program at NCBI. Protein prediction was performed using software at the ExPASy Molecular Biology Server^2^ (29) and signal peptides were predicted using the SignalP4.0 program (30). Disulfide bonding and cysteine connectivity were predicted using the DISULFIND program^3^ (31). Protein secondary structure was predicted using Jpred4 program^4^ (32). Global sequence comparison was performed using the scoring matrix BLOSUM62 within the MatGAT program, with a gap open penalty of 10 and gap extension penalty of 1 (33). Multiple sequence alignments were generated using CLUSTALW (34). The synteny of IL-2 loci was analyzed using Genomicus (database version 75.01) (35). A neighbor-joining phylogenetic tree was constructed on full-length aa multiple alignments using the MEGA7.0 software (36). The evolutionary distances were computed using the JTT matrix-based method with all ambiguous positions removed for each sequence pair.

### Tissue Distribution of Rainbow Trout *IL2* Paralogs

Six healthy rainbow trout (~140 g) were killed and 17 tissues (blood, thymus, gills, scales, skin, muscle, tail fins, adipose fin, brain, adipose tissue, spleen, liver, heart, intestine, gonad, HK, and caudal kidney) were collected and processed as described previously (24, 25). The RNA preparation, cDNA synthesis, and real-time PCR analysis of gene expression were also as described previously (37). The primers for real-time PCR were designed, so that at least one primer crossed an intron to ensure that genomic DNA could not be amplified under the PCR conditions used. The primers for qPCR expression analysis of *IL2* paralogs are detailed in Table S1 in Supplementary Material and for other genes in Table S3 in Supplementary Material. To directly compare the expression level of the different *IL2* paralogs, a reference was constructed using equal molar amounts of PCR product from each gene, including the house keeping gene *elongation factor-1α* (*EF1α*). The relative expression level of each sample was normalized against the expression level of *EF1α*.

### PBL Preparation

The PBL were prepared by hypotonic disruption of erythrocytes, using a method modified from the study by Crippen et al. (38). The detailed protocol and characterization of the PBL has been reported elsewhere (39). Briefly, blood was withdrawn from the caudal vein of rainbow trout (200–500 g/fish) using a BD Vacutainer Plus blood collection tube (with Lithium heparin, BD, UK). The red blood cells were lysed by combining blood and ice-cold cell culture grade water at a ratio of 1:9 and mixed for 20 s. The osmotic pressure was brought back by adding 10× PBS (Sigma, UK). The resultant PBL preparation was kept on ice for 5–10 min to allow the cell debris to settle. The PBL were then separated from cell debris by passing through an EASYstrainer (70 µm, Greiner Bio-One, UK), pelleted by centrifugation (200 *g*, 5 min), and washed once with incomplete cell culture medium [Leibovitz medium L-15 (Life Technologies) supplemented with 100 IU/ml penicillin, 100 µg/ml streptomycin, and 1% fetal calf serum (FCS, Sigma, UK)]. The resultant PBLs were resuspended in complete cell culture medium (as above except 10% FCS) at 2 × 10^6^ PBL/ml, ready for culture or stimulation.

### Modulation of the Expression of Trout *IL2* Paralogs in PBL

Freshly prepared PBL isolated as described above were stimulated with the T cell mitogen PHA (at 10 µg/ml), for 4, 8, 24 and 48 h. The PBL were also stimulated with PMA (50 ng/ml) or CI A23187 (100 ng/ml) alone or together for the same time points. The stimulation was terminated by dissolving the cells in TRI reagent (Sigma, UK). Quantification of gene expression was as described above. *IL2* expression was expressed as arbitrary units where the expression level in control cells at 4 h equals 1.

### Modulation of the Expression of Trout *IL2* Paralogs in PBL and Primary HK Cells During the MLR

A MLR from three individual fish was set up to increase the magnitude and decrease the variation of the *in vitro* response (40). PBL prepared as above or HK cells isolated as described previously (24, 25, 41) were resuspended at 2 × 10^6^ leukocytes/ml and mixed following a loop design where an equal number of cells from 6 fish (1–6) were mixed as follows: 1/2/3, 2/3/4, 3/4/5, 4/5/6, 5/6/1, and 6/1/2. The cells from individual fish at the same density were used as control. The cells were seeded in 12-well cell culture plates at 2 ml/well or in 6-well plates at 3 ml/well. The plates were then sealed (Thermo Fisher Scientific, UK) and incubated at 20°C. The cell cultures were terminated at 4–120 h by dissolving in TRI reagent and *IL2* gene expression was quantified as above. A fold change was calculated as the average expression level of mixed cells to that of cells from individual fish at the same time point.

### Cloning, Expression, and Purification of Recombinant Trout IL-2 Isoforms

#### Cloning

The sequences encoding the two trout IL-2 mature peptides were amplified from cloned cDNA using the primers detailed in Table S1 in Supplementary Material. The amplified products were cloned to a pET vector (Novagen) as described previously (24, 37, 42–44). Each construct has a His-tag (MGSHHHHHHHHS) at the N-terminus for translation initiation and purification. Thus, the recombinant trout IL-2A and IL-2B were 134 aa and 129 aa, with a calculated molecular weight/theoretical pI of 15.1 kDa/5.96 and 14.5 kDa/7.80, respectively.

#### Expression and Purification of Trout IL-2 Isoforms

For each protein, a sequence confirmed plasmid was transformed into BL21 Star (DE3) competent cells (Invitrogen). The induction of recombinant protein production, purification under denaturing conditions, refolding, re-purification under native conditions, SDS-PAGE analysis of proteins, and quantification of protein concentration were as described previously (24, 37, 42). The refolding buffer contained 50 mM Tris–HCl, pH 7.0 (for IL-2B) or pH 8.0 (for IL-2A), 10% glycerol, 0.5 M arginine monohydrochloride, 0.5% Triton X-100, 0.2% PEG3350, and 10 mM 2-mercaptoethanol (2-ME). The purified proteins were desalted in desalting buffer [50 mM Tris–HCl, pH 7.0 (for IL-2B) or pH 8.0 (for IL-2A), 140 mM NaCl, 10 mM arginine, 50% glycerol, and 5 mM 2-ME] using PD-10 Desalting Columns (GE Healthcare). After sterilization with a 0.2-µm filter, the recombinant proteins were aliquoted and stored at −80°C ready for stimulation of cells.

### Modulation of Gene Expression in PBL by Recombinant Trout IL-2 Isoforms

The recombinant proteins produced above were initially added to HK cells (2 × 10^6^ cells/ml) at 0.2–500 ng/ml for 24 h and the expression of *IFNγ* and *TNFα* analyzed. Further analysis of their bioactivity was focused in PBL using a dose of 200 ng/ml that showed good responses in HK cells. Freshly prepared PBL were stimulated with IL-2A, IL-2B, or both at 200 ng/ml for 4, 8, 24 and 48 h. The stimulation was terminated by dissolving the cells in TRI reagent, and real-time PCR analysis was conducted as described above. The expression of 75 trout immune genes, including those encoding for cellular markers, antimicrobial peptides, cytokines, and cytokine receptors, was analyzed. The primer information is detailed in Table S3 in Supplementary Material. The expression of each gene was first normalized to that of *EF1α*, and expressed as arbitrary units where one unit equals the average expression level in the control samples at 4 h. To give an estimation of constitutive expression in PBL, Δcp, the average cp (crossing point at which the fluorescence crosses the threshold during qPCR, *N* = 4) of a target gene minus that of the house keeping gene *EF1α* in control cells at 4 h is provided in Table S3 in Supplementary Material. A higher Δcp value indicates a lower expression level.

### Proliferation Assay

Peripheral blood leukocyte proliferation was quantified by measuring BrdU incorporation during DNA synthesis in replicating cells using a Cell Proliferation ELISA, BrdU (colorimetric) kit (Sigma, UK) as per the manufacturer’s instructions. Briefly, PBL from each fish in complete cell culture medium, at 4 × 10^5^ cells/well, were cultured in 96-well cell culture plates in the presence of 200 ng/ml of IL-2A or IL-2B. A control without IL-2 and a blank control without cells were also included. Three replicate wells were used for each treatment. The plates were then sealed and incubated at 20°C for 3 days. BrdU at 10 µM was added 20 h before fixation. The cell culture medium was removed after centrifugation (400 *g*, 5 min) and the cells fixed and DNA denatured by adding FixDenat solution. Anti-BrdU-peroxidase was then added to bind to BrdU incorporated in newly synthesized cellular DNA and detected using Tetramethylbenzidine. The color reaction was read at 450 nm using an ELISA plate reader (SoftMax Pr0 5.3). To calculate a stimulation index, the average OD450 of triplicates from each fish was first subtracted from the background value (without cell blank control). A stimulation index was calculated as the resulting OD450 of IL-2-stimulated cells divided by that of untreated samples.

### Phagocytic Assay

Peripheral blood leukocytes in complete cell culture medium prepared above (2 × 10^6^ cells/ml) were added to 12-well suspension cell culture plates (Greiner Bio-One) and incubated at 20°C. The fresh PBL were stimulated with recombinant IL-2A, IL-2B, or medium alone as control. Fluorescent latex beads (FluoSpheres Fluorescent Microspheres yellow green fluorescent, 1.0 μm, Life Technology) were added 24 h later at a cell/bead ratio of 1:20, and incubated for a further 3 h. The cells were harvested using 0.5% trypsin–EDTA (GIBCO) and the supernatant removed by centrifuging at 400 *g* for 3 min. Non-ingested beads were removed by centrifuging (100 *g* for 10 min at 4°C) over a 3% BSA and 4.5% d-glucose cushion prepared with FACS buffer (HBSS supplemented with 2% FCS, 5 mM EDTA, and 0.1% sodium azide). Cells were washed with FACS buffer and analyzed with a C6 Accuri Flow Cytometer, measuring at least 75,000 cells after live cell gating according to the FCS/SSC.

### Statistical Analysis

The data were analyzed statistically using the SPSS Statistics package 24 (SPSS Inc., Chicago, IL, USA). The real-time PCR data were scaled and log2 transformed before statistical analysis as described previously (37). One-way analysis of variance and the LSD *post hoc* test were used to analyze expression data in MLR, with *p* ≤ 0.05 between mixed and control groups considered significant. For the tissue distribution of expression and other *in vitro* experiments that consisted of sample sets from individual fish, a paired samples *T*-test was applied.

## Results

### Two Divergent *IL2* Paralog Arose From the Salmonid-Specific 4R WGD

#### Cloning and Sequence Analysis of Salmonid *IL2*

In addition to the known salmonid *IL2A* in rainbow trout (trout), a second *IL2* (*IL2B*) has been cloned in both trout and Atlantic salmon (Atlantic) (Figures S1 and S2 in Supplementary Material). Analysis of Atlantic salmon WGS contig (acc. no. AGKD04000200) resulted in the prediction of an Atlantic salmon *IL2A* with a translation that differs by two aa from the prediction in the database (acc. no. EU816603, Figure S3 in Supplementary Material). Further analysis of the recently released WGS of coho salmon (coho), chinook salmon (chinook), and Arctic char (char) resulted in the prediction of both *IL2A* and *IL2B* in all the species (Figures S4–S9 in Supplementary Material). The salmonid *IL2* sequences are summarized in Table S2 in Supplementary Material.

#### Gene Organization of Salmonid *IL2*

**Figure 1:**
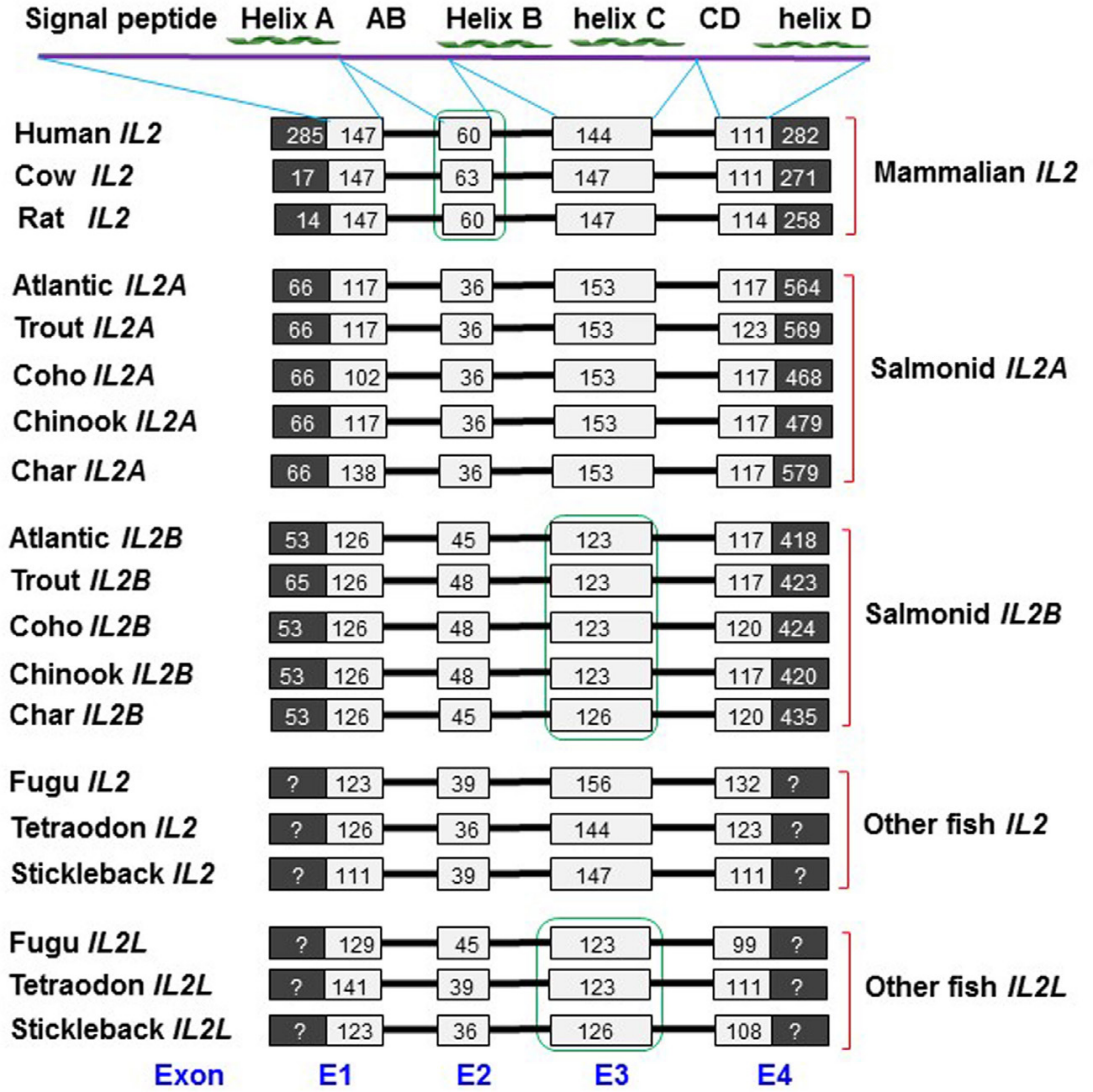
Comparison of the gene organization of salmonid *IL2* genes with mammalian *IL2* and other fish *IL2* and *IL2L* genes. The black and white boxes represent non-coding and amino acid (aa) coding regions, respectively. The black bars represent introns. The sizes (bp) of the coding regions are numbered in the boxes. The gene organization of the salmonid *IL2* genes was predicted using the Spidey program based on the sequence information from Table S2 and Figures S1–S9 in Supplementary Material. The human, cow, and rat *IL2* gene organization was extracted from Ensemble genes ENSG00000109471, ENSBTAG00000020883, and ENSRNOG00000017348, respectively. Other fish *IL2/IL2L* genes were extracted from the NCBI genomic sequences NC_018903 (Fugu), CAAE01023259 (Tetraodon), and AANH01006550 (Stickleback). The aa sequence domains (signal peptide, helices A–D, AB, and CD loops) encoded by each exon are indicated above.

Both the *IL2A* and *IL2B* genes, identified in the five salmonids, have a four-exon gene organization with three phase 0 introns (Figure 1; Figures S1–S9 in Supplementary Material). A four-exon organization is typical of mammalian *IL2* genes, and *IL2* and *IL2L* genes from other fish species (Figure 1). The mammalian IL-2 protein has a four helix-bundle structure (helices A–D), and this structure is predicted for each of the salmonid IL-2 molecules using the Jpred 4 program (32). Exon 1 encodes for the signal peptide and helix A, exon 2 for a large AB loop, exon 3 for helices B and C, and exon 4 encodes for a large CD loop and helix D. Lineage-specific and paralog-specific exon size differences are apparent. Mammalian *IL2* genes have a large coding region in exon 1 and a large exon 2, compared with fish *IL2* orthologs. Salmonid *IL2A* genes have a relatively smaller exon 2 (36 vs 45–48 bp) but larger exon 3 (153 vs 123–126 bp) relative to salmonid *IL2B*. The percomorph *IL2* genes also have a larger exon 3 (144–156 vs 123–123 bp) but in *IL2L* genes it is smaller (Figure 1). In general, salmonid *IL2A* has more similarity to *IL2* from other fish species, while salmonid *IL2B* is similar to *IL2L*.

#### Nucleic Acid and aa Sequence Analysis of Salmonid *IL2*

**Figure 2:**
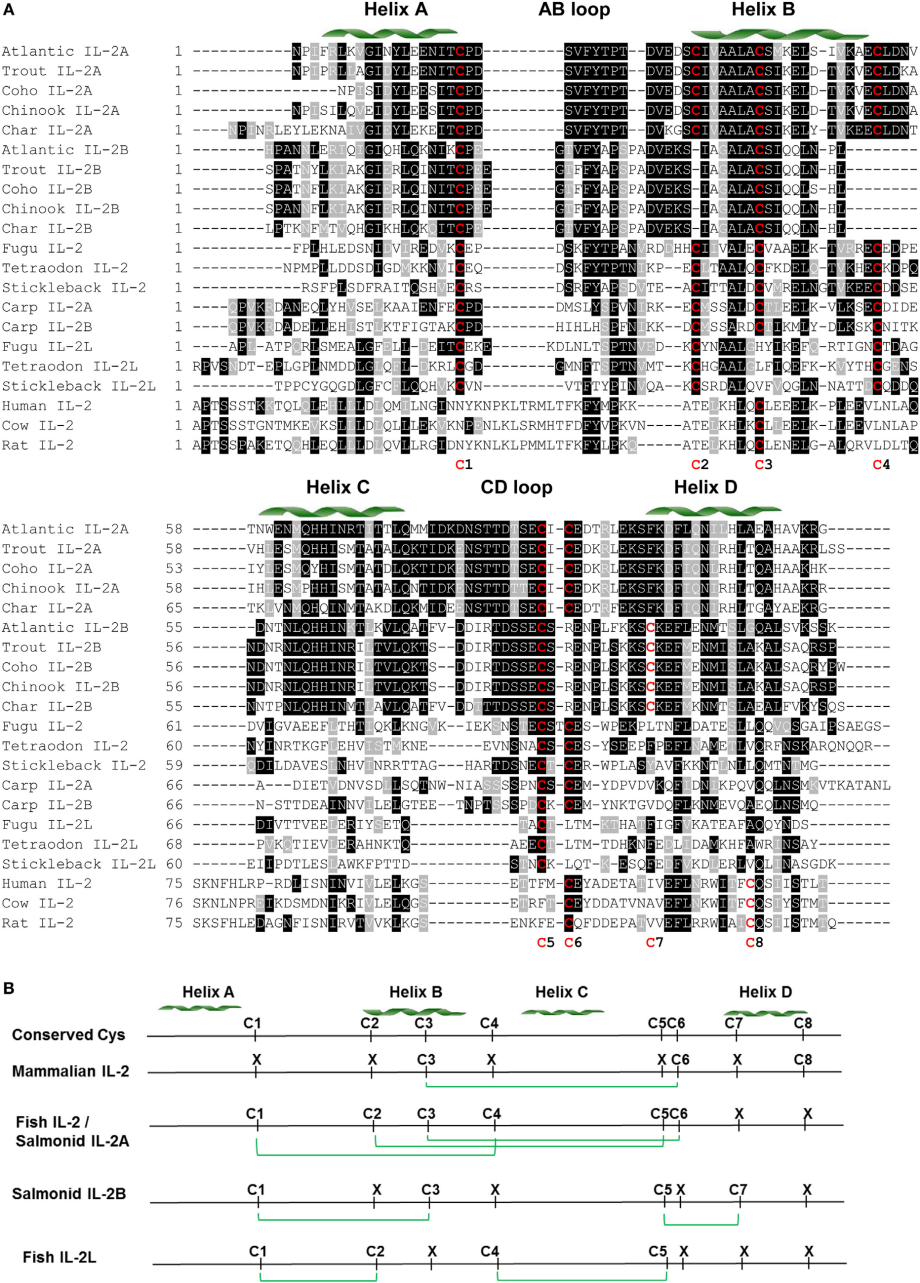
Amino acid (aa) sequence alignment of IL-2 mature peptides from salmonids and selected fish and mammalian species **(A)** and predicted potential intra-molecular disulfide bonds **(B)**. **(A)** The multiple alignment was produced using ClustalW, and conserved aa shaded using BOXSHADE (version 3.21). The four α helices (A–D) are indicated above the alignment. The conserved cysteine residues are in red and numbered at the bottom of the alignment (C1–8). The aa sequences and accession numbers are detailed in Supplementary Material: protein sequences. **(B)** Lineage- and paralog-specific conservation of cysteine residues in mammalian IL-2, salmonid IL-2A and other fish IL-2, and salmonid IL-2B and IL-2L is apparent. Cysteine residues potentially forming disulfide bonds are linked by green lines.

Each salmonid *IL2* gene has four to seven ATTTA mRNA instability motifs in the 3′-UTR, suggesting that salmonid *IL2* mRNA is unstable. Each salmonid *IL2* gene encodes for 135 −147 aa with a predicted signal peptide of 20 aa, a mature peptide of 115 −127 aa and a molecular weight of 12.8–14.5 kDa (Table S2 in Supplementary Material). The isoelectric point (pI) of the mature peptide of salmonid IL-2A is acidic (4.65–5.11) as seen in other fish species (Tables S2 and S4 in Supplementary Material). However, the pI of salmonid IL-2B is relatively high (6.79–7.87). One to four N-glycosylation sites can be predicted in each salmonid IL-2 peptide, with the site in the CD loop conserved in salmonid IL-2A (Figure 2A; Figure S10 in Supplementary Material).

There are six conserved cysteine residues in salmonid IL-2A but only four in IL-2B (Table S2 and Figure S10 in Supplementary Material). These cysteine residues are conserved in seven positions (C1–7) in a paralog-specific manner. IL-2A contains the first six cysteines (C1–6) potentially forming three intra-molecular disulfide bonds (C1/C4, C2/C5, and C3/C6, Figure S10 in Supplementary Material), as predicted using DISULFIND program (31). In addition to three conserved cysteine residues (C1, C3, and C5) present in IL-2A, IL-2B had a unique cysteine (C7). These four cysteine residues were predicted to form two distinct disulfide bonds (C1/C3 and C5/C7, Figure S10 in Supplementary Material).

A multiple alignment was produced using ClustalW (27) from the predicted mature peptide sequence of all salmonid IL-2A and IL-2B, other fish IL-2 and IL-2L, and selected mammalian IL-2 molecules (Figure 2). In general, there is high conservation in regions of the four helices but marked differences in the long AB and CD loops. There are conserved cysteine residues in eight positions (C1–8) over the alignment but none are conserved in all molecules and show lineage and paralog specificity. Mammalian IL-2 possesses three cysteine residues (C3, C6, and C8) that form a single disulfide bond (C3/C6) (Figure 2) important to stabilize its structure and bioactivity (45). Salmonid IL-2A and other fish IL-2, including the two common carp IL-2 isoforms, share the same six cysteine pattern (C1–6) that is predicted to form three disulfide bonds (C1/C4, C2/C5, and C3/C6) (Figure 2B). Salmonid IL-2B and other fish IL-2L all have four conserved cysteine residues potentially forming two disulfide bonds; however, the cysteine patterns are different. Salmonid IL-2B molecules possess C1, C3, C5, and C7, whereas IL-2L have C1, C2, C4, and C5 (Figure 2B).

#### Phylogenetic Tree Analysis of Salmonid *IL2*

**Figure 3:**
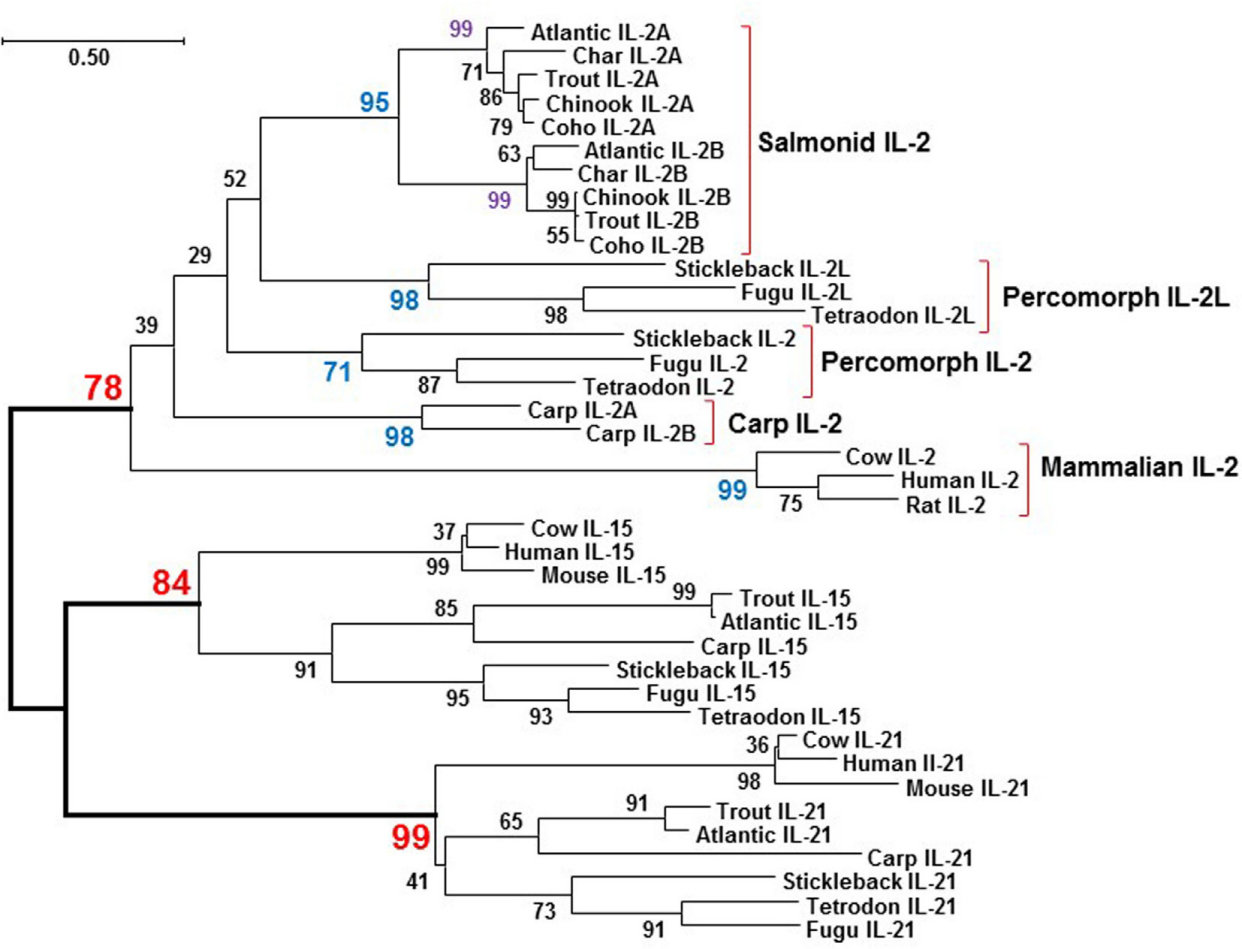
Phylogenetic tree analysis of salmonid IL-2, IL-2 and IL-2L from other fish species, and selected mammalian IL-2 molecules with the closely related γC cytokines IL-15 and IL-21. The phylogenetic tree was constructed using amino acid (aa) multiple alignments and the neighbor-joining method within the MEGA7.0 program (36). The percentage of replicate trees in which the associated taxa clustered together in the bootstrap test (10,000 replicates) is shown next to the branches. The evolutionary distances were computed using the JTT matrix-based method with all ambiguous positions removed for each sequence pair. The aa sequences and accession numbers are detailed in Supplementary Material: protein sequence.

The aa sequences of salmonid IL-2 orthologs, i.e., IL-2A or IL-2B, share high identity/similarity, e.g., salmonid IL-2B share 74.1–98.5%/80.4–99.3% aa identities/similarities. However, comparison of the salmonid IL-2 paralogs, i.e., IL2A vs IL-2B, revealed the aa sequence identities/similarities are lower, 38.9–43.0%/59.62–67.9%, similar to values for salmonid type I and type II TNFα isoforms (with 45.2–47.5% identities) that arose from the 3R teleost-wide WGD (41), and suggested a possible 3R origin. To clarify this, a neighbor-joining phylogenetic tree was produced from a multiple alignment of salmonid IL-2, other fish IL-2 and IL-2L, and mammalian IL-2 molecules, along with the IL-2 close relatives IL-15 and IL-21 from selected fish and mammals. All fish IL-2, IL-2L, IL-2A, and IL-2B molecules were grouped with mammalian IL-2 and separated from IL-15 and IL-21 from mammals and fish (Figure 3), suggesting that all fish IL-2-related molecules are indeed orthologs of mammalian IL-2. Salmonid IL-2A and IL-2B each formed an independent clade, which grouped together but was separate from other IL-2 molecules, a typical scenario for genes arising from the salmonid-specific 4R WGD (46). The two common carp IL-2 also grouped together suggesting a recent origin, perhaps from the carp-specific 4R WGD that occurred 5.6–11.3 million year ago (Mya, 44). The IL-2L, its gene only found in percomorphs neighboring the percomorph *IL2* (19) also formed an independent clade.

#### Synteny Analysis of *IL2* Loci in Rainbow Trout and Atlantic Salmon

**Figure 4:**
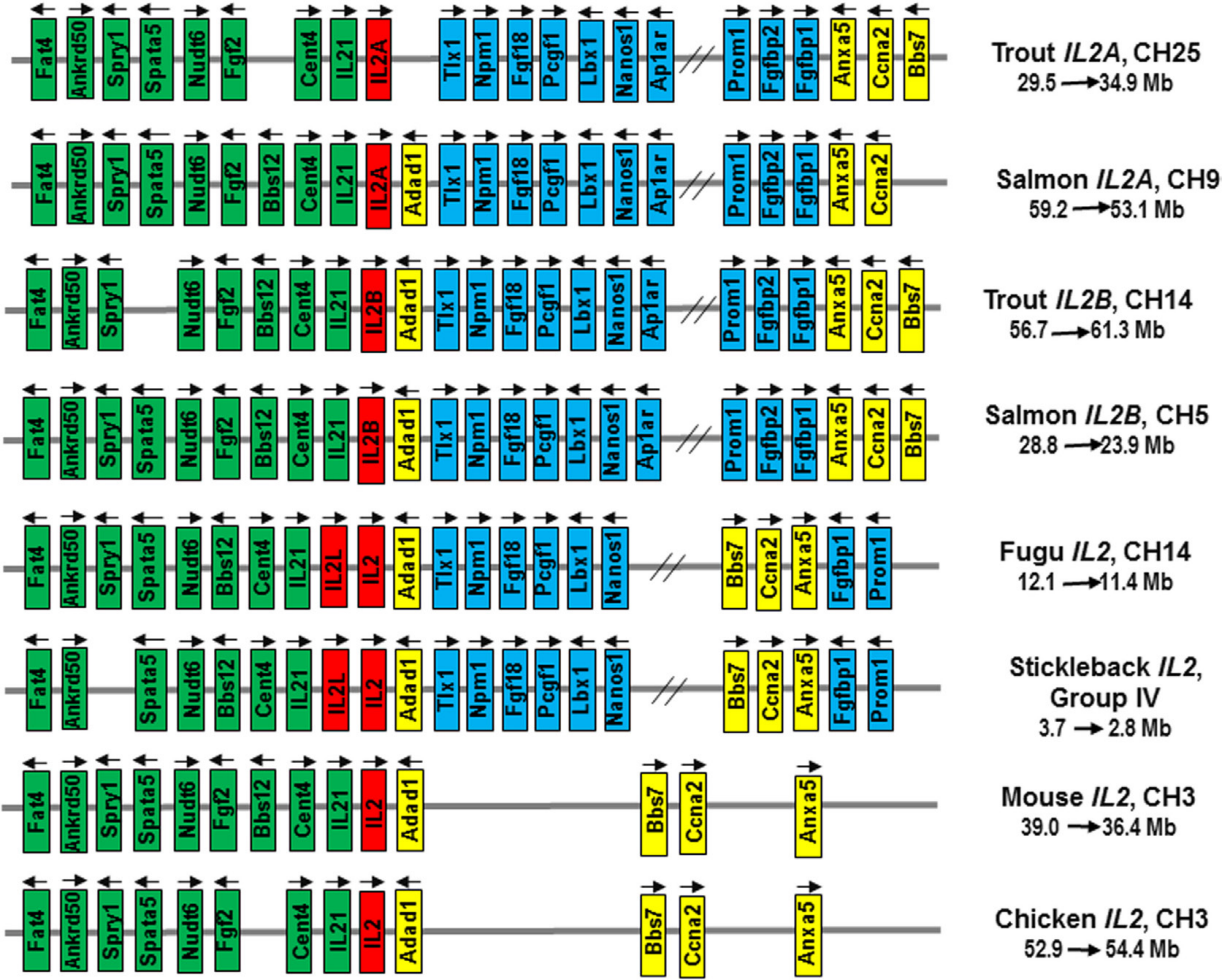
Gene synteny at the *IL2* loci in salmonids in comparison to other teleosts (Fugu, stickleback), mammals (mouse), and birds (chicken). The mouse and chicken *IL2* loci were analyzed using the Genomicus program (database version: 90.01). The information for salmonid and fugu *IL2* loci was extracted from NCBI reference genome sequences NC_035101 (Trout *IL2A*), NC_035090 (Trout *IL2B*), NC_027308 (Atlantic salmon *IL2A*), NC_027304 (Atlantic salmon *IL2B*), and NC_018903 (Fugu, *Takifugu rubripes*). The stickleback *Gasterosteus aculeatus IL2* loci was extracted from Ensembl database (https://www.ensembl.org/Gasterosteus_aculeatus/Location/View?r=groupIV:2800000-3700000). Arrows indicate transcriptional direction. *IL2* genes are in red. The genes syntenically conserved across vertebrates are in green (upstream of *IL2*) or yellow (downstream of *IL2*), and those conserved in teleosts only are in blue.

To clarify the origin of salmonid *IL2A* and *IL2B*, a synteny analysis was performed on the reference genome of rainbow trout, Atlantic salmon, fugu, stickleback, mouse, and chicken. The rainbow trout *IL2A* and *IL2B* were mapped to CH25 (Sequence ID: NC_035101) and CH14 (Sequence ID: NC_035090), respectively, while the Atlantic salmon *IL2A* and *IL2B* were mapped to CH9 (Sequence ID: NC_027308) and CH5 (Sequence ID: NC_027304), respectively. The *IL2* loci across vertebrates are syntenically conserved as shown in Figure 4. The genes (*Fat4, Ankrd50, Spry1, Spata5, Nudt6, Fgf2, Bbs12, Cetn2, IL21, Adad1, Bbs7, Ccna2*, and *Anxa5*) are syntenically conserved across all vertebrates with an insertion of genes (*Tlx1, Npm1, Fgf18, Pcgf1, Lbx1, Nanos1, Ap1ar, Prom1, Fgfbp1, Fgfbp2*, etc.) to one side of fish *IL2*. While the *IL2A* and *IL2B* are located in two homologous CH regions in both rainbow trout and Atlantic salmon, the *IL2* and *IL2L* genes in other teleosts are adjacent (19) within the syntenically conserved *IL2* loci (Figure 4). This chromosomal organization of *IL2* loci suggests that the divergent salmonid *IL2A* and *IL2B* arose from the salmonid-specific 4R WGD event, in contrast to other teleost *IL2* and *IL2L* that were due to local gene duplication.

### Comparative Tissue Transcript Analysis of *IL2* Paralogs in Rainbow Trout

**Figure 5:**
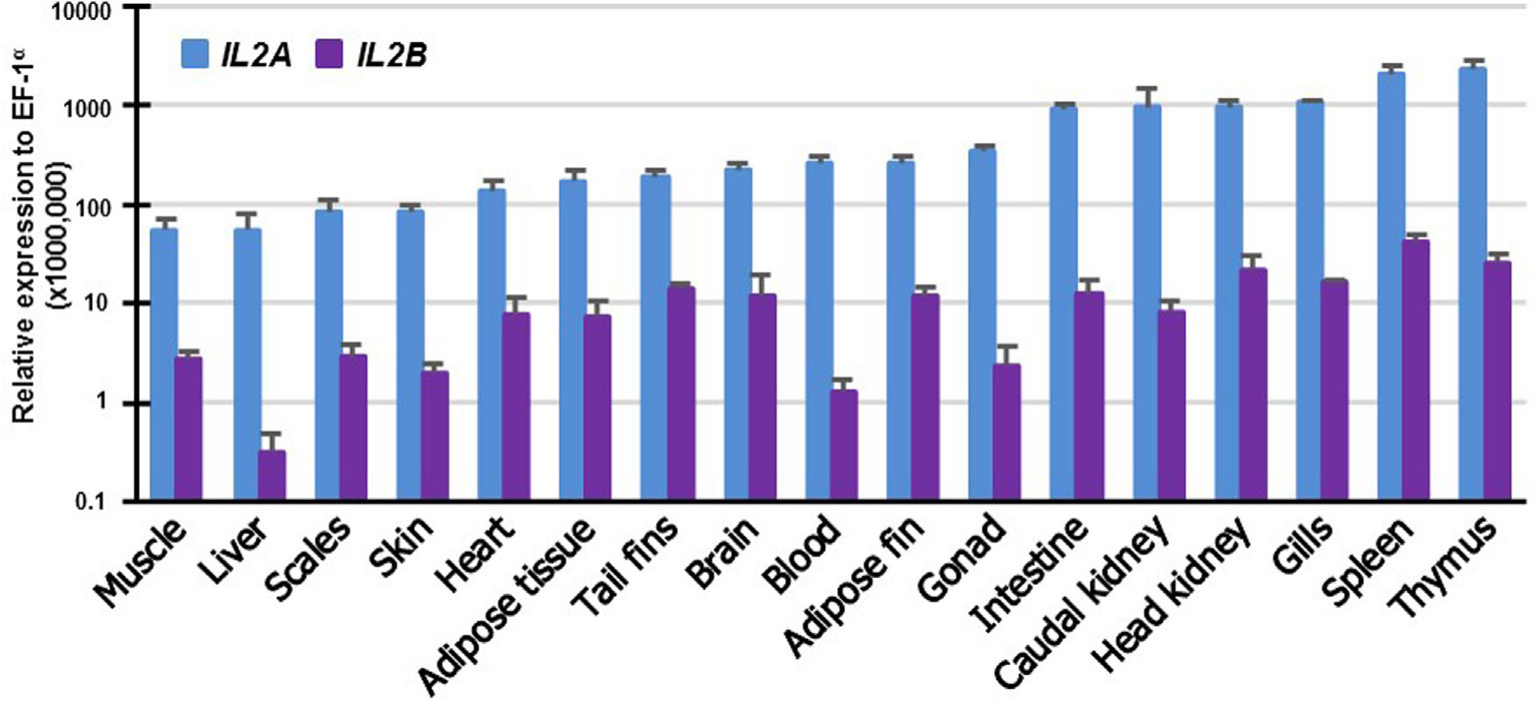
Tissue distribution of transcript expression of *IL2* paralogs in rainbow trout. The expression level of the two trout *IL2* paralogs was determined by real-time RT-PCR in 17 tissues from six fish. The transcript level was calculated using a serial dilution of references that contained equal molar amounts of the probes for each gene and was normalized against the expression level of *EF1α*. The results represent the average + SEM of six fish.

To gain insights into the potential function of the salmonid *IL2* paralogs, their expression was examined in 17 tissues of rainbow trout. The expression of both paralogs was detectable in all the tissues albeit at different levels. *IL2A* expression was highest in the immune organs thymus and spleen: high in gills, HK, caudal kidney and intestine, and low in muscle and liver (Figure 5). *IL2B* expression was also highest in immune organs (spleen, thymus, HK and gills) and lowest in liver. It is noteworthy that the expression of *IL2B* was lower than *IL2A* in all tissues examined (*p* ≤ 0.05, paired samples *T*-test).

### Modulation of *IL2* Paralog Expression in Rainbow Trout PBL by PHA, PMA, and CI

**Figure 6:**
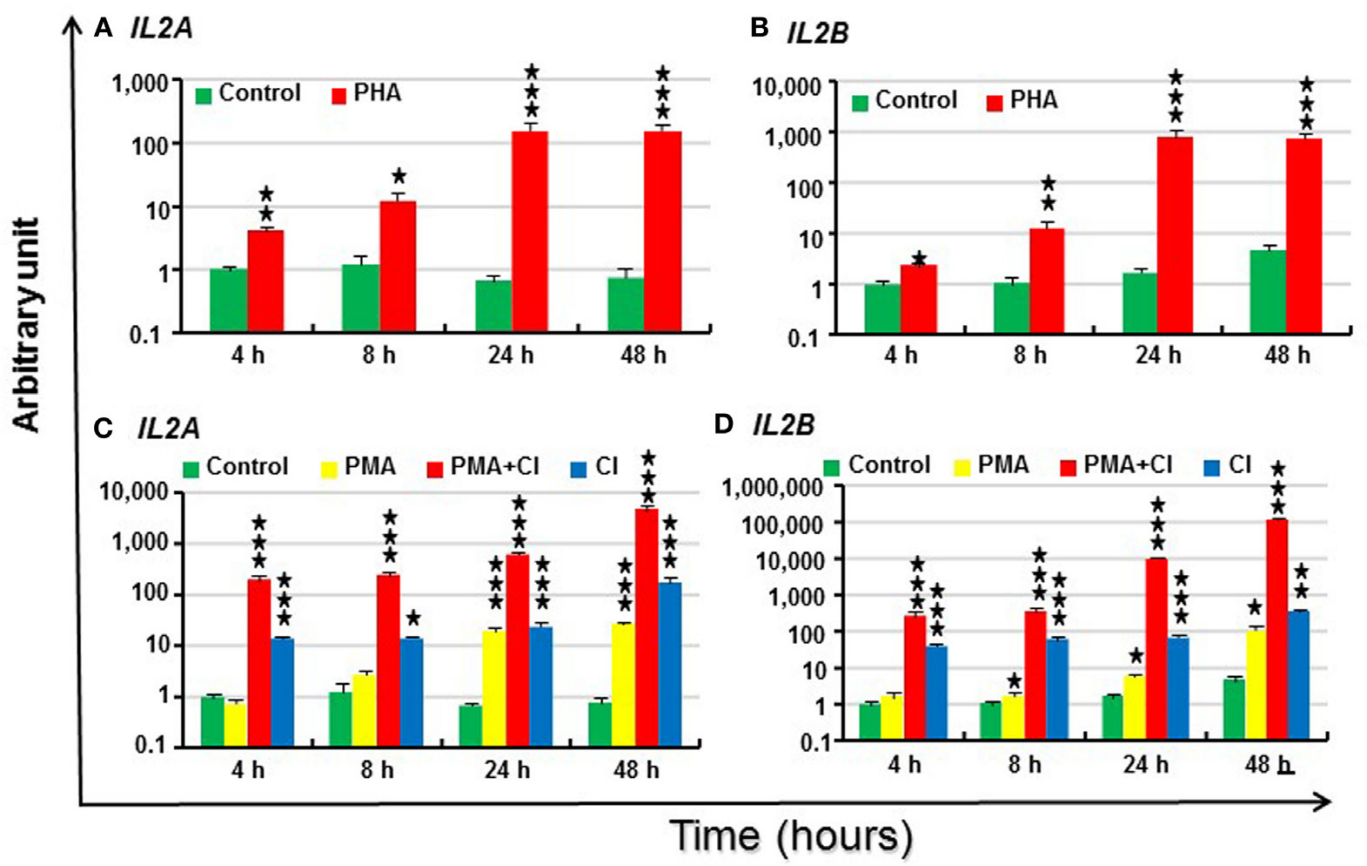
Modulation of *IL2* paralog expression in rainbow trout peripheral blood leukocytes (PBL) by phytohemagglutinin (PHA) **(A,B)**, phorbol 12-myristate 13-acetate (PMA) and calcium ionophore (CI) **(C,D)**. PBL freshly prepared from four fish were individually stimulated with PHA (10 µg/ml), PMA (50 ng/ml), CI A23187 (100 ng/ml), or a combination of PMA and CI for 4, 8, 24 and 48 h. The expression of *IL2* paralogs was measured by RT-qPCR. The mean (+SEM) relative expression is presented as arbitrary units where one unit equals the average expression level in control PBL at 4 h. The outcome of a paired samples *T*-test between stimulated samples and controls at the same time point is shown above the bars as *p ≤ 0.05, **p ≤ 0.01, and ***p ≤ 0.001. The expression levels in PMA + CI stimulated samples are significantly higher than either samples stimulated with PMA or CI alone at all the time points (*p* ≤ 0.05).

The modulation of trout *IL2* paralogs by the T cell activator PHA was next investigated in freshly prepared PBL. Trout *IL2A* and *IL2B* were lowly expressed constitutively in PBL as indicated by a ΔCP of control PBL at 4 h of 18.0 and 22.5, respectively (Table S3 in Supplementary Material). The expression of both *IL2* paralogs was induced by PHA from 4 to 48 h and peaked at 24 h (224-fold increase for *IL2A* and 457-fold for *IL2B*, Figures 6A,B).

Signaling *via* the TCR is believed to result in various biochemical events that include a rise in intracellular free calcium and activation (translocation) of protein kinase C. These two signals also can be generated by CI A23187 and by activators of protein kinase C, such as PMA (10). Thus, *IL2* expression in PBL was further investigated after stimulation with PMA and CI alone or in combination. Trout *IL2* was induced by CI from 4 to 48 h and reached the highest induction at 48 h (225-fold for *IL2A* and 77-fold for *IL2B*) (Figures 6C,D). PMA alone was a relatively weak inducer of *IL2* expression. It induced *IL2A* expression 28-fold at 24 h and 35-fold at 48 h, while *IL2B* expression was modulated from 8 h and reached a 24-fold increase at 48 h (Figures 6C,D). However, PMA and CI could synergize to upregulate *IL2* expression. Both paralogs had markedly higher expression in samples stimulated by the combination of PMA and CI from 4 to 48 h vs that seen in samples stimulated with PMA or CI alone (6,126-fold increase for *IL2A* and 25,207-fold for *IL2B* at 48 h) (Figures 6C,D).

### Modulation of *IL2* Paralog Expression by the MLR

**Figure 7:**
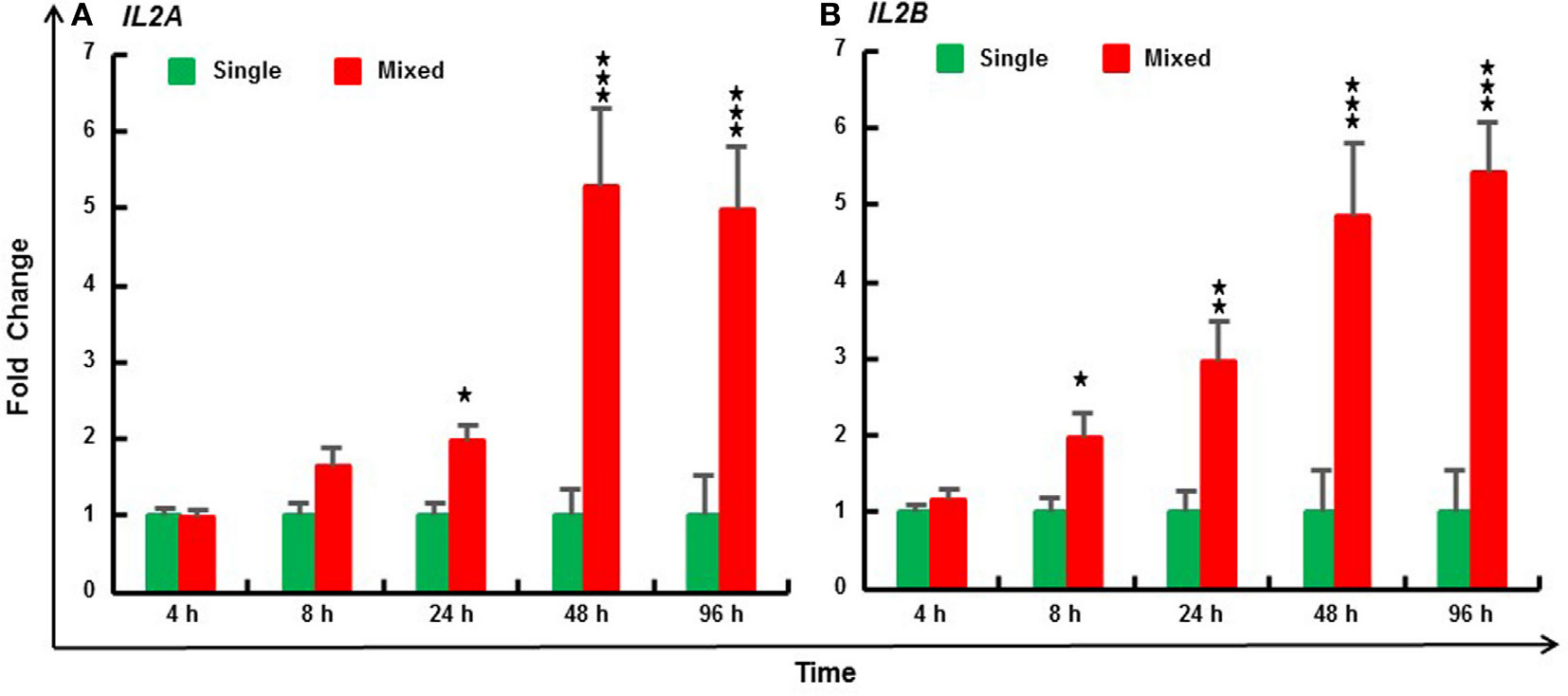
Mixed leukocyte reaction induced expression of IL2A **(A)** and IL2B **(B)** in rainbow trout peripheral blood leukocytes (PBL). PBL were prepared from 6 fish (1–6) and incubated at 20°C individually (single) or as a mix of three fish (mixed, 123, 234, 345, 456, 561, and 612) for 4, 8, 24, 48, and 96 h. The expression of *IL2* paralogs was measured by RT-qPCR. The data are presented as a mean (+SEM) fold change calculated by the average expression level of mixed PBL divided by that of single fish PBL at the same time point. The relative significance of a LSD *post hoc* test after a significant one-way analysis of variance between the mixed and single PBL at the same time point is shown above the bars as *p ≤ 0.05, **p ≤ 0.01, and ***p ≤ 0.001.

The MLR is due to T-cell activation by alloantigens presented by APCs. Three individuals were mixed to increase the magnitude and decrease the variation of the *in vitro* response (40). Compared with PBL cultured from individual fish, trout *IL2A* expression was significantly induced by the MLR from 24 to 96 h, and *IL2B* expression from 8 to 96 h (Figure 7). Similarly, MLR-induced *IL2* expression was also seen in HK cells (Figure S11 in Supplementary Material).

### Bioactivity of Trout Recombinant IL-2A and IL-2B in PBL

Recombinant trout IL-2A and IL-2B were expressed in *E. coli* after IPTG induction and purified under denaturing conditions (Figure S12 in Supplementary Material), with extensive washing with a buffer containing 1.5% Triton X-100 to remove LPS. The proteins were then refolded and re-purified. The purified IL-2 proteins, at up to 2 µg/ml, were ineffective at inducing the expression of TNF-α1, IL-1β, and COX-2, classic inflammatory genes that are known to be upregulated by LPS (41, 42) in the macrophage cell line RTS-11. When added to HK cells for 24 h, both proteins induced the expression of *IFNγ1* and *IFNγ2* from 2 to 500 ng/ml, and *TNFα2* from 20 to 500 ng/ml (Figure S13 in Supplementary Material). The expression of *TNFα1* was again not affected, suggesting that LPS contamination is negligible. The dose at 200 ng/ml of IL-2 isoforms induced a good response in HK cells and was used for analysis of IL-2 bioactivities in PBL in a time-course experiment to investigate the early (4 h), intermediate (8 and 24 h) and long-lasting (48 h) effects of IL-2 isoforms.

#### Modulation of Th1 Pathway Gene Expression by IL-2 Isoforms in Rainbow Trout PBL

**Figure 8:**
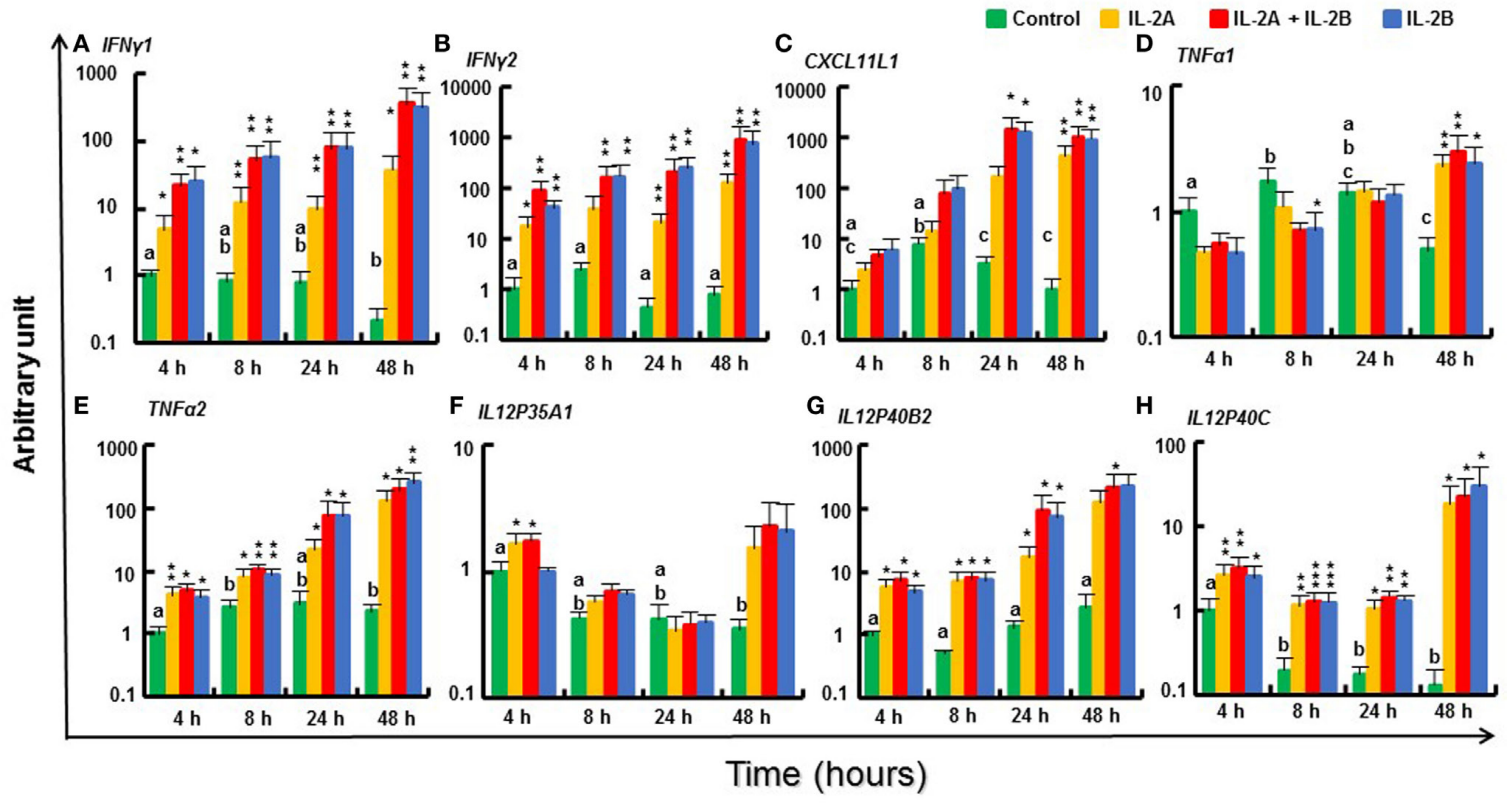
Modulation of T helper 1 pathway gene expression by IL-2 isoforms in peripheral blood leukocytes (PBL). Freshly prepared PBL from four fish were incubated with 200 ng/ml IL-2A, IL-2B, or both (IL-2A + IL-2B), or with medium alone as control for 4, 8, 24, and 48 h. The expression of *IFNγ1* **(A)**, *IFNγ2* **(B)**, *CXCL11L1* **(C)**, *TNFα1* **(D)**, *TNFα2* **(E)**, *IL12P35A1* **(F)**, *IL12P40B2* **(G)** and *IL12P40C* **(H)** was quantified as in Figure 6. The data are presented as mean (+SEM) arbitrary units where one unit equals the average expression level in the control samples at 4 h. Significant results of a paired samples *T*-test between the stimulated samples and time-matched controls are shown above the bars as *p ≤ 0.05, **p ≤ 0.01, and ***p ≤ 0.001. Different letters over the control bars indicate significant differences over time in the unstimulated cells (*p* ≤ 0.05). The expression levels in samples treated with IL-2B or IL-2A + IL-2B show no differences but are higher than IL-2A-treated samples for IFNγ1 at 8, 24, and 48 h, IFNγ2 at 8 and 48 h, and CXCL11L1 at 24 h (*p* ≤ 0.05).

IL-12 and IFNγ are critical cytokines that initiate the downstream signaling cascade *via* their receptors and the master transcription factor T-bet to develop Th1 cells to secrete IFNγ, TNFα, and IL-2 (3). Two *IFNγ* genes (47), *IFNγ1* and *IFNγ2*, present in salmonids due to the salmonid WGD were both rapidly induced by both IL-2 isoforms, alone or in combination, from 4 h until 48 h (Figures 8A,B). Similarly, the expression of *TNFα2, IL12P40B2*, and *IL12P40C* (48, 49) was also induced from 4 to 48 h (Figures 8E,G,H). However, *TNFα1* and *TNFα3* expression was less responsive, with relatively higher induction levels seen at 24 h (TNFα3 only) and 48 h (Figure 8D; Figure S14A in Supplementary Material). Of the other TNF family members studied, *FasL* was refractory to IL-2 isoform stimulation but *CD40L* was induced to a small extent at 4 and 48 h (Figures S14B,C in Supplementary Material). Within the IL-12 family, the expression of the alpha chains, *IL12P35A2* (Δcp = 20.2), *IL23P19* [(50), Δcp = 21.8] and *IL27A* [(51), Δcp = 19.7] was low in PBL and not significantly modulated by IL-2 isoforms. The expression of *IL12P35A1* was higher (Δcp = 15.1) and induced at 4 h by IL-2A but not by IL-2B (Figure 8F). No induction was seen for *IL12P40B1* and *EBI3* (Figures S14F,G in Supplementary Material). *CXCL11L1* (52), a downstream effector of IFNγ signaling, was upregulated by the IL-2 isoforms at late time points (Figure 8C). *IFNγR2* (53) expression was induced to a small extent in IL-2A (24–48 h) and IL-2B (8–48 h) stimulated PBL; however, *IFNγR1* and *T-bet* (54) were refractory (Figures S14D,E,H in Supplementary Material). *IFNγ* can be induced by IL-15 (43), IL-18 (55), and IL-21 (37). None of these cytokines was modulated in PBL by the IL-2 isoforms except for a downregulation of *IL15* at 8 h by both isoforms and a downregulation of *IL21* at 24 h by IL-2A (Figures S14I–K in Supplementary Material).

The ability of the trout IL-2 isoforms to modulate the expression of Th1 pathway genes was generally overlapping, but subtle differences were observed. The expression of *IFNγ1, IFNγ2, CXCL11L1*, and *TNFα3* was higher in IL-2B stimulated PBL at some time points. By contrast, the expression of *IL12P35A1* was induced only by IL-2A (Figure 8). The modulated gene expression seen with the combination of the two isoforms was mostly IL-2B-like with the exception of *IL12P35A1* that is IL-2A like (Figure 8). These patterns of activity suggest that the IL-2 isoforms may signal *via* the same cell surface receptors independently. The signal intensity is likely determined by the presence of specific combinations of receptor subunits and their affinity to the IL-2 isoforms that will be discussed later.

#### Modulation of Th2 Pathway Gene Expression by IL-2 Isoforms in Rainbow Trout PBL

**Figure 9:**
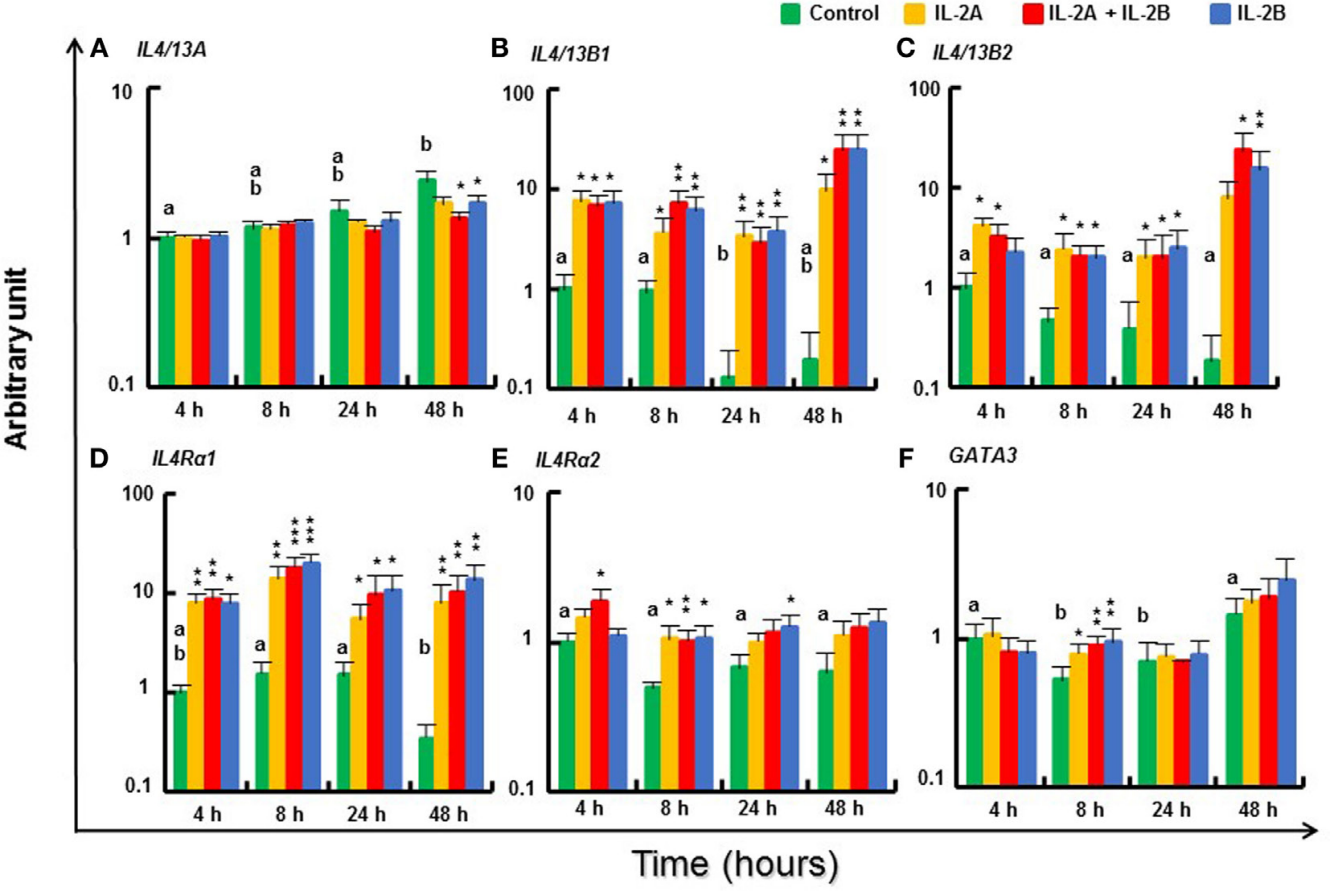
Modulation of T helper 2 pathway gene expression by IL-2 isoforms in peripheral blood leukocytes (PBL). Freshly prepared PBL from four fish were stimulated with IL-2A, IL-2B, IL-2A + IL-2B, or with medium alone as control. The expression of *IL4/13A* **(A)**, *IL4/13B1* **(B)**, *IL4/13B2* **(C)**, *IL4Rα1* **(D)**, *IL4Rα2* **(E)** and *GATA3* **(F)** was quantified and presented as in Figure 8. Significant results of a paired samples *T*-test between the stimulated samples and time-matched controls are shown above the bars as *p ≤ 0.05, **p ≤ 0.01, and ***p ≤ 0.001. Different letters over the control bars indicate significant differences over time in the unstimulated cells (*p* ≤ 0.05). The expression levels in samples treated with IL-2A + IL-2B are higher than with IL-2A for IL-4/13B1 and IL4Rα1 at 8 h, IL-4/13B2 at 48 h, and higher than either IL-2A or IL-2B alone for IL4Rα2 at 4 h (*p* ≤ 0.05).

The expression of Th2 cytokines *IL4/13B1* and *IL4/13B2*, and their receptor *IL4Rα1* (44) was upregulated in stimulated PBL from 4 to 48 h with no difference between IL-2B and the combination of IL-2A and IL-2B (Figures 9B–D). However, at some time points, e.g., *IL4/13B1* and *IL4Rα* at 8 h, and *IL4/13B2* at 48 h, the induced expression was higher in PBL stimulated by both IL-2A and IL-2B than by either alone. In agreement with previous studies showing IL4/13A expression was less responsive to T cell stimulation (24), *IL4/13A* expression was refractory to both isoforms from 4 to 24 h and showed a small decrease at 48 h in PBL stimulated with IL-2B or both isoforms (Figure 9A). The expression of *IL4Rα2*, the abundant *IL4Rα* isoform (Δcp of 11.4 compared with 14.4 of *IL4Rα1*, Table S3 in Supplementary Material) was also upregulated to a small extent at 4 and 8 h by the combination of IL-2A and IL-2B, at 8 h by IL-2A, and at 8 and 24 h by IL-2B (Figure 9E). The expression of the Th2 master transcription factor *GATA3* was maintained at 8 h after IL-2 stimulation when its expression in unstimulated PBL had decreased, but no difference was seen at other time points (Figure 9F).

#### Modulation of Th17 Pathway Gene Expression by IL-2 Isoforms in Rainbow Trout PBL

*IL17A/F1A, IL17A/F2A, IL17A/F3* (25), and *IL22* (56) genes were lowly expressed in PBL with a Δcp of 19.7, 21.3, 20.4, and 22.6, respectively (Table S3 in Supplementary Material). Their expression was not modulated by either isoform alone or together (Figures S15A–C in Supplementary Material). *IL17C1, IL17C2* (57), and *IL17D* were also lowly expressed in PBL (Table S3 in Supplementary Material) and are not described further (Figure S15D in Supplementary Material). The expression of the Th17 master transcription factor *RORγ* (58) was slightly downregulated at 8 h by IL-2A and at 48 h in the presence of both IL-2 isoforms (Figure S15E in Supplementary Material). *IL17Rα* (59) was refractory (Figure S15F in Supplementary Material).

#### Modulation of Pro-Inflammatory Gene Expression by IL-2 Isoforms in Rainbow Trout PBL

In general, the effects of the IL-2 isoforms on the expression of pro-inflammatory cytokines (*IL1β, IL6, IL8, IL11, IL34*, and *M17*) were minor (Figure S16 in Supplementary Material). Three *IL1β* are present in rainbow trout (60). *IL1β2* was lowly expressed in PBLs (Δcp = 21.8) and refractory. *IL1β1* expression was inhibited at 8 h by IL-2B but a small increase of *IL1β3* was seen at 4 h in the presence of both isoforms (Figures S16A,B in Supplementary Material). nIL1Fm (61) expression was also increased to a small extent at 4 h by IL-2 isoforms alone and maintained to a higher level at 48 h with all three treatments (Figure S16C in Supplementary Material). *IL6* expression was refractory, while *IL8* (62) expression was inhibited at 4 h by both IL-2 isoforms alone, and *IL11* (63) was inhibited at 8 h by their combination (Figures S16D–F in Supplementary Material). The expression of *M17* (64) was increased at 8 and 48 h by all three treatments (Figure S16G in Supplementary Material), and a small increase in *IL34* (65) expression at 24 h was induced by IL-2B or both isoforms (Figure S16H in Supplementary Material). Relatively higher-level expression was seen for *M17* at 4 h and *IL34* at 24 and 48 h in PBL stimulated with IL-2B vs IL-2A (Figures S16G,H in Supplementary Material).

#### Modulation of Treg Pathway Gene Expression by IL-2 Isoforms in Rainbow Trout PBL

**Figure 10:**
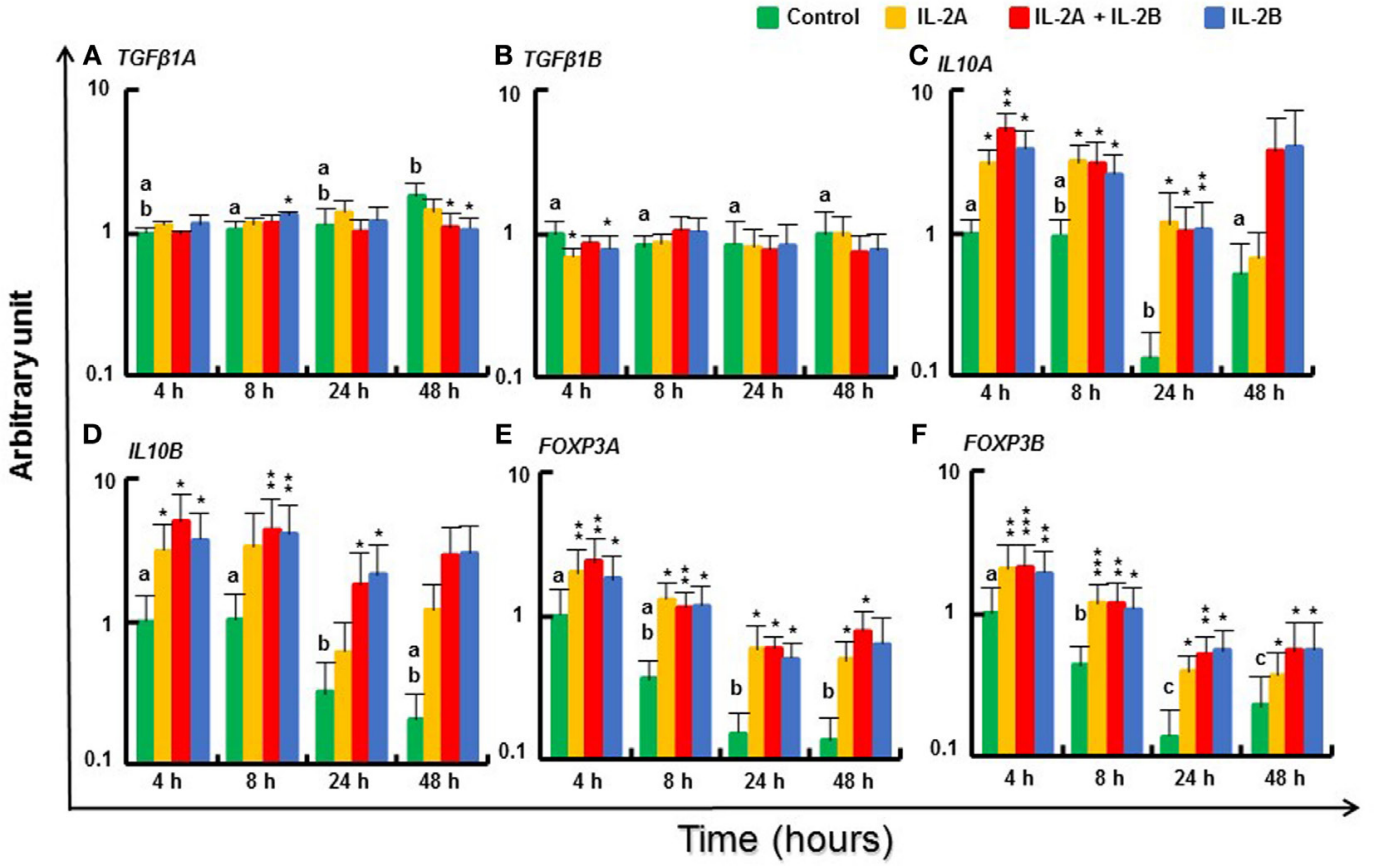
Modulation of regulatory pathway gene expression by IL-2 isoforms in peripheral blood leukocytes (PBL). Freshly prepared PBL from four fish were stimulated with IL-2A, IL-2B, IL-2A + IL-2B, or with medium alone as control. The expression of *TGFβ1A* **(A)**, *TGFβ1B* **(B)**, *IL10A* **(C)**, *IL10B* **(D)**, *FOXP3A* **(E)** and *FOXP3B* **(F)** was quantified and presented as in Figure 8. Significant results of a paired samples *T*-test between the stimulated samples and time-matched controls are shown above the bars as *p ≤ 0.05, **p ≤ 0.01, and ***p ≤ 0.001. Different letters over the control bars indicate significant differences over time in the unstimulated cells (*p* ≤ 0.05). The expression levels in samples treated with IL-2A + IL-2B are higher than with IL-2A for IL10A and FOXP3A at 4 h, and IL10B at 24 h, and higher than with IL-2B for IL10B at 4 h (*p* ≤ 0.05).

Small changes in expression of regulatory pathway genes were seen at some time points (Figure 10). *TGFβ1A* expression was increased to a small extent at 8 h by IL-2B but was downregulated at 48 h by IL-2B or IL-2B + IL-2A (Figure 10A). *TGFβ1B* (66) was also downregulated, at 4 h by both IL-2 isoforms (Figure 10B). However, *IL10A* and *IL10B* (67) were induced from 4 to 24 h by both IL-2 isoforms alone and together (Figures 10C,D). Interesting, the expression of the master transcription factors for Treg cell development, *FOXP3A* and *FOXP3B* (68), was increased from 4 to 48 h by all three treatments (Figures 10E,F).

#### Modulation of IL2 and IL2R Gene Expression by IL-2 Isoforms in Rainbow Trout PBL

**Figure 11:**
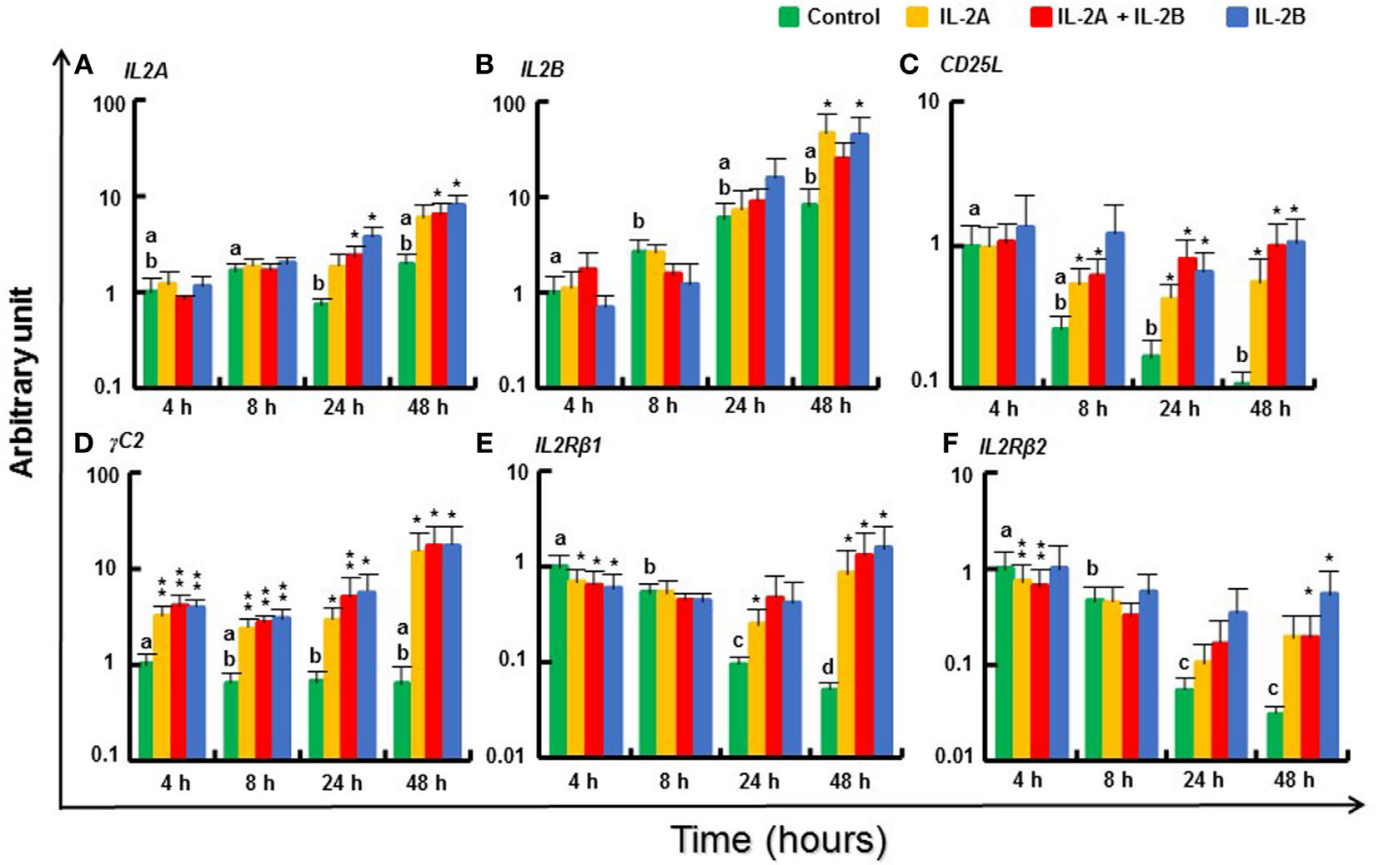
Modulation of *IL2* and *IL2R* gene expression by IL-2 isoforms in peripheral blood leukocytes (PBL). Freshly prepared PBL from four fish were stimulated with IL-2A, IL-2B, IL-2A + IL-2B, or with medium alone as control. The expression of *IL2A* **(A)**, *IL2B* **(B)**, *CD25L* **(C)**, *γC2* **(D)**, *IL2Rβ1* **(E)** and *IL2Rβ2* **(F)** was quantified and presented as in Figure 8. Significant results of a paired samples *T*-test between the stimulated samples and time-matched controls are shown above the bars as *p ≤ 0.05, **p ≤ 0.01, and ***p ≤ 0.001. Different letters over the control bars indicate significant differences over time in the unstimulated cells (*p* ≤ 0.05). The expression levels in samples treated with IL-2B are higher than with IL-2A for IL2A at 24 h, and IL2Rβ2 at 48 h, but lower than with IL-2A for IL2Rβ1 at 4 h (*p* ≤ 0.05).

*IL2A* was weakly induced at 24 and 48 h by IL-2B or the combination of IL-2A and IL-2B (Figure 11A). *IL2B* was also increased at 48 h by IL-2A or IL-2B alone (Figure 11B). The putative *IL2Ra CD25L* expression decreased over time *in vitro* but was sustained in the presence of IL-2 isoforms (Figure 11C). *γC1*, the broadly and abundantly expressed isoform, was only weakly induced at 4 and 8 h by IL-2B (Figure S14L in Supplementary Material), whereas *γC2*, that is less expressed constitutively (Δcp = 12.6 vs 5.6 of *γC1*, Table S3 in Supplementary Material) but more inducible by T-cell stimulants (13), was highly induced from 4 to 48 h by both IL-2 isoforms alone or together (Figure 11D). In contrast, the expression of *IL2Rβ1* was downregulated at 4 h by both IL-2 isoforms (alone or together) but maintained at higher levels in the presence of IL-2 when a decrease was seen in the control cells at later timings (Figure 11E). A similar expression pattern was observed for *IL2Rβ2* (Figure 11F).

#### Modulation of T Cell Marker Gene Expression by IL-2 Isoforms in Rainbow Trout PBL

**Figure 12:**
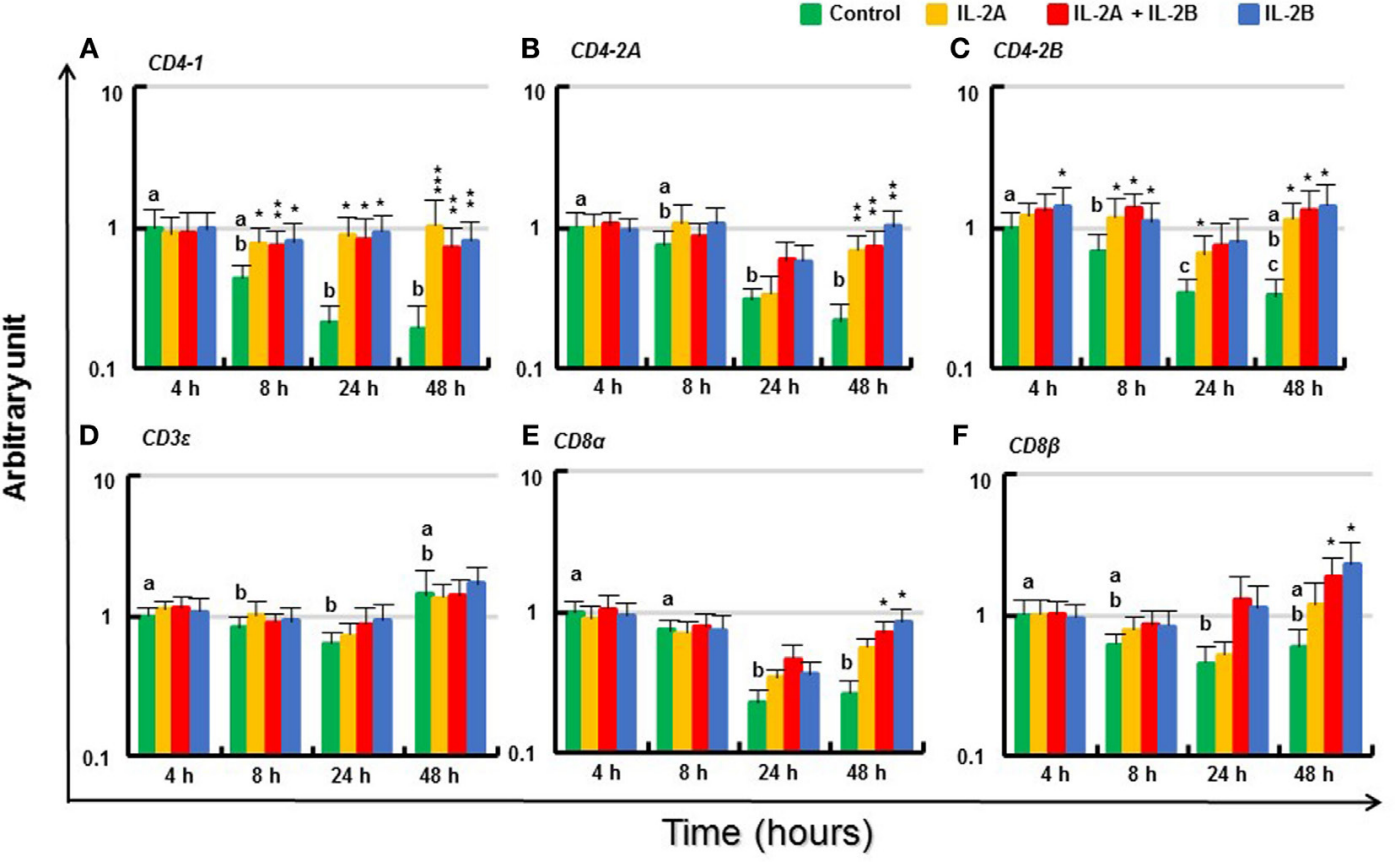
Modulation of T cell marker gene expression by IL-2 isoforms in peripheral blood leukocytes (PBL). Freshly prepared PBL from four fish were stimulated with IL-2A, IL-2B, IL-2A + IL-2B, or with medium alone as control. The expression of *CD4-1* **(A)**, *CD4-2A* **(B)**, *CD4-2B* **(C)**, *CD3ε* **(D)**, *CD8α* **(E)** and *CD8β* **(F)** was quantified and presented as in Figure 8. Significant results of a paired samples *T*-test between the stimulated samples and time-matched controls are shown above the bars as *p ≤ 0.05, **p ≤ 0.01, and ***p ≤ 0.001. Different letters over the control bars indicate significant differences over time in the unstimulated cells (*p* ≤ 0.05). The expression levels in samples treated with IL-2B or IL-2A + IL-2B are higher than IL-2A-treated samples for CD8α and CD8β at 48 h (*p* ≤ 0.05).

The expression of T cell markers (*CD4-1, CD4-2A, CD4-2B, CD3ε, CD8α*, and *CD8β*) in PBL *in vitro* without IL-2 decreased over time (Figure 12). Although there was no induction of these genes at 4 h (except for a small increase in *CD4-2B* by IL-2B), the expression of these T cell markers was maintained at almost constant levels by IL-2. Thus, the expression of CD4-1 was higher from 8 to 48 h in PBL treated with IL-2 compared with respective controls. This was also seen for *CD4-2A* and *CD4-2B*, particularly at 48 h (Figures 12A–C). The effect on *CD3ε* expression was less apparent but higher levels of *CD8α* and *CD8β* were seen at 48 h in PBL treated with IL-2B or IL-2A plus IL-2B (Figures 12D–F). The induction of *CD4-2A* at 24 h, and *CD8α* and *CD8β* at 48 h was greater in PBL treated with IL-2B or the combination of IL-2A and IL-2B.

#### Modulation of Chemokine Receptor Gene Expression by IL-2 Isoforms in Rainbow Trout PBL

**Figure 13:**
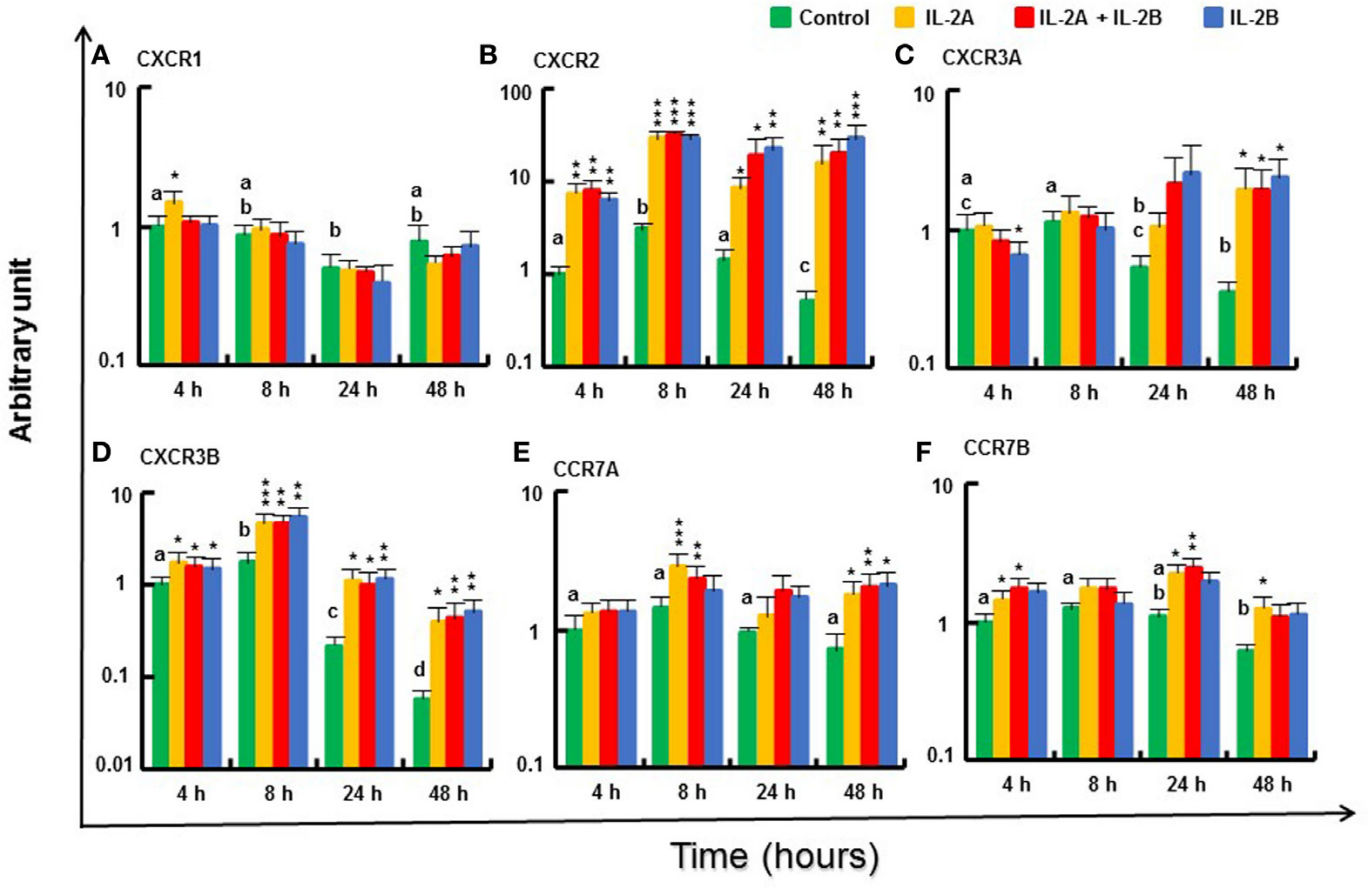
Modulation of chemokine receptor gene expression by IL-2 isoforms in peripheral blood leukocytes (PBL). Freshly prepared PBL from four fish were stimulated with IL-2A, IL-2B, IL-2A + IL-2B, or with medium alone as control. The expression of *CXCR1* **(A)**, *CXCR2* **(B)**, *CXCR3A* **(C)**, *CXCR3B* **(D)**, *CCR7A* **(E)** and *CCR7B* **(F)** was quantified and presented as in Figure 8. Significant results of a paired samples *T*-test between the stimulated samples and time-matched controls are shown above the bars as *p ≤ 0.05, **p ≤ 0.01, and ***p ≤ 0.001. Different letters over the control bars indicate significant differences over time in the unstimulated cells (*p* ≤ 0.05). The expression levels in samples treated with IL-2A are higher than with IL-2B for CXCR1 and CXCR3A at 4 h and CCR7A at 8 h, but lower for CXCR2 than with IL-2B or IL-2A + IL-2B at 24 h, and IL-2B at 48 h (*p* ≤ 0.05).

The expression of the chemokine receptors, such as *CXCR2* and *CXCR3B* (69), was increased in PBL from 4 to 48 h by IL-2A and IL-2B treatment (alone or in combination) (Figures 13B,D). A small induction was also seen for *CXCR1* at 4 h in PBL treated with IL-2A alone (Figure 13A). *CXCR3A* expression was downregulated at 4 h by IL-2B treatment but was sustained at higher levels in IL-2 treated PBL at 48 h when it was decreased in control cells (Figure 13C). A small induction of the expression of *CCR7A* was seen at 8 and 48 h, and *CCR7B* at 4, 24, and 48 h mainly by IL-2A or the combination of IL-2A and IL-2B (Figures 13E,F). The expression of *CXCR1* and *CXCR3A* at 4 and 8 h, and *CCR7A* and *CCR7B* at 8 h was higher in IL-2A-treated PBL. By contrast, the expression of *CXCR2* at 24 and 48 h was higher in IL-2B-treated PBL (Figure 13).

### IL-2 Isoforms Differentially Modulate Cathelicidin Gene Expression and Promote Phagocytosis of PBL

**Figure 14:**
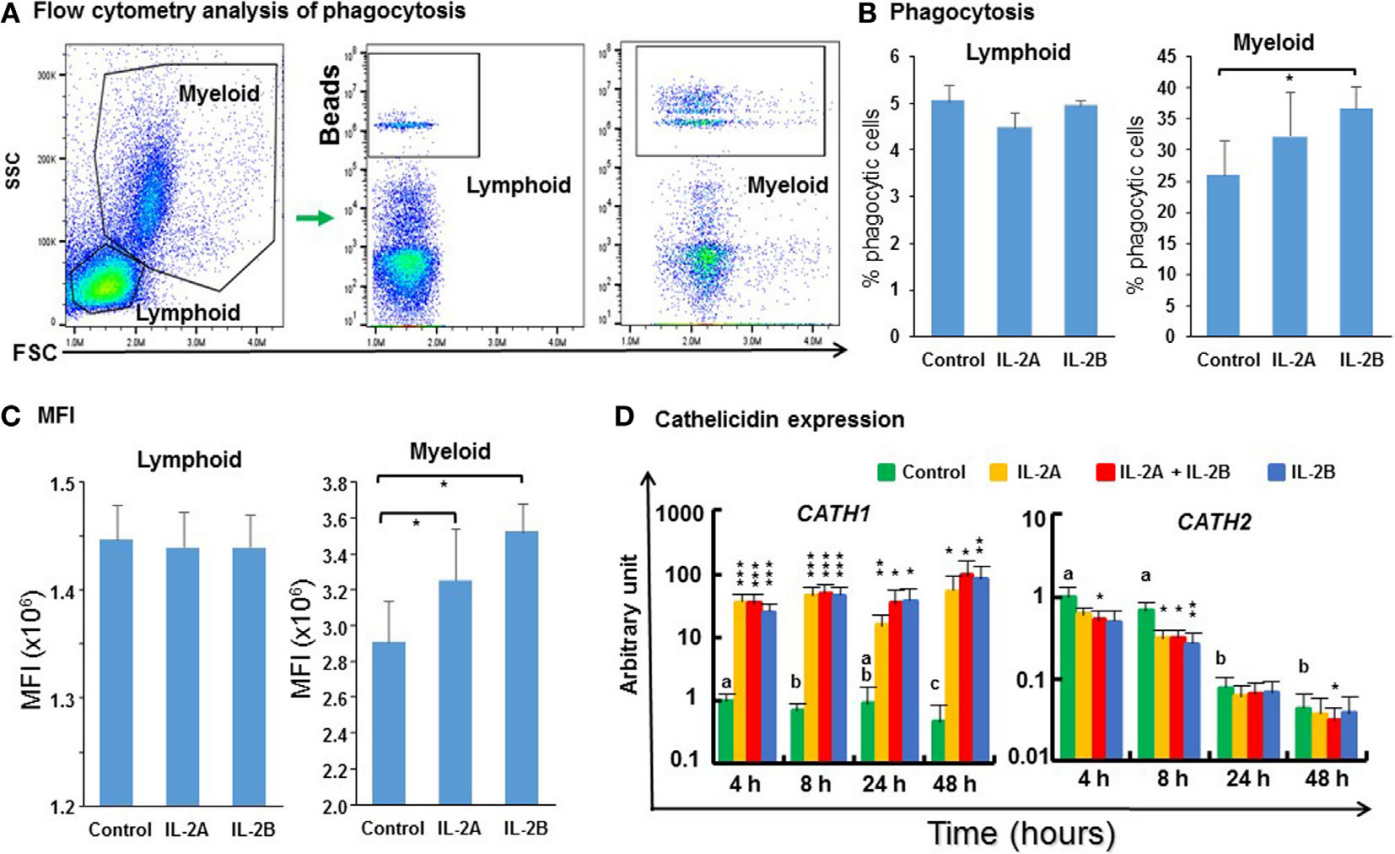
Flow cytometry analysis of phagocytosis **(A)**, percentage of phagocytic cells **(B)**, mean fluorescence intensity (MFI) of phagocytic cells **(C)**, and cathelicidin gene expression modulated by IL-2 isoforms in peripheral blood leukocytes (PBL) **(D)**. **(A)** Trout PBL were incubated with IL-2A, IL-2B, or medium alone as control for 24 h. PBL were then incubated with 1.0-µm fluorescent beads for 3 h and analyzed by flow cytometry. Typical results from a single fish are shown. **(B)** The percentage of phagocytic leukocytes in lymphoid and myeloid gates. The results are presented as the average + SEM of three fish. “*” indicates significant differences (*p* ≤ 0.5) of a paired samples *T*-test. **(C)** The MFI of phagocytic lymphoid and myeloid cells. The results are presented as the average + SEM of three fish. “*” indicates significant differences (*p* ≤ 0.5) of a paired samples *T*-test. **(D)** Freshly prepared PBL were stimulated with IL-2A, IL-2B, IL-2A + IL-2B, or with medium alone as control for 4, 8, 24 and 48 h, and the expression of *CATH1* and *CATH2* quantified. The results are presented as the average + SEM from four fish. Significant results of a paired samples *T*-test between the stimulated samples and time-matched controls are shown above the bars as: *p ≤ 0.05, **p ≤ 0.01, and ***p ≤ 0.001. Different letters over the control bars indicate significant differences over time in the unstimulated cells (*p* ≤ 0.05).

The phagocytic activity of PBL cultured *in vitro* with/without IL-2 isoforms was analyzed by flow cytometry using fluorescent beads (Figure 14A). 5% of the lymphoid cells were phagocytic in control PBL. This percentage was not affected by IL-2 treatment (Figure 14B). 26% of the myeloid cells were phagocytic in control PBL. This percentage was increased significantly to 37% by IL-2B stimulation (Figure 14B) but the increase seen with IL-2A treatment was not statistically significant (*p* = 0.07). In addition, more cells ingested more than one bead in the IL-2-stimulated samples (Figure 14A). Hence, the mean fluorescence intensity (MFI) of myeloid cells was increased significantly from 2.9 × 10^6^ in control samples to 3.2 × 10^6^ and 3.5 × 10^6^ in IL-2A and IL-2B stimulated samples, respectively (Figure 14C). The MFI was not altered in phagocytic lymphoid cells after IL-2 treatment. Importantly, the expression of *cathelicidin* (*CATH*)-*1*, known to enhance phagocytic activity in rainbow trout (70) was upregulated from 4 to 48 h by both IL-2 isoforms alone or together (Figure 14D). In contrast, the expression of *CATH2* was downregulated by IL-2 isoforms, when used individually at 8 h or together at 4, 8, and 48 h (Figure 14D). The differential modulation of *CATH1* and *CATH2* expression has also been seen with other cytokines, e.g., IL-4/13 (24), IL-6 (42), and TNFα (41) in rainbow trout.

### Recombinant Trout IL-2 Isoforms Promote Growth of PBL

**Figure 15:**
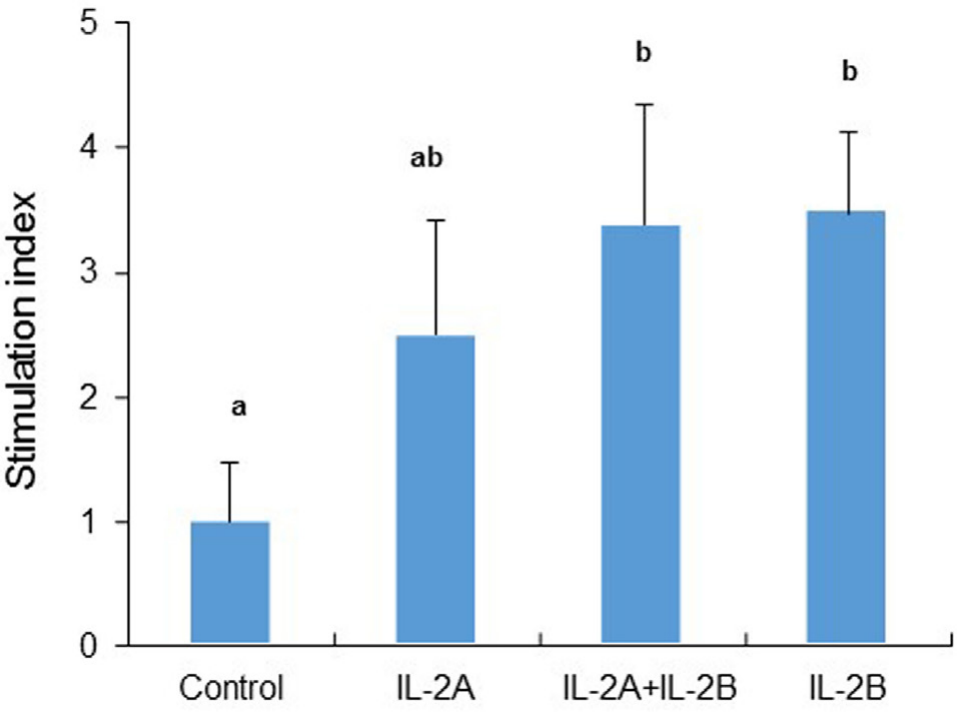
Rainbow trout IL-2 isoforms promote proliferation in peripheral blood leukocytes (PBL). Freshly prepared PBL from four fish were incubated with IL-2A and IL-2B, or with medium alone as control in triplicate for 3 days. BrdU was added 20 h before incorporation of BrdU was detected by ELISA. The data are presented as the average (+SEM) stimulation index, calculated as the OD450 of IL-2-treated cells divided by that of untreated samples. Different letters over bars indicate significant differences (*p* ≤ 0.05, paired samples *T*-test).

Mammalian IL-2 is a lymphocyte growth factor and so we examined the proliferation of PBL after stimulation with IL-2 isoforms. BrdU incorporation was increased in PBL treated with IL-2B alone and IL-2A + IL-2B (Figure 15). While no significant increase was seen using IL-2A-treated PBL, an intermediate level of proliferation was present that did not differ from the IL-2B treated cells.

## Discussion

In this study, two divergent *IL2* paralogs that arose from the salmonid WGD have been characterized molecularly in five salmonid species for which a sequenced genome is available. Expression and bioactivity analysis of these two *IL2* paralogs was then undertaken in one of these species, the rainbow trout *O. mykiss*. Our results suggest that IL-2 is an important T cell factor that regulates Th1 and Th2 pathways in fish and shed light on lineage-specific expansion, evolution, and functional divergence of *IL2* orthologs and paralogs.

### Lineage-Specific Expansion, Functional Divergence, and Convergent Evolution of *IL2*

Vertebrate animals emerged from the invertebrates approximately 500 Mya *via* two sequential rounds of WGD (2R). In fish, there was a further teleost-specific WGD (3R) approximately 300 Mya, with several individual lineages having further WGD events, as occurred in the salmonids (4R) approximately 95 Mya and in carp (4R) 5.6–11.3 Mya (46, 71). Many immune genes are retained after a WGD that may have a beneficial role. Thus, a mammalian cytokine gene can have two paralogs in 3R teleosts and up to four paralogs in 4R fish, as seen with *IL1β* (60), *TNFα* (41), and *IL12* (49). The two *IL2* paralogs present in salmonids share only low aa sequence identity, a range similar to 3R paralogs (41, 49). However, our phylogenetic tree and synteny analysis clearly indicate that the salmonid *IL2A* and *IL2B* arose from the 4R salmonid WGD. The two common carp *IL2* paralogs, sharing 51% aa identity, might also have arisen from the 4R WGD event in this lineage (71). By contrast, the two *IL2* paralogs found only in percomorphs, share even lower aa identity (Table S4 in Supplementary Material) but are located at the same genomic site and clearly arose from a local gene duplication event in this lineage. Thus, the expansion of *IL2* in teleosts originated from lineage-specific pathways, local gene duplications, and 4R WGD.

Although little is known about the specific function of each paralog possessed in fish, the high aa sequence divergence of *IL2* paralogs (Table S4 in Supplementary Material) within and between different lineages suggests a functional diversification. The functional diversification, neo-/sub-functionalization, can be demonstrated by changes in temporal and spatial gene expression at the transcript level or by changes of protein function as seen with the rainbow trout paralogs that will be discussed later. The functional diversification can also be reflected by diversification of aa sequence impacting secondary and tertiary structure that may affect ligand–receptor binding and signaling. The disulfide-bonding potential in IL-2 and related molecules from different lineages is also worth noting. Mammalian IL-2 forms a single-disulfide bond to stabilize its structure and is critical for its bioactivity (45). The salmonid IL-2A, and other fish IL-2 including the two carp IL-2, have three predicted disulfide bonds while salmonid IL-2B and percomorph IL-2L have the potential to form two disulfide bonds. Whether more disulfide-bonding plays an important role in stabilizing fish IL-2 isoforms at low physiological temperature in a changing environment is an interesting research area for future studies.

Mammalian IL-2 signals *via* dimeric (IL-2Rβ/γC) or high-affinity trimeric (IL-2Rα/IL-2Rβ/γC) receptors. In the latter, when IL-2Rα binds IL-2 it stabilizes a secondary binding site for presentation to IL-2Rβ. γC is then recruited to the composite surface formed by IL-2/IL-2Rβ (10). In this complex, γC contacts IL-2 helices A and D, IL-2Rβ contacts IL-2 helices A and C, and IL-2Rα interacts mainly with the long AB loop. IL-2Rα forms the largest of the three IL-2/IL-2R interfaces (11). There are marked differences in the AB loop that is encoded by exon 2, between mammalian and fish *IL2* orthologs as seen by comparison of their gene organization and aa sequence (Figures 1 and 2) which reveals the AB loop is smaller in fish. This difference may suggest that the two IL-2 isoforms have evolved binding preferences (affinity) to a specific IL-2 receptor (assuming multiple IL-2 receptors in fish). While *IL2Rβ* and *γC* are present in fish with two copies in salmonids (13), the *bona fide IL2Rα* (CD25) with two Sushi domains has never been discovered in teleosts. However, CD25L or IL-15Rα, with a single Sushi domain, has been characterized and shown to bind IL-15 strongly, and IL-2 to some extent (14). Furthermore, in addition to an *IL15* gene, teleosts possess an *IL15-Like* (*IL15L*) gene that is present in some mammals but pseudogenized in humans and mice (15). Mammalian (cow) IL-15L also binds IL-15Rα. Thus, the fish IL-2, IL-15, and IL-15L may all share three types of IL-2R subunits, CD25L, IL-2Rβ, and γC, with two isoforms each in some fish species. Understanding how this receptor sharing by multiple ligands (e.g., five known receptor subunits, and five ligands, IL-2A, IL-2B, IL-15, IL-15LA and IL-15LB in salmonids) helps regulate the immune response in fish is a fascinating but challenging topic to fish immunologists.

Interestingly, marked differences are present in the two salmonid IL-2 paralogs regarding the size of exon 2 and exon 3. This difference may impact receptor-binding affinity/signaling, leading to functional diversification. It is noteworthy that the two paralogs present in salmonids and percomorphs that arose *via* different evolutionary pathways (4R WGD in salmonids and local gene duplication in percomorphs) share exon 3 size diversification, with a larger exon 3 in salmonid *IL2A* and percomorph *IL2*, and a smaller exon 3 in salmonid *IL2B* and percomorph *IL2L*. This may suggest convergent evolution has occurred.

While the pI of salmonid IL-2A and IL-2 in other teleosts is acidic, that of all salmonid IL-2B is 2.14–3.04 higher. Protein pI is the pH at which the molecule carries no net electrical charge and determines the net charge at a specific environmental pH. The structure, stability, solubility, and function of a protein depend on its net charge and on the ionization state of the individual residues, both of which depend on the pH of the surrounding environment (72). The salmonids studied belong to the Salmoninae and are anadromous fish that evolved in freshwater in the Northern Hemisphere (73). Two isoforms of IL-2 with a different pI may be beneficial in coping with the changing pH of freshwater and ocean environments.

It is noteworthy that the IL-2 paralogs discovered in percomorphs and in 4R WGD teleost species (salmonids and common carp) share low aa sequence identity and appear to be fast-evolving regardless of the mode of duplication. This suggests an inherent potential for sub-/neo-functionalization in such molecules, as demonstrated here for trout IL-2A and IL-2B.

### IL-2 Isoforms Are T Cell Factors

Highest expression of both trout *IL2A* and *IL2B* was seen in thymus, spleen, gills, kidney, and intestine, important tissues/organs in T cell development and function. While their constitutive expression level in PBL was relatively low it could be upregulated by MLR, an alloantigen-mediated T cell activation, by the T cell mitogen PHA, and by signal mimics of T cell activation (PMA and CI stimulation). Previously, *IL2A* was found to be upregulated in PBL by T cell activation with the costimulatory signal CD80/CD86 (22) and *in vivo* in CD4-1 and CD4-2 double-positive T cells after bacterial infection (74). These expression patterns suggest that both IL-2 isoforms are T cell factors secreted by activated T cells.

Interestingly, the two *IL2* paralogs are differentially expressed and modulated. *IL2B* expression is lower constitutively relative to *IL2A* but is more inducible, as seen with stimulation with PHA and PMA + CI. Differential expression and modulation is a common feature of duplicated paralogs as seen with salmonid *IL1β* (60), *TNFα* (41), *IL4/13* (24), *IL-12* (48, 49) and *IL17A/F* (25) genes. In this study, many paralogous genes, e.g., *TNFα, IL-12*, and cytokine/chemokine receptors, are differentially modulated by the two IL-2 isoforms. These differential expression patterns and modulation of paralogs hint at functional diversification.

### Potential Role in Th Cell Development and Adaptive Immunity in Fish

In mammals, when naive CD4+ T cells recognize a foreign antigen-derived peptide presented in the context of MHC class II on APCs, they undergo massive proliferation and differentiation into distinct Th cell subsets such as Th1, Th2, Th17, and induced T-regulatory cells. Each cell subset expresses a unique set of signature cytokines (75). Cytokines produced by these Th subsets play a critical role in immune cell differentiation, effector subset commitment, and in directing the effector response. Although evidence for the existence of mammalian type Th cells in fish is elusive, the major cytokine players are present (5). In this study, both trout IL-2 isoforms could upregulate the expression of signature cytokines for Th1 (*IFNγ1, IFNγ2*, and *TNFα2*) and Th2 (*IL-4/13B1* and *IL4/13B2*) cells, but have no effects on Th17 cytokines (*IL17A/F1A, IL-17A/F2A*, and *IL17A/F3*), and limited effects on Treg cytokines (*TGFβ1* and *IL10*). Furthermore, IL-2 could modulate the expression of receptors for IFNγ and IL4/13 and maintain the expression of Th cell markers (e.g., *CD4-1, CD4-2A*, and *CD4-2B*). Importantly, *IL12P40B2* and *IL12P40C* are induced by both IL-2 isoforms with *IL12P35A1* upregulated by IL-2A and suggests that two isoforms of IL-12 (a driver cytokine for Th1 cell development in mammals) can be produced in response to trout IL-2 that are known to have distinct bioactivities in terms of induction of *IFNγ* and *IL10* expression (71). Overall, these findings indicate that the trout IL-2 isoforms may have an important role in regulating Th1 and Th2 cell development and adaptive immune responses in fish.

### Is There a Role of IL-2 in Treg Cell Development?

Although originally described as a potent T cell growth factor *in vitro*, the main non-redundant role of IL-2 *in vivo* is now known to be the maintenance of peripheral T cell tolerance by promoting the thymic development, peripheral homeostasis, and suppressive function of Treg cells in mammals (76). Treg cells express the signature transcription factor Foxp3, which is critical for their development, lineage commitment, and secretion of IL-10 and TGF-β (75). Both IL-2 isoforms upregulate *FOXP3* and *IL10* expression to a small extent, but have limited effects on *TGFβ1*. This may suggest that the role of IL-2 in Treg cell development is somewhat conserved in fish. However, the mammalian IL-2/Treg paradigm is linked with CD25 expression and hence the high-affinity IL-2R on Treg cells, allowing Treg cells to efficiently compete with effector CD4+ T cells for the available IL-2 (7). Due to the lack of a *bona fide* CD25, a high-affinity IL-2R specific for IL-2 may not exist in fish. Instead, receptors with different affinities may be shared by at least five IL-2/15 cytokines (discussed above) in salmonids. The contribution of each of these IL-2/15 cytokines to Treg cell development in fish remains to be determined.

### Functional Diversification of IL-2A and IL-2B

The aa sequence and exon size diversification of salmonid IL-2 hints at functional diversification. This notion was confirmed by bioactivity analysis of the two recombinant IL-2 isoforms. While in most cases the modulated gene expression by the two IL-2 isoforms was similar (overlapping) when added to cells alone or together, the upregulation of *IL12P35A1* and *CXCR1* expression was found in only IL-2A-treated samples. In several cases (e.g., *IFNγ1, IFNγ2, CXCL11L1, CD8α, CD8β* and *CXCR2*), the response seen was more IL-2B like when the two molecules were added together, with a stronger induction stimulated by IL-2B alone. However, in a few cases (e.g., *IL12P40C* and *IL4Rα2* at 4 h), a stronger response was seen when both IL-2 were present. These bioactivity patterns may suggest that each IL-2 isoform signals independently *via* a pool of receptors expressed in different cells.

It is known that rainbow trout possess one CD25L, and two IL-2Rβ and γC that potentially form four intermediate affinity receptors (IL-2Rβ/γC) and four high-affinity receptors (CD25L/IL-2Rβ/γC). The receptor subunits are differentially expressed and modulated in different cell types (13, 14), and this will determine which functional receptors are available on a cell. It is quite possible that each IL-2 isoform evolved a preference (high affinity) for binding to a specific receptor (discussed above), as part of receptor–ligand co-evolution. IL-2B may have higher affinity receptors on the cells that expressed *IFNγ1, IFNγ2, CXCL11L1, CD8α, CD8β* and *CXCR2*, even though IL-2A can induce a similar response (albeit weaker) in the absence of IL-2B. Similarly, IL-2A may have higher affinity receptors on the cells that expressed *IL12P35A1* and *CXCR1*.

### IL-2 and Host Defense

Both trout IL-2 isoforms induce the expression of Th1 (e.g., *IFNγ* and *TNFα*) and Th2 (e.g., *IL-4/13B*) cytokines in PBL and HK cells (data not shown), that are crucial for host defense against intracellular pathogens and extracellular helminthic parasites in mammals, respectively. They also modulate PBL expression of chemokine receptors important in leukocyte trafficking, and the antimicrobial peptide *cathelicidin-1*, key components of innate immune defense against microorganisms (70). Moreover, they enhance PBL phagocytosis of myeloid cells. Clearly, IL-2 isoforms have an important role in fish defense.

Interestingly, trout IL-2 isoforms induce only *cathelicidin-1* but not *cathelicidin-2* in PBL. This is a similar situation to that found with trout IL-4/13 isoforms in HK cells (24), but in contrast with trout IL-6 that induces *cathelicidin-2* but not *cathelicidin-1* (42). This may relate to the signaling pathways used by different cytokine families in relation to the transcription factors needed for cathelicidin 1 or 2 expression.

### Is IL-2 a T Cell Growth Factor in Fish?

Besides its potent T cell growth factor activity, mammalian IL-2 induces proliferation of NK cells and B cells (7). Trout IL-2 isoforms can promote PBL growth *in vitro* as shown by enhanced BrdU incorporation, although direct evidence on T cell growth was not analyzed in the current study. However, the maintenance of high-level expression of T cell markers (*CD4-1, CD4-2, CD8α*, and *CD8β*) after IL-2 stimulation at late time points, when their expression was decreased in the absence of IL-2, suggests that T cell proliferation could contribute to the enhanced PBL growth seen.

### Conclusion

Two divergent *IL2* paralogs are present in salmonids due to the salmonid 4R WGD. The salmonid *IL2* paralogs differ not only in sequence but also in exon sizes. The IL-2 isoforms encoded have disparate pI values and may have evolved preferential binding to specific IL-2 receptors. Rainbow trout *IL2* paralogs have highest constitutive expression in thymus, spleen, gills, kidney and intestine, important tissues/organs in fish T cell development and function. While their transcript levels are relatively low in PBL, their expression can be upregulated by MLR, by the T cell mitogen PHA, and by signal mimics of T cell activation. Both trout IL-2 isoforms promote PBL proliferation and sustain high-level expression of the T cell markers CD4 and CD8, suggesting that trout IL-2 isoforms are T cell growth factors mainly expressed by activated T cells. The two trout IL-2 isoforms have shared but also distinct bioactivities. IL-2A, but not IL-2B, induces *IL12P35A1* and *CXCR1* expression in PBL. IL-2B has a stronger effect on upregulation of the Th1 cytokine *IFNγ* and sustaining *CD8α* and *CD8β* expression. Both proteins upregulate the expression of key Th1 and Th2 cytokines, cytokine and chemokine receptors, and the antimicrobial peptide *cathelicidin-1*, and enhance phagocytosis of myeloid cells in PBL. Our results suggest that *IL2* paralogs have an important role in regulating Th1 and Th2 cell development and host defense in fish.

## Ethics Statement

All the experiments described comply with the Guidelines of the European Union Council (2010/63/EU) for the use of laboratory animals and were carried out under UK Home Office project license PPL 60/4013, approved by the ethics committee at the University of Aberdeen.

## Author Contributions

TW and CS contributed conceptually, to the design of the study, and wrote the first draft of the manuscript. TW, YH, EW, FL, AW, EZ, ML, and QX performed the experiments. All the authors analyzed the data, contributed to the manuscript revision, read, and approved the submitted version.

## Conflict of Interest Statement

The authors declare that the research was conducted in the absence of any commercial or financial relationships that could be construed as a potential conflict of interest.

## Footnotes

^1^http://blast.ncbi.nlm.nih.gov/Blast.cgi.

^2^http://www.expasy.org/tools.

^3^http://disulfind.dsi.unifi.it.

^4^http://www.compbio.dundee.ac.uk/jpred.

## Abbreviations

CH, chromosome; CI, calcium ionophore; IL, interleukin; MLR, mixed leukocyte reaction; Mya, million year ago; PBL, peripheral blood leukocytes; PHA, phytohemagglutinin; PMA, phorbol 12-myristate 13-acetate; Th1, T helper 1; Th2, T helper 2; Th17, T helper 17; WGD, whole genome duplication.
