# Supplementary material for: Interleukin (IL)-2 Is a Key Regulator of T Helper 1 and T Helper 2 Cytokine Expression in Fish: Functional Characterization of Two Divergent IL2 Paralogs in Salmonids
Source: Front Immunol. 2018 Jul 26;9:1683. doi: 10.3389/fimmu.2018.01683 (PMC6070626; doi:10.3389/fimmu.2018.01683)
Supplement: Supplementary file 1 [file data_sheet_1.PDF]

# Interleukin(IL)-2 is a key regulator of Th1 and Th2 cytokine expression in fish: functional characterisation of two divergent *IL2* paralogues in salmonids

Tiehui Wang, Yehfang Hu, Eakapol Wangkahart, Fuguo Liu, Alex Wang, Eman Zahran,

Kevin Maisey, Min Liu, Qiaoqing Xu, Mónica Imarai, Christopher J Secombes,

**Supplementary Materials**

**Table of contents**

Protein sequences……………………………………………………………………………………………Page 2
Fig. S1. Nucleotide and deduced amino acid sequences of Atlantic salmon *IL2B*………………………………Page 5
Fig. S2. Nucleotide and deduced amino acid sequences of trout *IL2B*…………………………………………Page 6
Fig. S3. Prediction of the Atlantic salmon *IL2A* gene…………………………………………………………Page 7
Fig. S4. Prediction of the coho salmon *IL2A* gene……………………………………………………………Page 8
Fig. S5. Prediction of the coho salmon *IL2B* gene……………………………………………………………Page 9
Fig. S6. Prediction of the chinook salmon *IL2A* gene………………………………………………………Page 10
Fig. S7. Prediction of the chinook salmon *IL2B* gene………………………………………………………Page 11
Fig. S8. Prediction of the Arctic char *IL2A* gene……………………………………………………………Page 12
Fig. S9. Prediction of the Arctic char *IL2B* gene……………………………………………………………Page 13
Fig. S10. Amino acid sequence alignment of salmonid IL-2 (A) and predicted potential intra-molecular disulfide bonds (B) ……………………………………………Page 14
Fig. S11. Mixed leucocyte reaction induced *IL2* expression in rainbow trout head kidney (HK) cells. …………Page 15
Fig. S12. SDS-PAGE analysis of rainbow trout IL-2A and IL-2B recombinant proteins. ………………………Page 16
Fig. S13. Modulation the expression of *IFNγ1* (A), *IFNγ2* (B), *TNFα1* (C), *TNFα2* (D) by IL-2 isoforms in HK cells……………………………………………Page 17
Fig. S14. Modulation of other Th1 pathway gene expression by IL-2 isoforms in PBL…………………………Page 18
Fig. S15. Modulation of Th17 pathway gene expression by IL-2 isoforms in PBL………………………………Page 19
Fig. S16. Modulation of proinflammatory cytokine and hepcidin gene expression by IL-2 isoforms in PBL…...Page 20
Table S1 Primers used for cloning, expression analysis and recombinant protein production of salmonid IL2A and IL2B genes ……………………………………………Page 21
Table S2. Summary of sequence analysis of salmonid IL-2……………………………………………………Page 22
Table S3. Primers used for real-time RT-PCR analysis of gene expression……………………………………Page 23
Table S4. Characteristics (top) and comparison of amino acid identity / similarity (bottom) of IL-2 like molecules from salmonids, percomorphs, common carp and mammals…………………………Page 25

**Protein sequences**

Amino acid sequences used for multiple alignments and phylogenetic tree analysis are below:

# Interleukin-2

```
>Atlantic salmon IL-2A, predicted from AGKD04000200
MDRLYRISFLTLFLAGCLQGNPIFRLKVGINYLEENITCPDSVFYTPTDVEDSCIVAALACSMKELSIVK
AECLDNVTNWENMQHHINRTITTLQMMIDKDNSTTDTSECICEDTRLEKSFKDFLQNILHLAEAHAVKRG
>Trout IL-2A, NP_001157537
MDRRYRISFLTLFLTGCLQGNPIPRLLAGIDYLEENITCPDSVFYTPTDVEDSCIVAALACSIKELDTVK
VECLDKAVHLESMQHHISMTATALQKTIDKENSTTDTSECICEDKRLEKSFKDFIQNIRHLTQAHAAKRL
SS
>Coho IL-2A, predicted from MPKV01002376
MDCLYRISFLTLFLAGCLQGNPISIDYLEESITCPDSVFYTPTDVEDSCIVAALACSIKELDTVKVECLD
NAIYLESMQYHISMTATDLQKTIDKENSTTDTSECICEDKRLEKSFKDFIQNLRHLTQAHAAKHK
>Chinook IL-2A, predicted from PIPH01030521
MDCLYRISFLTLFLAGCLQGNPISILQVEIDYLEESITCPDSVFYTPTDVEDSCIVAALACSIKELDTVK
VECLDNAIHLESMPHHISMTATALQNTIDKENSTTDTTECICEDKRLEKSFKDFIQNIRHLTQAHAAKRR
>Char IL-2A, predicted from PPUY01000011
MDRLYRISFLTLFLAGGLQGNPINRLEYLEKNAIVGIEYLEKEITCPDSVFYTPTDVKGSCIVAALACSI
KELYTVKEECLDNTTKLVNMQHQINMTAKDLQKMIDEENSTTDTSECICEDTRFEKSFKDFIQNIRHLTG
AYAEKRG
>Atlantic salmon IL-2B,
MEHIFRSAFLMLFLSVGLQGHPANNLERIQIGIQHLQKNIKCPEGTVFYAPSPADVEKSIAGALACSIQQ
LNPLDNTNLQHHINKTLKVLQATFVDDIRTDSSECSRENPLFKKSCKEFLENMTSLGQALSVKSSK
>Trout IL-2B,
MENIFRIAFLMLFLSVGLQGSPATNYLKIAKGIERLQINITCPEEGTFFYAPSPADVEKSIAGALACSIQ
QLNHLNDNRNLQHHINRILTVLQKTSDDIRTDSSECSRENPLSKKSCKEFMENMISLAKALSAQRSP
>Coho IL-2B, predicted from MPKV01002536
MENIFRIAFLMLFLSVGLQGSPATNFLKIAKGIERLQINITCPEEGTFFYAPSPADVEKSIAGALACSIQ
QLSHLNDNTNLQHHINRILTVLQKTSDDIRTDSSECSRENPLSKKSCKEFMENMISLAKALSAQRYPW
>Chinook IL-2, predicted from PIPH01048965
MENIFRIAFLMLFLSVGLQGSPANNFLKIAKGIERLQINITCPEEGTFFYAPSPADVEKSIAGALACSIQ
QLNHLNDNRNLQHHINRILTVLQKTSDDIRTDSSECSRENPLSKKSCKEFMENMISLAKALSAQRSP
>Char IL-2B, predicted from PPUY01016320
MENIFRIAFLMLFLSVGLQGLPTKNFMTVQHGIKHLQKQITCPEGTVFYAPSPADVEKSIAGALACSIQQ
LNHLNNTPNLQHHINMTLAVLQATFVDDTTTDSSECSRENPLSKKSCKEFMKNMTSLAEALFVKYSQS
>Fugu IL-2, NP_001033083
MENFIRINVWLGILCLCFPANPFPLHLEDSNIDVIREDVKCEPDSKFYTPANVRDDHHCIIVALECVAAE
LKTVRRECEDPEDVIGVAEEFLTHTIQKLKNGVKIEKSNSTECSTCESWPEKPLTNFLDATESLLQQVQS
GAIPSAEGS
>Tetraodon IL-2, ABS44960
METFNRIYFGMVIVCVCLPANSNPMPLLDDSDIGDMKKNVICEQDSKFYTPTNIKPECLTAALQCFKDEL
QTVKHECKDPQNYINRTKGFLEHVISTMKNEEVNSNACSCESYSEEPFPEFLNAMETLVQRFNSKARQNQ
QR
>Stickleback IL-2, ABS44953
MFFFIQMAYWILLSDCLLARSFPLSDFRAITQSHVECRSDSRFYAPSDVTEACITTALDCVMRELNGTVK
EECDDSEQDILDAVESLNHVINRRTTAGHARTDSNECTCERWPLASYAVFKKNTLNLLQMTNTMG
>Fugu IL-2L, NP_001129615
MSWITLALLMVPLIGHLRAAPLATPQRLSMEALGFELLDEITCEKEKDLNLTSPTNVEDKCYNAALGHYI
KEFQRTIGNCTDAGDIVTTVEELERIYSETQTACTLTMKTHATFIGFVKATEAFAQQYNDS
>Tetraodon IL-2L, ABS44961
MTWIAIALWLVPLIGQVQVRPVSNDTEPLGPLNMDDLGLQFLDKRLCGDGMNFTSPTNVMTKCHGAALGL
FIQEFKKVYTHCGENSPVKQTIEVLERAHNKTQAEECTLTMTDHKNFEDLIDAMKHFAWRINSAY
>Stickleback IL-2L, ABS44954
```

MEHSLRTALWVFCLFGFLQATPPCYGQGDLGFCFLQQHVKCVNVTFTYPINVQAKCSRDALQVFVQGLNN
ATTDCQDDQEIIPDTLESLAWKFPTTDSTNCKLQTKESQFEDFVKDLERLVQLINASGDK
>Common carp IL-2A, AKN90080
MFALHWICALTLALVSCLSSQPVKRDANEQLYHVSELKAAIENFECPDDMSLYSPVNIRKECMSSALDCT
LEELKVLKSECDIDEADIETVDNVSDLLSQTNWNIASSSSPNCSCEMYDPVDVKQFIDNIKPQVQQLNSM
KVTKATANL
>Common carp IL-2B, AKN90081
MFALHRICALTLALVICLNAQPVKRDADELLEHISTLKTFIGTAKCPDHIHLHSPFNIKKDCMSSARDCT
IKMLYDLKSKCNITKNSTTDEAINNVILELGTEETNPTSSSPDCKCEMYNKTGVDQFLKNMEVQAEQLNS
MQ
>Human IL-2, P60568
MYRMQLLSCIALSLALVTNSAPTSSSTKKTQLQLEHLLLDLQMILNGINNYKNPKLTRMLTFKFYMPKKA
TELKHLQCLEEELKPLEEVLNLAQSKNFHLRPRDLISNINVIVLELKGSETTFMCEYADETATIVEFLNR
WITFCQSIISTLT
>Cow IL-2, P05016
MYKIQLLSCIALTLALVANGAPTSSSTGNTMKEVKSLLLDLQLLLEKVKNPENLKLSRMHTFDFYVPKVN
ATELKHLKCLLEELKLLEEVLNLAPSKNLNPREIKDSMDNIKRIVLELQGSETRFTCEYDDATVNAVEFL
NKWITFCQSIYSTMT
>Rat IL-2, P17108
MYSMQLASCVALTLVLLVNSAPTSSPAKETQQHLEQLLLDLQVLLRGIDNYKNLKLPMMLTFKFYLPKQA
TELKHLQCLENELGALQRVLDLTQSKSFHLEDAGNFISNIRVTVVKLKGSENKFECQFDDEPATVVEFLR
RWIAICQSIISTMTQ

# Interleukin-15

>Human IL-15, P40933
MRISKPHLRSISIQCYLCLLLNSHFLTEAGIHVFILGCFSAGLPKTEANWVNVISDLKKIEDLIQSMHID
ATLYTESDVHPSCKVTAMKCFLLELQVISLESGDASIHDTVENLIILANNSLSSNGNVTESGCKECEELE
EKNIKEFLQSFVHIVQMFINTS
>Mouse IL-15, P48346
MKILKPYMRNTSISCYLCFLLNSHFLTEAGIHVFILGCVSVGLPKTEANWIDVRYDLEKIESLIQSIHID
TTLYTDSDFHPSCKVTAMNCFLLELQVILHEYSNMTLNETVRNVLYLANSTLSSNKNVAESGCKECEELE
EKTFTEFLQSFIRIVQMFINTS
>Cow IL-15, Q28028
MRILKPYLRSTSIQCYLCLLLNSHFLTEAGIHVFILGCISASLPKTEANWQYVINDLKTIEHLIQSIHMD
ATLYTESDAHPNCKVTAMQCFLLELRVILHESKNATIYEIIENLTMLANSNLSSIENKTELGCKECEELE
EKSIKEFLKSFVHIVQMFINTS
>Trout IL-15, CAD88594
MTGFLTVLLFCIRLLERRTKKSVRWICLFWGFHYYPHQRLNIELWNCFIILSCLSATAHLPIAGAAETHG
MTIDDVKELQSELKNLKSTIEKSDACLYAPTNDDIYNDHCIFKFMHCYLLELEVVLFEDMSVTDNYHDEI
KTSIYHRKKHLEEHERQYNSSRCSPCEAQRVANSTIFLYNLERLLERIGQTVS
>Atlantic salmon IL-15, NP_001265994
MTGFLTVLLFCIRLLERRTKKRVRWICLFWGFHYYQHLCLNIELWNCFLILSCLSATAHLPIAGAAETHG
MTIDDVRELQSELLNLKSTIEKSDACLYAPTNDDIYNDNCIFKFMHCYLLELEVVLIEDMQVTDNYHDEI
KTSIYHRKKMLEEHERQYNSSRCSPCEAQRVANSTIFLYNLERLLERIGTTVS
>Carp IL-15, KTG32966
MTHFKDSYIRRTCELHAAVVVGLPTILLSMILMTLFLVFIVGIWNKPAKLKSKRTGRCACNPWCFESHME
CLLNSEVWNSILILSCLSALLPVAEGNEMQALKDLQNALQDKETKLLFQRSNVRLYTPNTEDIKNCTFKF
FDCFLMEMNVLLHDEDPTNENQLLIKTTLEDYTKIFTVMNRKAADFKCSERYPCELQELTNSREFFDRMT
NFLEKQQVLCRQPSTITCD
>Stickleback IL-15, NP_001254613
MTDFMTVFPEILVKASQKGFQLRSTCHLFQESHKPQVWLCFLVLSLLSTSTCAAPGAAKLAHLQSCLGTE
YLKKAIEKSDAMLYAPSANEPKENCKMMSLKCYILELRMVIVEEVGSENPKTHCVMDFNERLPDLDNGAS
AYPVDCPPCEAYSLKNITVFMERLNSLLQELNSMQT
>Fugu IL-15, NP_001028220
MRDFMMVQPTCLGEQRATGVHFQSSCHLCREILKAQVWFHLFVLCFLSPYTCAASVPETRGVQICVKALR
PAIENSDAMLYAPSANSVKKQCKNMSLRCYMLELIMVINEEEIMDNNANCISDFNEILPTDNSVGCPPCE

EYAIRNITIFLGRLQSLLEELNVIQNT
>Tetraodon IL-15, AAZ23017
MSGFMTATAQPTCARGHRSRGVSFHSTCCLCREFKTWFPFFLLCFLSLYTCHAAVVETSEVKTCITAIQP
AIEKSDAMLYTPSANYVGWSSQGKKNCKNKSLKCYMLELLMVIDEEAETGMYPNCINDFNARLLPDNSVG
CPPCEVYPQNNITIFLRELQNLLEEINVINRT

# Interleukin-21

>Human IL-21, Q9HBE4
MERIVICLMVIFLGTLVHKSSSQGQDRHMIRMRQLIDIVDQLKNYVNDLVPEFLPAPEDVETNCEWSAFS
CFQKAQLKSANTGNNERIINVSIKKLKRKPPSTNAGRRQKHRLTCPSCDSYEKKPPKEFLERFKSLLQKM
IHQHLSSRTHGSEDS
>Mouse IL-21, Q9ES17
MERTLVCLVVIFLGTVAHKSSPQGPDRLLIRLRHLIDIVEQLKIYENDLDPELLSAPQDVKGHCEHAAFA
CFQKAKLKPSNPGNNKTFIIDLVAQLRRRLPARRGGKKQKHIAKCPSCDSYEKRTPKEFLERLKWLLQKM
IHQHLS
>Cow IL-21, Q76LU5
MERIVICLMVIFSGTVAHKSSSQGQDRLFIRLRQLIDIVDQLKNYVNDLDPEFLPAPEDVKRHCERSAFS
CFQKVQLKSANNGDNEKIINILTKQLKRKLPATNTGRRQKHEVTCPSCDSYEKKPPKEYLERLKSLIQKM
IHQHLS
>Atlantic salmon IL-21, ACJ02096
MKLLVCCLLAITCCVLANADNVERTMKLTEVLKELRQLNKSVEHNGVMLNTPTMDIEECCFLSTLECFQK
MVPRLKTKQKKLQCKVSRNLNPLTFTGVDSCSREEREKKVCQGCDSYPMKDSREFVQQLESLLQKVAGYY
E
>Trout IL-21, AJA40852
MKLLVCCLLAITCCVLANVDKVERTMKLTEVLKELRQLNKSVAHNGMMLNTPTLDIEECCFLSALECFRK
MVPSLNAKQKKLQRKVIKNLSPLTFRGVDSCSREERENKVCQGCDSYPMKDSREFVKQLESLLQKAMNRL
V
>Carp IL-21, XP_018943056
MKASVCFVFAVVCWIAAQAEVSPMILTLSKVMNELNNISKEMNKSTTLKSPTINDLEDCCIKSALDCFRS
KVFHLSVSDRKLKQSQRIISHELHKSIIVNSVSNCKPEETQKAQCKSCDSYKVVDSQTFVQNFQTLLQKI
YSSQV
>Fugu IL-21, NP_001033082
MKPLVLCLFAVCCWCVADASADVSDDKVKQQRKLEEILRELGMVKRRLQNSKKMLSTPSENIGDCCCLSA
LKCFRENFKEIFSLTDLPQKKLYRSLTNTLTEKGLDFCDSKNSTCQDCHSHPEEKAEKFFDRLNSLIQKV
HLKSSICHLMSSLMF
>Tetraodon IL-21, ABB05043
MKQLVFCLFAVCCWWLADASSAECSERKLEEVRRELEGVNNTLQNRELLLTTPPKNIEEGCCLSALRCFR
DSIQENIKSTVRLQRRLYKSLNNSHTAACLNFCHSENATCQTCNSHPQEKVGEFFSRLDSFIQKAISKLR
SSAAN
>Stickleback IL-21, NP_001254615
MKLVVFCFLAAWCGSLVGASTSRPTTRPPMFRRKLEEVLGHLHRVKESQQHNEKTLNIPPQSFKDRCCVS
TLKYFLENLEKQFNASQDKLYRSLKQRKTERALCPSGDIQANCQTYNSHPGTVQVFFERLQSFIEEGITR
LSMK

```
1                                                                M   E   H   I   F   R   S   A   F   L   M   L   F
1   CCTCCCTTCGTGTACATCTCACAGGACACTGCCTACAAGACCTTCAGATTATCATGGAACACATTTTCAGGAGTGCCTTTTTGATGCTTTTT

 14   L   S   V   G   L   Q   G   H   P   A   N   N   L   E   R   I   Q   I   G   I   Q   H   L   Q   K   N   I   K   C   P
 93   CTCTCAGTTGGTCTTCAAGGACATCCGGCTAACAATTTAGAGAGAATTCAAATTGGAATACAACATCTACAAAAGAATATTAAATGTCCA

 44   E   G   T   V   F   Y   A   P   S   P   A   D   V   E   K   S   I   A   G   A   L   A   C   S   I   Q   Q   L   N   P
183   GAAGGTACAGTCTTCTATGCTCCGTCTCCAGCGGATGTAGAGAAAAGCATTGCTGGAGCACTGGCCTGTTCTATTCAACAACTGAACCCC

 74   L   D   N   T   N   L   Q   H   H   I   N   K   T   L   K   V   L   Q   A   T   F   V   D   D   I   R   T   D   S   S
273   CTCGATAACACCAATCTGCAGCATCATATCAACAAGACTTTGAAGGTCCTGCAAGCGACATTTGTCGATGACATCAGAACGGACTCGTCA

104   E   C   S   R   E   N   P   L   F   K   K   S   C   K   E   F   L   E   N   M   T   S   L   G   Q   A   L   S   V   K
363   GAATGCAGCCGTGAGAACCCGCTGTTCAAAAAGTCTTGCAAGGAGTTCCTGGAGAATATGACCAGTTTAGGTCAAGCTCTATCTGTCAAA

134   S   S   K   -
453   TCGTCCAAATGATAGATCCTATTTTATCTCCCAGTTGCATGGGGGGAGGATGGCGTCACAGGAAGTAGATTGTCATTGCACTGATACTAT

543   ATATTATCTCGAACAATTTGTGTAACATTGTGTGACACCAGCTTTTAAAATATATATATTTTATGTCTTACTGGATGTACTTACAAACCT

633   GGGATGAAAGCCCCACATAATGAAGGAAGGGGCTACTGTAGATTCTGTTTAATGATGTGATATCCATATGCTGTGCCTGAACTGCTCTGA

723   ATGCAAGTGTTTGAGTTCTCAATGTCTGTTAACTATAATGTATTTAAGCTTCTAAAAAGCTCTAATTAAGTTATTTATTTATGTATTTAT

813   TACATATTTATTATATTTTGATTTCTACACATGGACTGTTCCTATAACCTTGATAATAAATTTGCAATTGAAAGGAAAAAAAAAAAAAAA

903   AAAAAAAA
```

**Fig. S1. Nucleotide and deduced amino acid sequences of Atlantic salmon *IL2B*.** The cDNA sequence was obtained by 3'- RACE. The start and stop codons for translation are highlighted in red. Putative mRNA instability motifs (ATTTA) and the potential polyadenylation signal in the 3'-UTR are in bold and boxed. Intron positions are indicated by arrows. A predicted signal peptide is highlighted in green. Potential N-glycosylation sites are in purple.

```
  1                                                                                 M  E  N  I  F  R  I  A  F
  1 ACAGACTCCAGACCTCCCTTCGTATACACCTCACAGGACTCTGCCTACAAGACCTTCAGATTATCATGGAAAACATATTCAGGATTGCCTTT
            F1
 10   L  M  L  F  L  S  V  G  L  Q  G  S  P  A  T  N  Y  L  K  I  A  K  G  I  E  R  L  Q  I  N
 93  TTGATGCTTTTTCTCTCAGTTGGTCTTCAAGGAAGCCCGGCTACCAATTACTTGAAAATTGCAAAAGGAATCGAACGACTACAAATAAAT

 40   I  T  C |P  E  E  G  T  F  F  Y  A  P  S  P  A  D  V  E |K  S  I  A  G  A  L  A  C  S  I
183  ATTACATGT|CCAGAAGAAGGTACATTCTTCTATGCTCCGTCTCCAGCGGATGTAGAG|AAAAGCATTGCTGGAGCACTGGCCTGTTCTATT
              ↓                                                ↓
 70   Q  Q  L  N  H  L  N  D  N  R  N  L  Q  H  H  I  N  R  I  L  T  V  L  Q  K  T  S  D  D  I
273  CAACAACTGAACCACCTCAATGACAACAGAAATCTGCAGCATCATATCAACAGGATTTTGACGGTCCTGCAAAAGACATCTGATGATATC

100  |R  T  D  S  S  E  C  S  R  E  N  P  L  S  K  K  S  C  K  E  F  M  E  N  M  I  S  L  A  K
363  |AGAACGGACTCGTCAGAATGCAGCCGTGAGAACCCGCTGTCCAAAAAGTCTTGCAAGGAGTTCATGGAGAATATGATCAGTTTAGCTAAA
     ↓
130   A  L  S  A  Q  R  S  P  -
453  GCTCTATCTGCCCAACGGTCCCCATGATAAAATAGGTTCTAGCTCCCAGTTACATGGGGGGAGAATGGGGTCACAGGAAGTAGATTGTCA

543  TTGCACTGATACTATATATTATCTCTAACAATTTGTGTAACATTGTTTAACACCAGCTTTTAAAATATATATATATATATTTATATGTCT

633  AACTGGATGTCCTTAACCTGGTATGAAAGCCCTACATAATGAAGGAAGGGGCTACTGTAGGTTCTGTTTAATGATGTGATATCCATATGC

723  TGTGCCTGAACTGCtctgaatgcaagtgtttgagttctcaatgtctgttaactataatgtatttaagcttctaaaaagctctaattaagt
         R1
813  tatttatttatgtatttattacatatttattatattttgatttctacacacggactgttcctataaccttgataataaatttgcaattaa
```

**Fig. S2. Nucleotide and deduced amino acid sequences of trout *IL2B*.** The cDNA sequence (capital letters) was obtained by PCR using primers F1 and R1 (underlined). The DNA sequence in small letters is derived from WGS contigs (acc. No. MSJN01001294/CCAF010069625). The start and stop codons for translation are highlighted in red. Putative mRNA instability motifs (ATTTA) and the polyadenylation signal in the 3'-UTR are in bold and boxed. Intron positions are indicated by arrows. A predicted signal peptide is highlighted in green. A potential *N*-glycosylation site is in purple.

```
Exon 1
839582  AAACATTTACAGACC TCCGCTTGTGTACAC CTTACAGGACACTAC CTACAAGCCCTTCAG ATTATCATGGACCGT
                                                                              M  D  R
839505  CTTTACAGGATTTCC TTTTTGACGCTTTTT CTCGCCGGTTGTCTA CAAGGAAACCCAATT TTCAGACTCAAAGTT
         L  Y  R  I  S   F  L  T  L  F   L  A  G  C  L   Q  G  N  P  I   F  R  L  K  V
839440  GGAATCAACTATCTA GAAGAAAACATTACA TGTgtaag
         G  I  N  Y  L   E  E  N  I  T   C

Exon 2
839310  tgtagCCAGATTCAGTC TTCTATACTCCAACT GATGTAGAGgtaag
              D  S  V   F  Y  T  P  T   D  V  E  V

Exon 3
838934  tgcagGATAGTTGCATT GTTGCAGCATTGGCC TGTTCCATGAAGGAA CTGTCCATTGTGAAA GCAGAATGCCTCGAT
              D  S  C  I   V  A  A  L  A   C  S  M  K  E   L  S  I  V  K   A  E  C  L  D
838857    AATGTGACCAATTGG GAAAATATGCAACAC CACATCAACAGGACT ATCACAACCCTACAA ATGATGATTGATAAG
           N  V  T  N  W   E  N  M  Q  H   H  I  N  R  T   I  T  T  L  Q   M  M  I  D  K
838782    GACAACgtaag
           D  N

Exon 4
838645  ctcagAGCACAACGGAC ACTTCAGAATGCATC TGTGAAGACACGCGG TTGGAAAAGTCTTTC AAGGACTTCCTTCAG
              S  T  T  D   T  S  E  C  I   C  E  D  T  R   L  E  K  S  F   K  D  F  L  Q
838568    AACATATTACATTTA GCTGAAGCTCATGCT GTAAAGCGTGGATAA GTCCATACATGCTGG CAATAGAGTGATTGT
           N  I  L  H  L   A  E  A  H  A   V  K  R  G  *
838493    TGTAATATATGGAAC ATTACGTAATTGGGA CAAAATACATGCCAT ACCAACATGTACAGT AGTACCATGTTCCAG
838418    TGATAAACAAGCTCT AGTAGCTTCCAGTTG CTGAACAGTTATATG GGAGAAGATGGGGAG GGGGGTTACAGGAAG
838343    TTTGTCATTGCACCG ATACTCTAATGTGTA TAATCTATCATCTCT AACAATTAGAGGCCC ATTTTTTGGTGTGAA
838268    AACCAGTTCATTTCA TGTATGTCTCACTGG ATGTCCTGAACCAGA GATAGCAAGCCCTCC ATAATGAAGGAAGGT
838193    GCTACGGACGTTATT AATTATGTTTTGTTT CATTATGTGATTTCC ATATACTGTGCCTGA ATGCCAGTGTTTCAG
838118    AGTTCTCAATGTCTG TTTCAATTAGAATGC ATTTGAAATAAGCTC CTAAAAAGCTCTAAT TAAGTTATTTATTTA
838043    TGTATTTATTACGTA TTTATTCTATTTTTA TTTCTGCACATGGAC TGTTCCGAAACCCTG ATAATAAAGTTGAAT
837968    TTAAAAGTA
```

**Fig. S3. Prediction of the Atlantic salmon *IL2A* gene.** The Atlantic salmon *IL2A* was predicted on WGS contig AGKD04000200 using the rainbow trout *IL2A* gene as bait. The gt/ag motifs at the intron boundaries are boxed. The start and stop codons for translation are highlighted in red. Putative mRNA instability motifs (ATTTA) and the polyadenylation signal in the 3'-UTR are in bold and boxed. A predicted signal peptide is highlighted in green. Potential *N*-glycosylation sites are in purple.

**Exon 1**
```
3625856   AAACATTTACAGATC TCCCCTTGTGTACAC CTTACAGGACACTAT CTACAAACCCTTCAG ATTATCATGGACTGT
                                                                                M  D  C
3625931   CTTTACAGGATTTCC TTTTTGACGCTTTTT CTCGCCGGTTGTCTA CAAGGAAACCCAATT TCCATCGATTATCTA
           L  Y  R  I  S   F  L  T  L  F   L  A  G  C  L   Q  G  N  P  I   S  I  D  Y  L
2626006   GAAGAAAGTATTACA TGTgtaag
           E  E  S  I  T   C
```

**Exon 2**
```
3626115 tgtagCCAGATTCAGTC TTCTATACTCCAACT GATGTAGAGgtaag
              P  D  S  V   F  Y  T  P  T   D  V  E
```

**Exon 3**
```
3626496 tgcagGATAGTTGCATT GTTGCAGCATTGGCC TGTTCCATTAAGGAA CTGGACACTGTGAAA GTAGAATGCCTCGAT
              D  S  C  I   V  A  A  L  A   C  S  I  K  E   L  D  T  V  K   V  E  C  L  D
3626571    AACGCGATCTATCTG GAAAGTATGCAATAC CACATCAGCATGACT GCCACGGACCTACAA AAGACGATTGATAAG
            N  A  I  Y  L   E  S  M  Q  Y   H  I  S  M  T   A  T  D  L  Q   K  T  I  D  K
3626646    GAGAACgtaag
            E  N
```

**Exon 4**
```
3626774 ctcagAGCACAACGGAC ACTTCAGAATGCATC TGTGAAGACAAGCGG TTGGAAAAGTCTTTC AAGGACTTCATTCAG
              S  T  T  D   T  S  E  C  I   C  E  D  K  R   L  E  K  S  F   K  D  F  I  Q
3626879    AACCTAAGACATTTA ACTCAAGCTCATGCT GCAAAGCATAAATAA GTCCATAAATGCTGG CAATAGAGTGACTGT
            N  L  R  H  L   T  Q  A  H  A   A  K  H  K  *
3626954    TGTAATATATGGAAC ATTACGTAATTGGGA CAAAATACATGATGA AGTTTGTCATTGCAC TGATACTCTAATGTG
3627029    TATAATCTATCATCT CTAACAATTAGAGGC CCATTATTTGGTGTG AAAACCAGTTAATTT CATTGGTCATGTATG
3627104    TCTCACTGAATGTCC TTAACCGGAGATAGC AAGCCCTCCATAATG AAAGAAGGTGCTAGG GATGTTATTAATTAT
3627179    GTTTTGTTTCATTAT GTGATTTCCATATAC TGTGCCTGAATGCCA GTGTTTCAGAGTTCT CAATGTCTGTTTCAA
3627254    TTAGAATGCATTTGA AATAAGCTCCTAAAA AGCTCTAATTAAGTT ATTTATTTATGTATT TATTACGTATTTATT
3627239    ATATTTTCATTTCTG CACATGGACTGTTCC GAAACCGTGATAATA AAGTTGCATTTAAAA GTA
```

**Fig. S4. Prediction of the coho salmon *IL2A* gene.** The coho salmon IL2A was predicted on WGS contig MPKV01002376 using the Atlantic salmon and rainbow trout IL2A gene as bait. The gt/ag motifs at the intron boundaries are boxed. The start and stop codons for translation are highlighted in red. Putative mRNA instability motifs (ATTTA) and the polyadenylation signal in the 3'-UTR are in bold and boxed. A predicted signal peptide is highlighted in green. A potential *N*-glycosylation site is in purple.

```
Exon 1
109340 CCTCCCTTCGTATACAC CTCACAGGACTCTGC CTACAAGACCTTCAG ATTATCATGGAAAAC ATATTCAGGATTGCC
                                                              M  E  N   I  F  R  I  A
109415   TTTTTGATGCTTTTT CTCTCAGTTGGTCTT CAAGGAAGTCCGGCT ACCAATTTCTTGAAA ATTGCAAAAGGAATC
          F  L  M  L  F   L  S  V  G  L   Q  G  S  P  A   T  N  F  L  K   I  A  K  G  I
109490   GAACGACTACAAATA AATATTACATGTgtaag
          E  R  L  Q  I   N  I  T  C
Exon 2
109606   tgtagCCAGAAGAAGGT ACATTCTTCTATGCT CCGTCTCCAGCGGAT GTAGAGgtaag
                P  E  E  G   T  F  F  Y  A   P  S  P  A  D   V  E

Exon 3
109993 tgcagAAAAGCATTGCT GGAGCACTGGCCTGT TCTATTCAACAACTG AGCCACCTCAATGAC AACACAAATCTGCAG
            K  S  I  A   G  A  L  A  C   S  I  Q  Q  L   S  H  L  N  D   N  T  N  L  Q
110068   CATCATATCAACAGG ATTTTGACGGTCCTA CAAAAGACATCTGAT GATATCgtaag
          H  H  I  N  R   I  L  T  V  L   Q  K  T  S  D   D  I

Exon 4
110258 ctcagAGAACGGACTCG TCAGAATGCAGCCGT GAGAACCCGCTGTCC AAAAAGTCTTGCAAG GAGTTCATGGAGAAT
            R  T  D  S   S  E  C  S  R   E  N  P  L  S   K  K  S  C  K   E  F  M  E  N
110333   ATGATCAGTTTAGCT AAAGCTCTATCTGCC CAACGGTACCCATGG TAAAATAGGTTCTAG CTCCCAGTTGCATGG
          M  I  S  L  A   K  A  L  S  A   Q  R  Y  P  W   *
110408   GGGGAGGATGGGGTC ACAGGAAGTAGATTG TCATTGCACTGATAC CATATATTATCTCTA ACAATTTGTGTAACA
110483   TTGTGTAACACCAGC TTTTAAAATATATAT ATATATTTATATGTC TAACTGGATGTCCTT AACCTGGTATGAAAG
110558   CCCTACATAATGAAG GAAGGGGCTACTGTA GGTTCTGTTTAATGA TGTGATATCCATATA CTGTGCCTTGAACTG
110633   CTCTGAATGCAAGTG TTTGAGTTCTCAATG TATGTTAACTATAAT GTATTTAAGCTTCTA AAAAGCTCTAATTAA
110708   GTTATTTATTTATGT ATTTATTACATATTT ATTATATTTAGATTT TTACACATGGACTGT TCCTATAACCTTGAT
110783   AATACATTTGCAATT AAAAG
```

**Fig. S5. Prediction of the coho salmon *IL2B* gene.** The coho salmon *IL2B* was predicted on WGS contig MPKV01002536 using the Atlantic salmon and rainbow trout *IL2B* gene as bait. The gt/ag motifs at the intron boundaries are boxed. The start and stop codons for translation are highlighted in red. Putative mRNA instability motifs (ATTTA) and a canonical polyadenylation signal (ATTAAA) in the 3'-UTR are in bold and boxed. A predicted signal peptide is highlighted in green. A potential *N*-glycosylation site is in purple.

**Exon 1**

```
7422   AAACATTTACAGATC TCCCCTTGTGTACAC CTTACAGGACACTAT CTACAAACCCTTCAG ATTATCATGGACTGT
                                                                              M  D  C
7497   CTTTACAGGATTTCC TTTTTGACGCTTTTT CTCGCCGGTTGTCTA CAAGGAAACCCAATT TCTATACTCCAAGTT
        L  Y  R  I  S   F  L  T  L  F   L  A  G  C  L   Q  G  N  P  I   S  I  L  Q  V
7572   GAAATCGATTATCTA GAAGAAAGTATTACA TGTgtaag
        E  I  D  Y  L   E  E  S  I  T   C
```

**Exon 2**

```
7696   tgtagCCAGATTCAGTC TTCTATACTCCAACT GATGTAGAGgtaag
             P  D  S  V   F  Y  T  P  T   D  V  E
```

**Exon 3**

```
8075 tgcagGATAGTTGCATT GTTGCAGCATTGGCC TGTTCCATTAAGGAA CTGGACACTGTGAAA GTAGAATGCCTCGAT
           D  S  C  I   V  A  A  L  A   C  S  I  K  E   L  D  T  V  K   V  E  C  L  D
8150   AACGCGATCCATCTG GAAAGTATGCCACAC CACATCAGCATGACT GCCACGGCCCTACAA AACACGATTGATAAG
        N  A  I  H  L   E  S  M  P  H   H  I  S  M  T   A  T  A  L  Q   N  T  I  D  K
8225   GAGAACgtaag
        E  N
```

**Exon 4**

```
8367 ctcagAGCACAACGGAC ACTACAGAATGCATC TGTGAAGACAAGCGG TTGGAAAAGTCTTTC AAGGACTTCATTCAG
           S  T  T  D   T  T  E  C  I   C  E  D  K  R   L  E  K  S  F   K  D  F  I  Q
8442   AACATAAGACATTTA ACTCAAGCTCATGCT GCAAAGCGCCGATAA GTCCATAAATGCTGG CAATAGAGTGACTGT
        N  I  R  H  L   T  Q  A  H  A   A  K  R  R   *
8517   TGTAATATATGGAAC ATTACGTAATTGGGA CAAAATACATGCCAT ACCAACATGTACAGT AGTACCATGTCTATC
8592   TGTTCCAGTGATAAA GAAGCTCTAGTAGCT TCCACTTGCTGAACA GTTATATGGGGGGAA ATGGGGGGGGTTTAC
8667   AGGAAGTTTGTCATT GCACTGATACTCTAA TGTGTATAATCTATC ATCTCTAACAATTAG AGGCCCATTATTTGG
8742   TGTGAAAACCAGTTA ATTTCGTTGGTCATG TATGTCTCACTGTAT GTCCTTAACCGGAGA TAGCAAGCCCTCCAT
8817   AATGAAGGAAGGTGC TAGGGATGTTATTAA TTATGTTTTGTTTCA TTATGTGATTTCCAT ATACTGTGCCTGAAT
8892   GCCAGTGTTTCAGAG TTCTCAATGAATGTC TGTTTCAATTAGAAT GCATTTGAAATAAGC TCCTAAAAAGCTCTA
8967   ATTAAGTTATTTATT TATGTATTTATTACG TATTTATTATATTTT CATTTCTGCACATGG ACTGTTCCGAAACCG
9042   TGATAATAAAGTTGC ATTTAAAAG
```

**Fig. S6. Prediction of the chinook salmon *IL2A* gene.** The chinook salmon *IL2A* was predicted on WGS contig PIPH01030521 using the Atlantic salmon and rainbow trout *IL2A* gene as bait. The gt/ag motifs at the intron boundaries are boxed. The start and stop codons for translation are highlighted in red. Putative mRNA instability motifs (ATTTA) and the polyadenylation signal in the 3'-UTR are in bold and boxed. A predicted signal peptide is highlighted in green. A potential *N*-glycosylation site is in purple.

```
Exon 1
14902 CCTCCCTTCGTATACAC CTCACAGGACTCTGC CTACAAGACCTTCAG ATTATCATGGAAAAC ATATTCAGGATTGCC
                                                               M  E  N   I  F  R  I  A
14827   TTTTTGATGCTTTTT CTCTCAGTTGGTCTT CAAGGAAGTCCGGCT AACAATTTCTTGAAA ATTGCAAAAGGAATC
         F  L  M  L  F   L  S  V  G  L   Q  G  S  P  A   N  N  F  L  K   I  A  K  G  I
14752   GAACGACTACAAATA AATATTACATGTgtaag
         E  R  L  Q  I   N  I  T  C

Exon 2
14636   tgtagCCAGAAGAAGGT ACATTCTTCTATGCT CCGTCTCCAGCGGAT GTAGAGgtaag
              P  E  E  G   T  F  F  Y  A   P  S  P  A  D   V  E

Exon 3
14249 tgcagAAAAGCATTGCT GGAGCACTGGCCTGT TCTATTCAACAACTG AACCACCTCAATGAC AACAGAAATCTGCAG
            K  S  I  A   G  A  L  A  C   S  I  Q  Q  L   N  H  L  N  D   N  R  N  L  Q
14174   CATCATATCAACAGG ATTTTGACGGTCCTA CAAAAGACATCTGAT GATATCgtaag
         H  H  I  N  R   I  L  T  V  L   Q  K  T  S  D   D  I

Exon 4
13985 ctcagAGAACGGACTCG TCAGAATGCAGCCGT GAGAACCCGCTGTCC AAAAAGTCTTGCAAG GAGTTCATGGAGAAT
            R  T  D  S   S  E  C  S  R   E  N  P  L  S   K  K  S  C  K   E  F  M  E  N
13915   ATGATCAGTTTAGCT AAAGCTCTATCTGCC CAACGGTCCCCATGA TAAAATAGGTTCTAG CTCCCAGTTGCATGG
         M  I  S  L  A   K  A  L  S  A   Q  R  S  P  *
13840   GGGGAGGATGGGGTC ACAGGAAGTAGATTG TCATTGCACTGATAC TATATATTATCTCTA ACAATTTGTGTAACA
13765   CCAGCTTTTAAAATA TATATATATATATAT ATATATATGTCTAAC TGGATGTCCTTAACC TGGTATGAAAGCCCT
13690   ACATAATGAAGGAAG GCGCTACTGTAGGTT ATGTTTAATGATGTG ATATCCATATACTGT GCCTGAACTGCTCTG
13615   AATGCAAGTGTTTGA GTTCTCAATGTCTGT TAACTATAATGTATT TAAGCTTCTAAAAAG CCCTAATTAAGTTAT
13540   TTATTTATGTATTTA TTACATATTTATTAT ATTTTGATTTCTACA CATGGACTGTTCCTA TAACCTTGATAATAA
13465   ATTTGCAATTAAAAG
```

**Fig. S7. Prediction of the chinook salmon *IL2B* gene.** The chinook salmon *IL2B* was predicted on WGS contig PIPH01048965 using the Atlantic salmon and rainbow trout *IL2B* gene as bait. The gt/ag motifs at the intron boundaries are boxed. The start and stop codons for translation are highlighted in red. Putative mRNA instability motifs (ATTTA) and the polyadenylation signal in the 3'-UTR are in bold and boxed. A predicted signal peptide is highlighted in green. A potential *N*-glycosylation site is in purple.

```
Exon 1
2389875     AAACATTTACAGATC TCCCCTTGTGTACAC CTTACAGGACACTAC CTACAAGCCCTTCAG ATTATCATGGACCGT
                                                                                    M  D  R
2389950     CTTTACAGGATTTCC TTTTTGACGCTTTTT CTCGCCGGTGGTCTA CAAGGAAACCCAATT AACAGACTCGAGTAT
             L  Y  R  I  S   F  L  T  L  F   L  A  G  G  L   Q  G  N  P  I   N  R  L  E  Y
2390025     CTAGAAAAAAATGCA ATCGTTGGAATCGAG TATCTAGAAAAAGAA ATTACATGTgtaag
             L  E  K  N  A   I  V  G  I  E   Y  L  E  K  E   I  T  C

Exon 2
2390168 tgtagCCAGATTCAGTCTTC TATACTCCAACTGAT GTAAAGgtaag
               P  D  S  V  F   Y  T  P  T  D   V  K

Exon 3
2390540 tccagGGTAGTTGCATTGTT GCAGCATTGGCCTGT TCCATTAAGGAACTG TACACTGTGAAAGAA GAATGCCTCGATAAC
               G  S  C  I  V   A  A  L  A  C   S  I  K  E  L   Y  T  V  K  E   E  C  L  D  N
2390615      ACGACCAAACTGGTA AATATGCAACACCAG ATCAACATGACTGCC AAGGACCTGCAAAAG ATGATTGATGAGGAG
               T  T  K  L  V   N  M  Q  H  Q   I  N  M  T  A   K  D  L  Q  K   M  I  D  E  E
2390690      AACgtaag
               N

Exon 4
2390796 ctcagAGCACAACGGACACT TCAGAATGCATCTGT GAAGACACACGGTTT GAAAAGTCTTTCAAG GACTTCATTCAGAAC
               S  T  T  D  T   S  E  C  I  C   E  D  T  R  F   E  K  S  F  K   D  F  I  Q  N
2390871      ATAAGACATTTAACT GGAGCTTATGCTGAA AAGCGTGGATAAGTC CATAAATGCTGGCAA TAGAGTGATTGTTGT
               I  R  H  L  T   G  A  Y  A  E   K  R  G  *
2390946      AATATATGGAACATT ACGTAATTGGGACAA AATACATGCCATACC AAATTGCACAGTAGT ACCATGTCTATCTGT
2391021      TCCAGTGATAAAGAA GCTCTAGTAGCTTCC AGTTGCTGAACAGTT ATATGGGGGGGGGGG GGGGGGGGGTTATAG
2391096      GAAGTTTGTCATTGC ACCGGTACTCTAATG TGTATAATCTATAAT CTCTAACAATTAGAG GCCCATTTTTTTATG
2391171      TGAAAAACAGTTAAT TTCATTGGTCATGTA TGTCTCACTGGATGT CCTTAACCGGAGATA GCAAGCCCTCCATAA
2391246      TGAAGGAAGGTGCTA CGGATGTTATTAATT ATGTTTTGTTTCATT ATGTGATTTCCATAT ACTGTGCCTGAATGC
2391321      CAGTGTTTCAGAGTT CTCAATGTCTGTTTC AATTAGAATGCATTT GAAATTATCTCCTAA AAAGCTCTAATTAAG
2391396      TTATTTATTTATGTA TTTATTACGTATTTA TTATATATTTTCATT TCTGCACATGGACTG TTCCGAAACCTTGAT
2392471      AATAAAGTTGCATTT AAAAGT
```

**Fig. S8. Prediction of the Arctic char *IL2A* gene.** The Arctic char *IL2A* was predicted on WGS contig PIPH01048965 using the Atlantic salmon and rainbow trout *IL2B* gene as bait. The gt/ag motifs at the intron boundaries are boxed. The start and stop codons for translation are highlighted in red. Putative mRNA instability motifs (ATTTA) and the polyadenylation signal in the 3'-UTR are in bold and boxed. A predicted signal peptide is highlighted in green. Potential *N*-glycosylation sites are in purple.

**Exon 1**

```
1420749      TCCCTTCGTGTACAC CTCACAGGACACTGC CTACAAGACCTTCAG ATTATCATGGAAAAC ATTTTCAGGATTGCC
                                                                   M  E  N   I  F  R  I  A
1420824      TTTTTGATGCTTTTT CTCTCAGTTGGTCTT CAAGGACTTCCGACT AAGAATTTCATGACA GTTCAACATGGAATC
              F  L  M  L  F   L  S  V  G  L   Q  G  L  P  T   K  N  F  M  T   V  Q  H  G  I
1420979      AAACATCTACAAAAA CAAATTACATGTgtaag
              K  H  L  Q  K   Q  I  T  C
```

**Exon 2**

```
1421018 agcagCCAGAAGGTACAGTC TTCTATGCTCCGTCT CCAGCGGATGTAGAG gtaag
              P  E  G  T  V   F  Y  A  P  S   P  A  D  V  E
```

**Exon 3**

```
1421399 tgcagAAAAGCATTGCTGGA GCACTGGCCTGTTCT ATTCAACAACTGAAC CACCTCAATAACACC CCAAATCTGCAGCAT
              K  S  I  A  G   A  L  A  C  S   I  Q  Q  L  N   H  L  N  N  T   P  N  L  Q  H
1421474      CATATCAACATGACT TTGGCAGTCCTACAA GCGACATTTGTTGAT GACACCgtaag
              H  I  N  M  T   L  A  V  L  Q   A  T  F  V  D   D  T
```

**Exon 4**

```
1421659 ctcagACAACGGACTCGTCA GAATGCAGCCGTGAG AACCCGCTGTCCAAA AAGTCTTGCAAGGAG TTCATGAAGAATATG
              T  T  D  S  S   E  C  S  R  E   N  P  L  S  K   K  S  C  K  E   F  M  K  N  M
1421734      ACCAGTTTAGCTGAA GCTCTATTTGTCAAA TATTCCCAATCATAA AATAGGTTCTAGCTC CCAGTTAAAATGGGG
              T  S  L  A  E   A  L  F  V  K   Y  S  Q  S  *
1421809      GGAGGATGGGGTCAC AGGAAGTAGATTGTC ATTGCACTGATACTA TATATTATCTCTAAC AATTTGTGTAACATT
1421884      GTGTGACACCAGCTT TTAAAGCATATTTAT ATATATTTTTTTTTT TATATGTCTAACTGG ATGTCCTTAAAAACC
1421959      TGGGATGAAAGCCCT ACATAATGAAGGAAA GGGCTACTGTAGGTT CTGTTTAATGATGTG ATATCCATATACTGT
1422034      GTCTGAACTGCTCTG AATGCAAGTGTTTGA GTTCTCAATGTCTGT TAACTATAATGTATT TAAGCTTCTAAAAAG
1422109      CTCTAATTAAGTTAT TTATTTATGTATTTA TTACATATTTATTAT ATTTTGATTTCTACA CATGGTCTGTTCCTA
1422184      TAACCTTGACAATAA ATTTGCAATTAAAAG
```

**Fig. S9. Prediction of the Arctic char *IL2B* gene.** The Arctic char *IL2B* was predicted on WGS contig PPUY01016320 using the Atlantic salmon and rainbow trout *IL2B* gene as bait. The gt/ag motifs at the intron boundaries are boxed. The start and stop codons for translation are highlighted in red. Putative mRNA instability motifs (ATTTA) and the polyadenylation signal in the 3'-UTR are in bold and boxed. A predicted signal peptide is highlighted in green. Potential *N*-glycosylation sites are in purple.

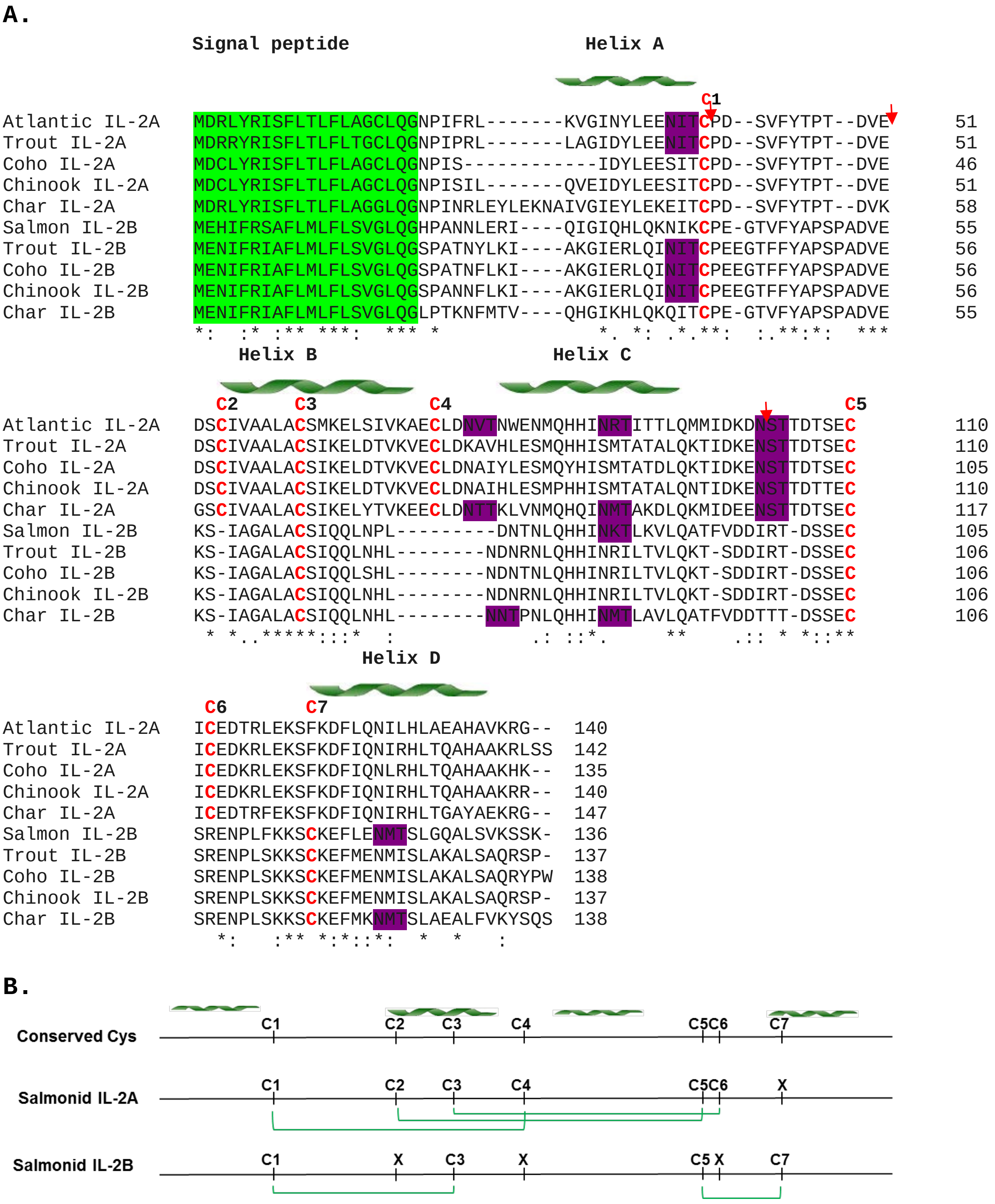


**Fig. S10. Amino acid sequence alignment of salmonid IL-2 (A) and predicted potential intra-molecular disulfide bonds (B).** (**A**) The multiple alignment was produced using ClustalW. Gaps introduced to increase identity are shown by dashes. The completely conserved residues and similar residues are indicated by asterisks and dots (. or :), respectively, below the sequences. The conserved cysteine residues are in red and numbered above the alignment (C1-7). The predicted four α helices (A–D) are indicated above the alignment. The predicted signal peptide is highlighted in green and potential *N*-glycosylation sites are in purple. The arrows indicate intron positions. (**B**) Conserved cysteine residues in salmonid IL-2A and IL-2B. Cysteine residues potentially forming disulfide bonds are linked by green lines. The position of the four α helices are also indicated.

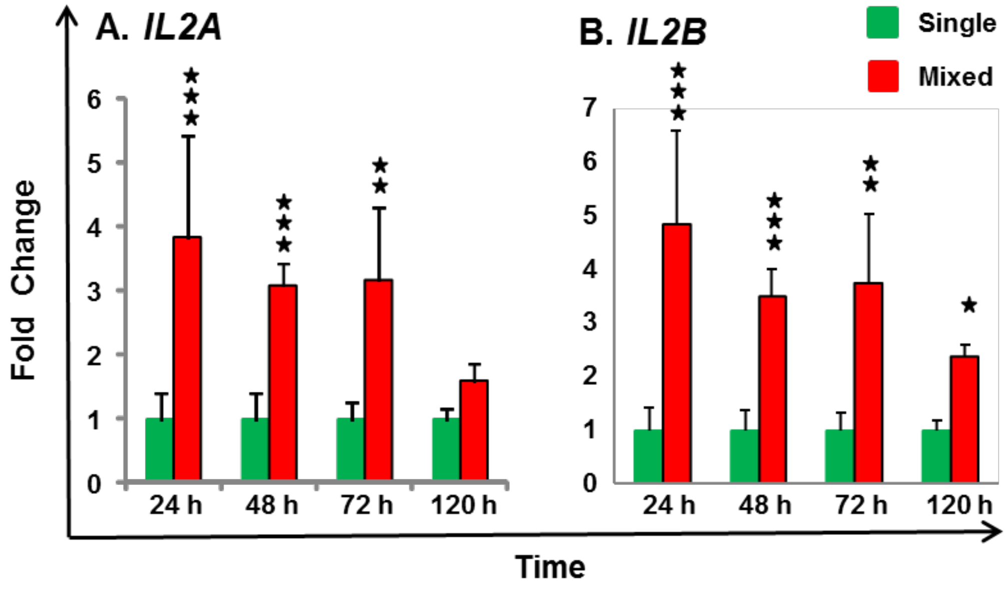


**Fig. S11. Mixed leucocyte reaction induced *IL2* expression in rainbow trout head kidney (HK) cells**. HK cells were prepared from 6 fish (1-6) and incubated at 20 °C individually (Single) or as a mix of three fish (Mixed, 123, 234, 345, 456, 561 and 612) for 24 h, 48 h, 72 h and 120 h. The expression of *IL2* paralogues was measured by RT-qPCR. The data are presented as a mean (+SEM) fold change calculated by the average expression level of mixed HK cells divided by that of single fish HK cells at the same time point. The relative significance of a LSD post hoc test after a significant one-way ANOVA between the mixed and single HK cells at the same time point is shown above the bars as * $p \leq 0.05$; **$p \leq 0.01$ and *** $p \leq 0.001$.

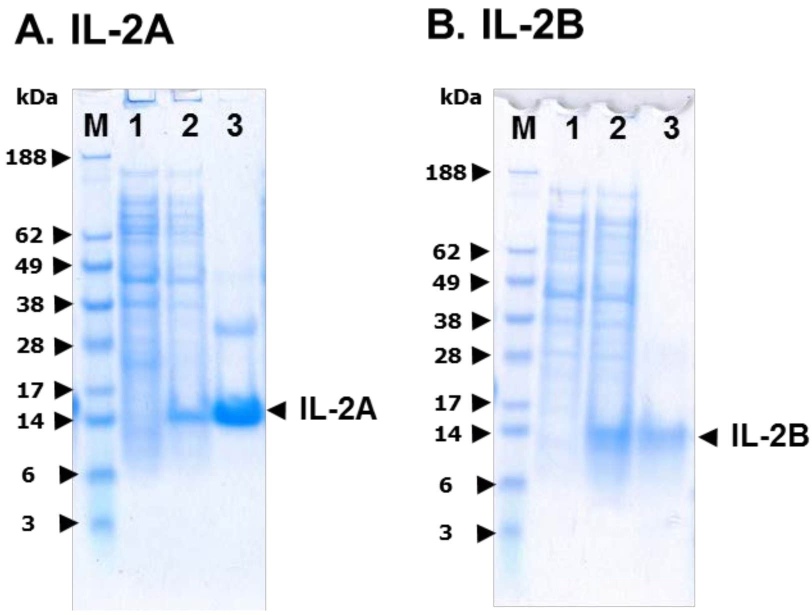


**Fig. S12. SDS-PAGE analysis of rainbow trout IL-2A (A) and IL-2B (B) recombinant proteins.** The recombinant proteins were expressed in *E. coli* BL21 Star (DE3) by IPTG induction. Samples were analysed using NuPAGE 4-12% Bis-Tris gel (Invitrogen) and stained with PageBlue protein staining solution (Thermo Scientific). 1. Transformed cells without IPTG induction; 2. Transformed cells induced with 1 mM IPTG for 4 h; 3. Purified recombinant proteins; M. Protein marker, SeeBlue (Invitrogen).

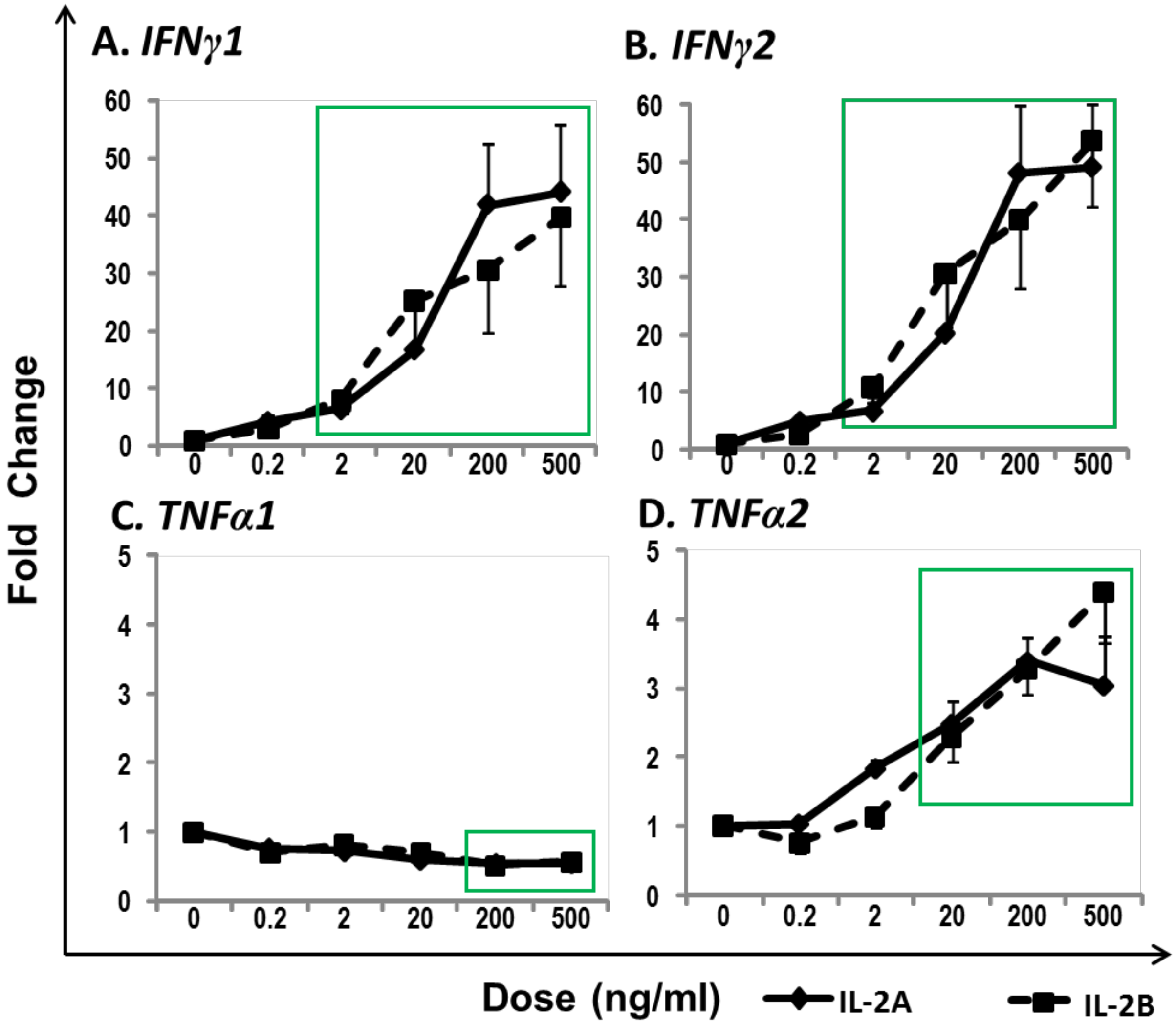


**Fig. S13. Modulation of the expression of *IFNγ1* (A), *IFNγ2* (B), *TNFα1* (C) and *TNFα2* (D) by IL-2 isoforms in HK cells.** Freshly prepared HK cells were incubated with 0.2-500 ng/ml IL-2A, IL-2B, or storage buffer (0) as control for 24 h. Expression was quantified by RT-qPCR, and presented as arbitrary units where one unit equals the average expression level in the control samples. The treatments grouped in the green box are significantly different from control samples (Paired samples T test, $p \leq 0.05$). Data are means + or – SEM of HK cells from four fish.

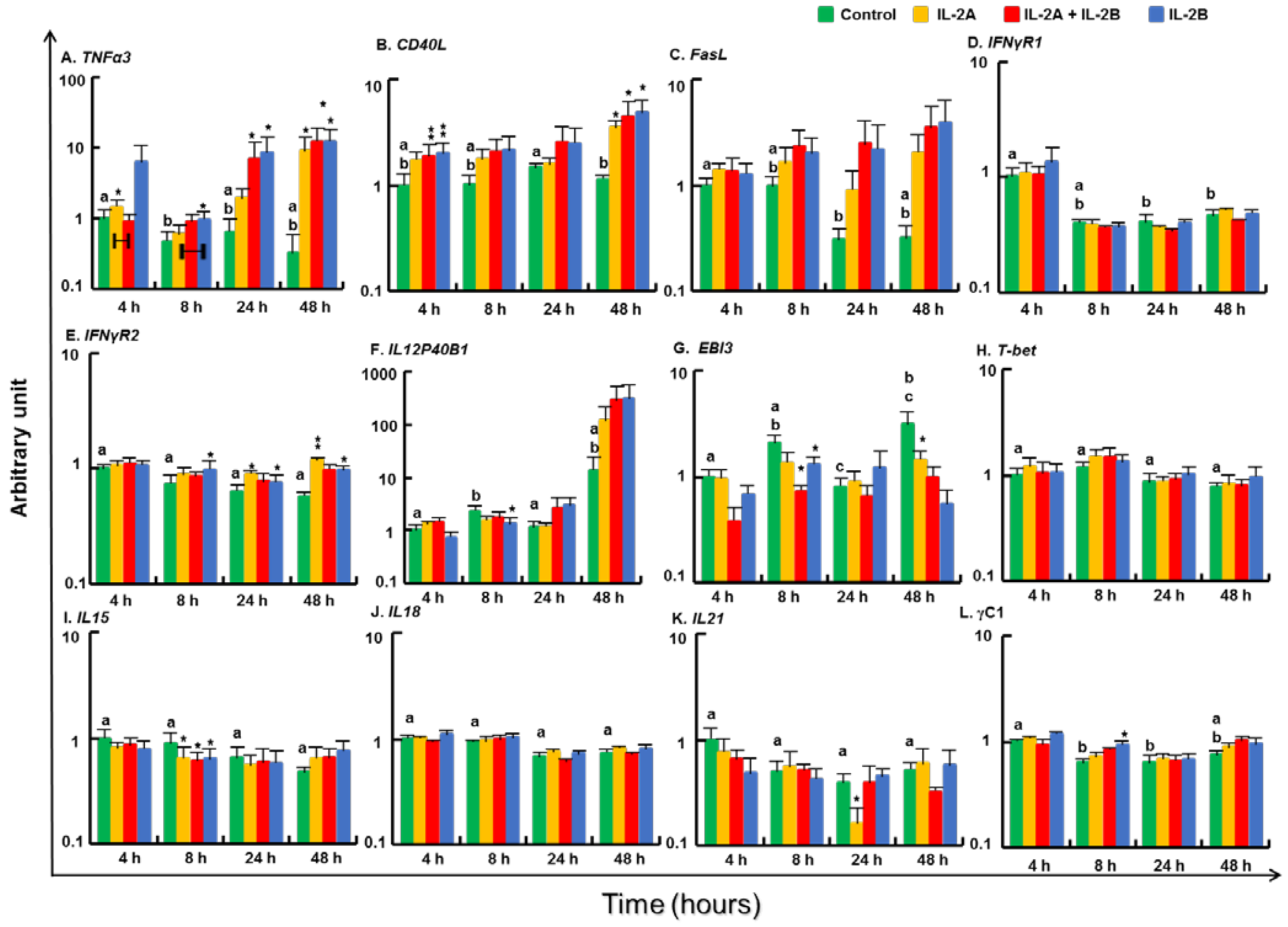


**Fig. S14. Modulation of other Th1 pathway gene expression by IL-2 isoforms in PBL.** Freshly prepared PBL from 4 fish were incubated with 200 ng/ml IL-2A, IL-2B, or both (IL-2A+IL-2B), or with medium alone as control for 4 h, 8 h, 24h and 48 h. The average (+SEM) expression of *TNFα3* (A), *CD40L* (B), *FasL* (C), *IFNγR1* (D), *IFNγR2* (E), *IL12P40B1* (F), *EBI3* (G), *T-bet* (H), *IL15* (I), *IL18* (J), *IL21* (K) and *γC1* (L) was quantified by RT-qPCR. The gene expression was expressed as arbitrary units where one unit equals the average expression level in the control samples at 4 h. Significant results of a paired samples T test between the stimulated samples and time-matched controls are shown above the bars as $*p \leq 0.05$ and $**p \leq 0.01$. Different letters over the control bars indicate significant differences over time in the unstimulated cells ($p \leq 0.05$). The "H" connecting two treatment bars indicates a significant difference ($p \leq 0.05$).

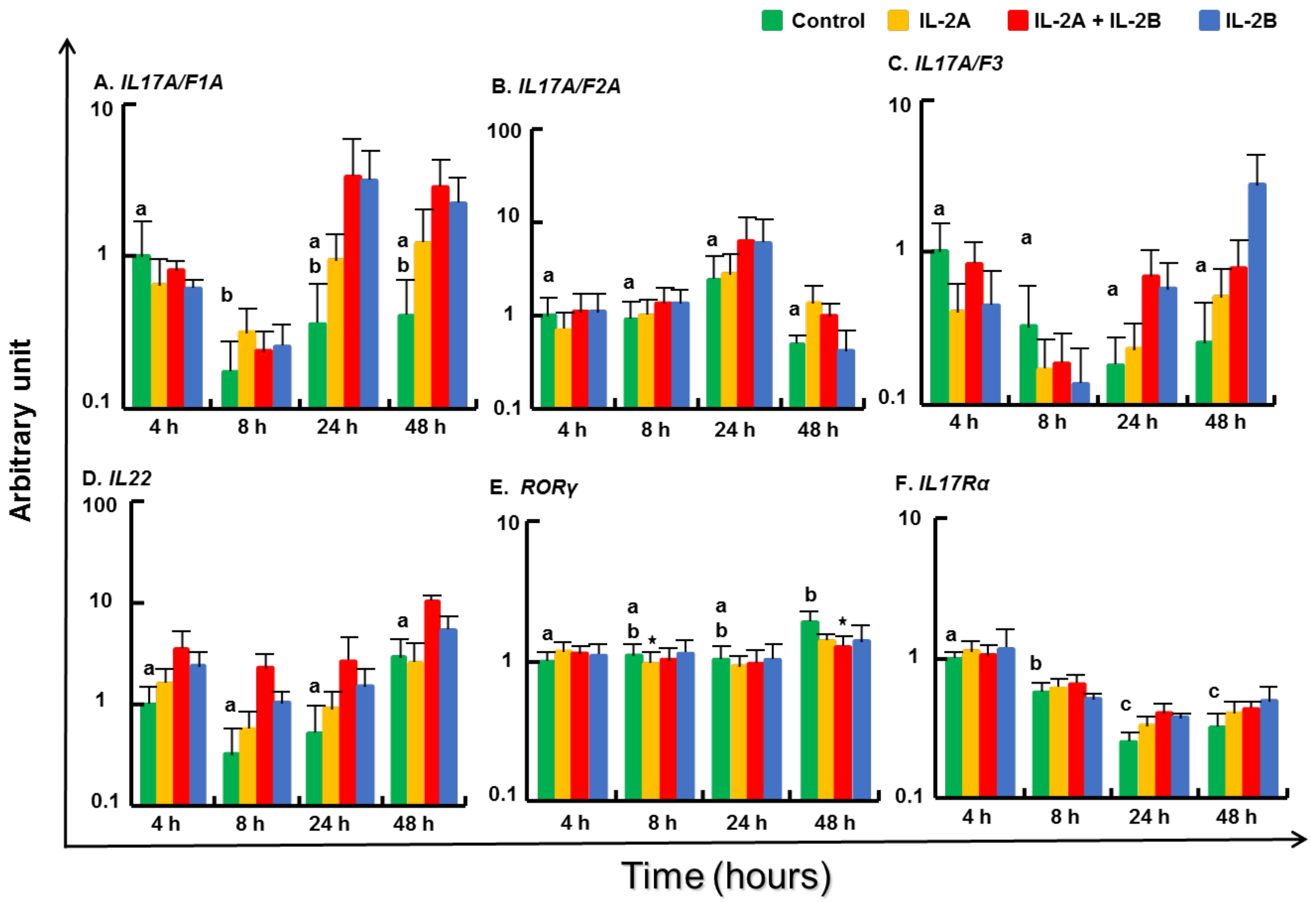


**Fig. S15. Modulation of Th17 pathway gene expression by IL-2 isoforms in PBL.** Freshly prepared PBL from 4 fish were incubated with 200 ng/ml IL-2A, IL-2B, or both (IL-2A+IL-2B), or with medium alone as control for 4 h, 8 h, 24 h and 48 h. The average (+SEM) expression of *IL17A/F1A* (A), *IL17A/F2A* (B), *IL17A/F3* (C), *IL22* (D), *RORγ* (E) and *IL17Rα* (F) was quantified by RT-qPCR. The gene expression was expressed as arbitrary units where one unit equals the average expression level in the control samples at 4 h. Significant results of a paired samples T test between the stimulated samples and time-matched controls are shown above the bars as *$p \leq 0.05$. Different letters over the control bars indicate significant differences over time in the unstimulated cells ($p \leq 0.05$).

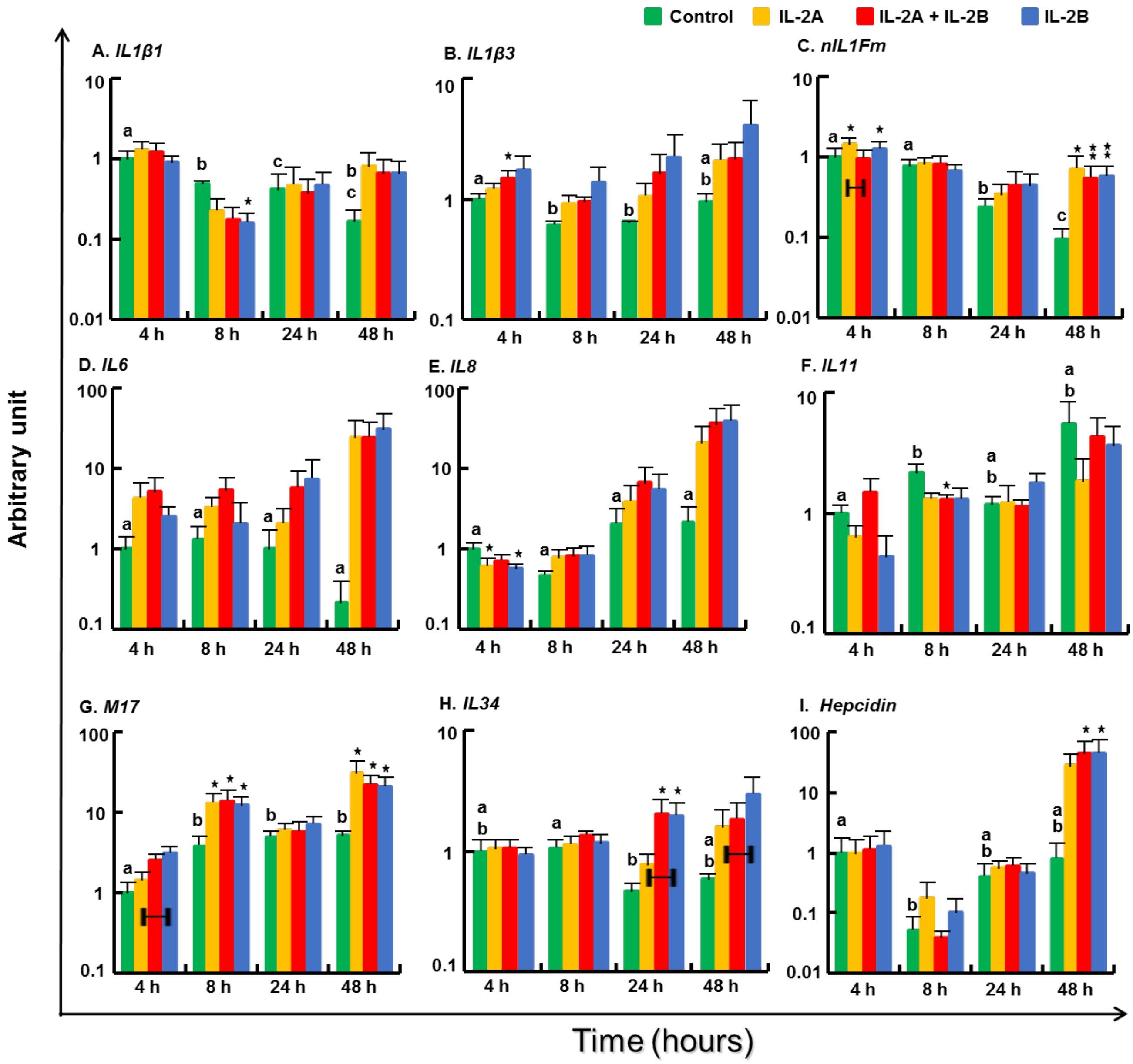


**Fig. S16. Modulation of proinflammatory cytokine and hepcidin gene expression by IL-2 isoforms in PBL.** Freshly prepared PBL from 4 fish were incubated with 200 ng/ml IL-2A, IL-2B, or both (IL-2A+IL-2B), or with medium alone as control for 4 h, 8 h, 24 h and 48 h. The average (+SEM) expression of *IL1β1* (A), *IL1β3* (B), *nIL1Fm* (C), *IL6* (D), *IL8* (E), *IL11* (F), *M17* (G), *IL34* (H) and Hepsidin (I) was quantified by RT-qPCR. The gene expression was expressed as arbitrary units where one unit equals the average expression level in the control samples at 4 h. Significant results of a paired samples T test between the stimulated samples and time-matched controls are shown above the bars as $*p \leq 0.05$ and $**p \leq 0.01$. Different letters over the control bars indicate significant differences over time in the unstimulated cells ($p<0.05$). The "H" connecting two treatment bars indicates a significant difference ($p \leq 0.05$).

**Table S1 Primers used for cloning, expression analysis and recombinant protein production of salmonid *IL2A* and *IL2B* genes**

| Gene | Primer | Sequences(5'- to 3') | Application |
|---|---|---|---|
| **Atlantic salmon *IL2B*** | **sIL2BF1** | ACAGACTCCAGACCTCCCTTCGT | 3'-RACE , PCR |
| | **sIL2BF2** | CCTCCCTTCGTGTACATCTCACA | 3'-RACE |
| | **sIL2BR1** | GCAGTTCAGGCACAGCATATGG | PCR |
| **Trout *IL2A*** | **rF** | AACCCAATTCCCAGACTCCTAG | Recombinant protein |
| | **rR** | TGAACTTAGACGCTTTGCAGC | Recombinant protein |
| | **F** | TGATGTAGAGGATAGTTGCATTGTTGC | Real-time PCR |
| | **R** | GAAGTGTCCGTTGTGCTGTTCTC | Real-time PCR |
| **Trout *IL2B*** | **rF** | AGCCCGGCTACCAATTACTTGA | Recombinant protein |
| | **rR** | TGGGGACCGTTGGGCAGAT | Recombinant protein |
| | **F** | TTCTTCTATGCTCCGTCTCCAGC | Real-time PCR |
| | **R** | CATTCTGACGAGTCCGTTCTGATATC | Real-time PCR |

# Table S2. Summary of sequence analysis of salmonid IL-2

| Gene | IL2A | | | | | IL2B | | | | |
|---|---|---|---|---|---|---|---|---|---|---|
| Species | Atlantic | Trout | Coho | Chinook | Char | Atlantic | Trout | Coho | Chinook | Char |
| Nucleic acid sequence | | | | | | | | | | |
| WGS contigs | AGKD04000200 | MSJN01001230 | MPKV01002376 | PIPH01030521 | PPUY01000011 | AGKD04000795 | MSJN01001294 | MPKV01002536 | PIPH01048965 | PPUY01016320 |
| No. of exons | 4 | 4 | 4 | 4 | 4 | 4 | 4 | 4 | 4 | 4 |
| No. of ATTTA motifs | 5 | 5 | 5 | 5 | 5 | 5 | 6 | 7 | 4 | 6 |
| Protein sequence | | | | | | | | | | |
| Full length (aa) | 140 | 142 | 135 | 140 | 147 | 136 | 137 | 138 | 137 | 138 |
| Signal peptide | 20 | 20 | 20 | 20 | 20 | 20 | 20 | 20 | 20 | 20 |
| Mature peptide (MP) | 120 | 122 | 115 | 120 | 127 | 116 | 117 | 118 | 117 | 118 |
| MW (kDa) of MP | 13.7 | 13.7 | 13.0 | 13.5 | 14.5 | 12.8 | 13.0 | 13.2 | 13.0 | 13.1 |
| pI of MP | 4.83 | 5.11 | 4.65 | 4.76 | 4.85 | 7.87 | 7.66 | 6.79 | 7.67 | 7.12 |
| N-glycosylation sites | 4 | 2 | 1 | 1 | 3 | 2 | 1 | 1 | 1 | 3 |
| No. of Cys | 6 | 6 | 6 | 6 | 6 | 4 | 4 | 4 | 4 | 4 |
| Homology table | | | | | | | | | | |
| 1. Atlantic IL-2A | | 77.5 | 75.0 | 77.1 | 74.1 | 42.9 | 41.9 | 42.3 | 41.9 | 42.6 |
| 2. Trout IL-2A | 83.1 | | 85.9 | 88.0 | 73.2 | 41.2 | 41.6 | 40.9 | 41.6 | 40.5 |
| 3. Coho IL-2A | 82.1 | 89.4 | | 90.0 | 72.1 | 40.1 | 39.2 | 38.9 | 39.2 | 39.9 |
| 4. Chinook IL-2A | 85.0 | 90.1 | 93.6 | | 72.8 | 39.5 | 39.2 | 38.9 | 39.2 | 40.5 |
| 5. Char IL-2A | 79.6 | 79.6 | 78.9 | 79.6 | | 43.0 | 40.1 | 40.5 | 40.8 | 41.4 |
| 6. Atlantic IL-2B | 67.9 | 63.4 | 65.4 | 65.0 | 63.9 | | 76.1 | 74.8 | 76.8 | 79.0 |
| 7.Trout IL-2B | 66.4 | 63.4 | 65.7 | 65.7 | 59.2 | 81.8 | | 96.4 | 98.5 | 74.8 |
| 8. Coho IL-2B | 66.4 | 62.0 | 64.5 | 65.0 | 59.9 | 81.2 | 97.8 | | 96.4 | 74.1 |
| 9. Chinook IL-2B | 66.4 | 63.4 | 65.7 | 65.7 | 59.9 | 82.5 | 99.3 | 97.1 | | 75.5 |
| 10. Char IL-2B | 67.1 | 63.4 | 65.2 | 65.7 | 63.3 | 84.1 | 81.2 | 80.4 | 81.2 | |

# Table S3. Primers used for real-time RT-PCR analysis of gene expression

| No. | Gene | ΔCP* | Forward (5'-3') | Reverse (5'-3') | Size (bp) | Acc. no. |
|---|---|---|---|---|---|---|
| House keeping gene | | | | | | |
| 1 | EF1α | | CAAGGATATCCGTCGTGGCA | ACAGCGAAACGACCAAGAGG | 327 | AF498320 |
| Th1 pathway related genes | | | | | | |
| 2 | IFNγ1 | 20.7 | CAAACTGAAAGTCCACTATAAGATCTCCA | TCCTGAATTTTCCCCTTGACATATTT | 210 | AJ616215 |
| 3 | IFNγ2 | 22.6 | CAAACTGAAAGTCCACTATAAGATCTCCA | GGTCCAGCCTCTCCCTCAC | 188 | FM864345 |
| 4 | IFNγR1 | 9.8 | CAGACGTGGTGGTACATTGCTCAT | AGCAGGTAAAACTATGCTCTTTCAACG | 201 | CCAF010024601 |
| 5 | IFNγR2 | 11.7 | CAGACAGGCCAGGGATAAAGT | TACCATGTGGACCATCAGAAG | 251 | EU244877 |
| 6 | CXCL11_L1 | 19.2 | TCATCAGCTTCCTGGCCTGTC | TTCTCCGTTCTTCAGAGTGACAATGAT | 191 | AF396869 |
| 7 | TNFa1 | 16.5 | TGTGTGGGGTCCTCTTAATAGCAGGTC | CCTCAATTTCATCCTGCATCGTTGA | 102 | AJ277604 |
| 8 | TNFa2 | 18.2 | CTGTGTGGCGTTCTCTTAATAGCAGCTT | CATTCCGTCCTGCATCGTTGC | 98 | AJ401377 |
| 9 | TNFa3 | 15.9 | GCTGCACTCTTCTTTACCAAGAAACAAG | CCACTGAGGACTTGTAATCACCATAGGT | 148 | HE798544 |
| 10 | CD40L | 17.5 | AAAACAAGCATGACGAGGACTTTG | CAACTTGTGAGACCTTTTCCATTATGTT | 156 | EF160131 |
| 11 | FasL | 18.3 | GGACCAAGGTTAACATTGAGAGCCA | CCTTCTCGATCCGGCCTATGAC | 133 | BX857476 |
| 12 | IL12P35A1 | 15.1 | GGAACACCACATTCAGTGAGAGTGC | CGTCTGCAACTTGTGAGGAAGGAT | 180 | HE798148 |
| 13 | IL12P35A2 | 20.2 | GGAACACCACATTCAGTGAGAGTGA | CAACCTGTGAGGAAGACACCCA | 174 | HG917950 |
| 14 | IL27A1 | 19.7 | GCAGCTGCTCAGGAGATATAAGGAGG | TCTCTCAGGTATGCTGGGTTTTGG | 277 | HG794528 |
| 15 | IL23P19 | 21.8 | ACCTAAGAGCAGATTCAATGCCTTG | TCTTCCCAGCTCTTCACTTCCTG | 211 | KP410548 |
| 16 | IL12P40B1 | 18.9 | CCCTTCTACATCCGAGAAATAGTGAAAC | GTTGGTTTCACTTATAAACACCTTTTCCTT | 195 | HE798149 |
| 17 | IL12P40B2 | 16.6 | CCGTTCTACATACGAGAAATAGTGGAGA | TCAGAGTCACAGCTTTCCCTGG | 167 | HG917952 |
| 18 | IL12P40C | 16.4 | TTAAAGACAACGGAAAGGAGGAGC | CCTCCCGTAACCACATTTTTCC | 143 | AJ548830 |
| 19 | EBI3 | 18.1 | ACATCGCCACCTACAGTATGAAAGG | GGGTCCGGCTTCACAATGT | 257 | AJ620467 |
| 20 | T-bet | 11.8 | GGTAACATGCCAGGGAACAGGA | TGGTCTATTTTTAGCTGGGTGATGTCTG | 317 | FM863825 |
| 21 | IL15 | 10.6 | TGGAATTGCTTCATAATATTGAGCTGCC | TGGTAGTTATCTGTGACCGACATGTCCTC | 278 | AJ628345 |
| 22 | IL18 | 8.9 | GAGCAATGCAAAGCAGATGATTG | CATGTTTTGAGCAGCCAATGTAGTC | 211 | AJ556990 |
| 23 | IL21 | 16.8 | AAAGTTATCAAAAACCTCAACAACCGAA | CCAGTCTACTGATGGCCTTTTGAAG | 169 | FM883702 |
| Th2 pathway related genes | | | | | | |
| 24 | IL4/13A | 15.1 | ACCACCACAAAGTGCAAGGAGTTCT | CACCTGGTCTTGGCTCTTCACAAC | 156 | FN820501 |
| 25 | IL4/13B1 | 21.3 | GAGATTCATCTACTGCAGAGGATCATGA | GCAGTTGGAAGGGTGAAGCTTATTGTA | 261 | HG794522 |
| 26 | IL4/13B2 | 21.0 | GAGACTCATCTATTGCGTATGATCATCG | TGCAGTTGGTTGGATGAAACTTATTGTA | 262 | HG794523 |
| 27 | IL4Ra1 | 14.4 | CTGAATACAGCATGACGTTGAAACA | GGGGTTTTGGGTTTTATGGTGT | 226 | AJ634731 |
| 28 | IL4Ra2 | 11.4 | GTGCTGAATACAACATGACATTGGAAT | GGGTTTTGGGTTTTATGCTGC | 228 | FN824519 |
| 29 | GATA3 | 15.7 | CCA AAA ACA AGG TCA TGT TCA GAA GG | TGGTGAGAGGTCGGTTGATATTGTG | 313 | FM863826 |
| Th17 related genes | | | | | | |
| 30 | IL17A/F1A | 19.7 | CAAACGTACACTTTTTGATGGTGCTG | GGGACTCATCATAGGTGGTGTTGGT | 280 | KJ921977 |
| 31 | IL17A/F2A | 21.3 | CACCCTGGACCTGGAAAAGCAC | GGCCACAGACAGGAAGGAGG | 212 | AJ580842 |
| 32 | IL17A/F3 | 20.4 | CTGGTGCTGGGTCTGATCATGT | GGTCTCATCGTATGTGTCGCTGTATG | 222 | KJ921980 |
| 33 | IL-22 | 22.6 | GAAGGAACACGGCTGTGCTATTAAAC | GATCTAGGCGTGCACACAGAAGTC | 168 | AM748538 |
| 34 | IL17C1 | 22.6 | CTGGCGGTACAGCATCGATA | GAGTTATATCCATAATCTTCGTATTCGGC | 138 | FM955455 |
| 35 | IL17C2 | 19.1 | CTGGCGGTACAGCATCGATA | CAGAGTTATATGCATGATGTTGGGC | 134 | FM955456 |
| 36 | IL17D | 22.5 | GAAGAAATCCTCGAGCAGATGTTTG | GGGTCGTGGGAGATCCTGTATG | 200 | AJ580843 |
| 37 | IL17Rα | 13.7 | CGTAATCCAGCATATGAAAAGTGGTCTT | CTCCAAACACACCTTGCTCCTCT | 192 | AJ634727 |
| 38 | RORγ | 15.1 | ACAGACCTTCAAAGCTCTTGGTTGTG | GGGAAGCTTGGACACCATCTTTG | 262 | FM883712-13 |

Table S3, continued

| No. | Gene | ΔCP* | Forward (5'-3') | Reverse (5'-3') | Size (bp) | Acc. no. |
|---|---|---|---|---|---|---|
| Regulatory pathways | | | | | | |
| 39 | TGFβ1A | 8.0 | CTCACATTTTACTGATGTCACTTCCTGT | GGACAACTGCTCCACCTTGTG | 371 | OMY7836 |
| 40 | TGFβ1B | 9.2 | CATGTCCATCCCCCAGAACT | GGACAACTGTTCCACCTTGTGTT | 361 | FN822750 |
| 41 | IL10A | 20.7 | GGATTCTACACCACTTGAAGAGCCC | GTCGTTGTTGTTCTGTGTTCTGTTGT | 119 | AB118099 |
| 42 | IL10B | 20.6 | GGGATTCTAGACCACATCAAGAGTCC | GATGGGAGATTTAAAGTTGTGTGTTCC | 132 | FR691804 |
| 43 | FOXP3A | 15.3 | CCCAGAACCGAGGTGGAGTGT | TGACGGACAGCGTTCTTCCA | 319 | FM883710 |
| 44 | FOXP3B | 15.1 | TCCTGCCCCAGTACTCATCCC | TGACGGACAGCGTTCTTCCA | 295 | FM883711 |
| IL-2 and IL2 receptor subunit | | | | | | |
| 45 | IL2A | 18.0 | TGATGTAGAGGATAGTTGCATTGTTGC | GAAGTGTCCGTTGTGCTGTTCTC | 180 | AM422779 |
| 46 | IL2B | 22.5 | TTCTTCTATGCTCCGTCTCCAGC | CATTCTGACGAGTCCGTTCTGATATC | 176 | HE805273 |
| 47 | CD25L | 16.9 | TGT GCATACCCGACCCATCTA | TGCATATATGGCTGTAGCAGTGC | 344 | NM_001124572 |
| 48 | IL2Rβ1 | 11.6 | CGGTGGAAACTTTCAGAAATGGCT | AAGAGGCTGCTGGGGTATTTGGT | 261 | FN813346 |
| 49 | IL2Rβ2 | 10.4 | CGGTGGAAACTTTCAGAAATGGCT | GGAAGCCGCTGGGGTAGAAGTA | 251 | FN813347 |
| 50 | γC1 | 5.6 | TTTCAGCAGCAGGTTC ATCAAAGA | AGGAGGGTAAAGCTTCACCATGCT | 196 | AJ276623 |
| 51 | γC2 | 12.6 | CTTCCACAAAGGGTCCATCAAAAG | AGGAGGGTAAAGCTTCACCATGCT | 196 | FN813348 |
| T cell marker genes | | | | | | |
| 52 | CD3e | 11.7 | AAGGAAAGGTGTGTAAGGACTGCTATG | GAGCGGGAAGTGGGTTTTTG | 160 | NM_001195174 |
| 53 | CD4-1 | 11.7 | GTGTGGAGGTGCTACAGGTTTTTTC | ATCGTCACCCGCTGTCTGTG | 396 | AY973028 |
| 54 | CD4-2A | 13.3 | CGACATTGTCACAGTCAAGGTCC | CCTCATTTGGCAACAAACTTCTCAC | 284 | AY973029 |
| 55 | CD4-2B | 13.9 | CGACATTGTCACAGTCAAGGTCC | GGCTTAGCAGTCAACAAACTTTTCAC | 285 | AY899932 |
| 56 | CD8α | 13.8 | CAAGTCGTGCAAAGTGGGAAA | TCTGTTGTTGGCTATAGGATGTTGTTG | 214 | AF178053 |
| 57 | CD8β | 15.0 | GAACTATCAAACCCCAGAAGGCTGTG | GACACTTTTTGGGTAGTCGGCTGAA | 125 | AY563420 |
| Chemokine receptors | | | | | | |
| 58 | CXCR1 | 8.9 | TGGATGCAAAGATGACAGAGGTCC | AAGCCAATCACGAGCCCCAC | 235 | AF260965 |
| 59 | CXCR2 | 14.2 | GGACATGTAAAGCCAGCTCATGG | AGGGTCAGGGAGAAGAGGAGGTC | 300 | HG794530 |
| 60 | CXCR3A | 13.3 | CACTGGAGCCATGTTTACAATCAACT | CCCTCACAGACTCCAGGAAGTG | 206 | AJ888881 |
| 61 | CXCR3B | 14.1 | CAAGGCAACCACAAATTACTATATTTATGATG | CAGCACACACAGCACCAGGAT | 193 | AJ888878 |
| 62 | CCR7A | 17.2 | TTTCACTGATTACCCCACAGACAATAC | GGAGATGGCGAAGTACCTGTCC | 394 | CU065128 |
| 63 | CCR7B | 9.8 | TACAATGGTTACTCCACAGACACGAA | TGGAGATGGCAAAGTACCTGTCC | 394 | CA376736 |
| Pro-inflammatory cytokines and antimicrobial peptide genes | | | | | | |
| 64 | IL1β1 | 12.6 | CCTGGAGCATCATGGCGTG | GCTGGAGAGTGCTGTGGAAGAACATATAG | 179 | AJ278242 |
| 65 | IL1β2 | 21.8 | GAGCGCAGTGGAAGTGTTGG | AGACAGGTTCAAATGCACTTTATGGT | 204 | AJ245925 |
| 66 | IL1β3 | 13.7 | CTG AAG GCC GTC ACA ATC CA | CTGGTCCTTACAGCGCTCCAA | 195 | AM181685 |
| 67 | nIL1Fm | 11.0 | CCCATTCCTCGTGACACCAG | CTGGACGACCTGGAGAGTGACT | 250 | AJ555869 |
| 68 | IL6 | 20.2 | GGGAGAAAATGATCAAGATGCTCGT | GCAGACATGCCTCCTTGTTGG | 180 | DQ866150 |
| 69 | IL8 | 12.5 | TCCTGACCATTACTGAGGGGATGA | AGCGCTGACATCCAGACAAATCTC | 200 | AJ279069 |
| 70 | IL11 | 19.8 | CTCTCGCTGCTATTGGCCCA | TCTCGAATGCATGTTCCTTCAATAGAT | 174 | AJ535687 |
| 71 | IL34 | 13.2 | AGGCAGAAGACGTAACATGAAACACA | TCCACCCTCGCCCTCAGCTT | 111 | FN820429 |
| 72 | M17 | 16.7 | GTGGACCTCTTAAAAACATACAAGCTCAG | GGATGGTGGCTGTAAGTCTGTCTG | 204 | FM866399 |
| 73 | CATH1 | 21.0 | ACCAGCTCCAAGTCAAGACTTTGAA | TGTCCGAATCTTCTGCTGCAA | 275 | AY594646 |
| 74 | CATH2 | 13.4 | ACATGGAGGCAGAAGTTCAGAAGA | GAGCCAAACCCAGGACGAGA | 135 | AY542963 |
| 75 | Hepcidin | 18.9 | GCTGTTCCTTTCTCCGAGGTGC | GTGACAGCAGTTGCAGCACCA | 165 | CA369786 |

Note

* Δcp, the average cp (crossing point at which the fluorescence crosses the threshold during qPCR, N=4) of a target gene minus that of the house keeping gene EF-1α in control PBL at 4 h is shown. The average cp of EF-1a is 11.9.

**Table S4. Characteristics (top) and comparison of amino acid identity / similarity (bottom) of IL-2 like molecules from salmonids, percomorphs, common carp and mammals. Bottom right, identity; bottom left, similarity.**

| | 1 | 2 | 3 | 4 | 5 | 6 | 7 | 8 | 9 | 10 | 11 | 12 | 13 | 14 | 15 | 16 | 17 | 18 | 19 | 20 | 21 |
|---|---|---|---|---|---|---|---|---|---|---|---|---|---|---|---|---|---|---|---|---|---|
| | Salmonid | | | | | | | | | | Percomorphs | | | | | | Carp | | Mammalian | | |
| Full length (aa) | 140 | 142 | 135 | 140 | 147 | 136 | 137 | 138 | 137 | 138 | 149 | 142 | 135 | 131 | 135 | 130 | 149 | 142 | 153 | 155 | 155 |
| Signal peptide | 20 | 20 | 20 | 20 | 20 | 20 | 20 | 20 | 20 | 20 | 22 | 22 | 19 | 19 | 19 | 20 | 20 | 20 | 20 | 20 | 20 |
| Mature peptide (MP) | 120 | 122 | 115 | 120 | 127 | 116 | 117 | 118 | 117 | 118 | 127 | 120 | 116 | 112 | 116 | 110 | 129 | 122 | 133 | 135 | 135 |
| MW (kDa) of MP | 13.7 | 13.7 | 13.0 | 13.5 | 14.5 | 12.8 | 13.0 | 13.2 | 13.0 | 13.1 | 14.1 | 13.9 | 13.1 | 12.5 | 13.1 | 12.4 | 14.8 | 13.8 | 15.4 | 15.5 | 15.5 |
| pI of MP | 4.83 | 5.11 | 4.65 | 4.76 | 4.85 | **7.87** | **7.66** | **6.79** | **7.67** | **7.12** | 4.56 | 5.03 | 4.94 | 4.53 | 5.67 | 4.51 | 4.29 | 5.51 | 7.05 | 5.74 | 6.34 |
| Homology table | | | | | | | | | | | | | | | | | | | | | |
| 1. Atlantic IL-2A | | **77.5** | **75.0** | **77.1** | **74.1** | 42.9 | 41.9 | 42.3 | 41.9 | 42.6 | 30.5 | 32.0 | 32.2 | 24.8 | 23.2 | 23.1 | 25.9 | 29.1 | 18.8 | 15.6 | 15.5 |
| 2. Trout IL-2A | 83.1 | | **85.9** | **88.0** | **73.2** | 41.2 | 41.6 | 40.9 | 41.6 | 40.5 | 30.9 | 31.3 | 31.1 | 27.3 | 23.9 | 24.3 | 25.2 | 25.8 | 18.5 | 16.0 | 19.0 |
| 3. Coho IL-2A | 82.1 | 89.4 | | **90.0** | **72.1** | 40.1 | 39.2 | 38.9 | 39.2 | 39.9 | 30.2 | 30.0 | 29.0 | 23.2 | 21.2 | 23.1 | 24.7 | 26.5 | 15.5 | 16.7 | 14.6 |
| 4. Chinook IL-2A | 85.0 | 90.1 | 93.6 | | **72.8** | 39.5 | 39.2 | 38.9 | 39.2 | 40.5 | 30.5 | 30.7 | 30.1 | 22.7 | 22.5 | 24.5 | 23.4 | 24.8 | 18.1 | 19.3 | 16.8 |
| 5. Char IL-2A | 79.6 | 79.6 | 78.9 | 79.6 | | 43.0 | 40.1 | 40.5 | 40.8 | 41.4 | 27.6 | 30.5 | 26.8 | 25.7 | 25.2 | 22.3 | 22.1 | 28.2 | 20.1 | 17.0 | 15.3 |
| 6. Atlantic IL-2B | 67.9 | 63.4 | 65.4 | 65.0 | 63.9 | | **76.1** | **74.8** | **76.8** | **79.0** | 23.9 | 26.0 | 25.5 | 19.0 | 20.8 | 28.1 | 23.2 | 24.2 | 18.9 | 19.1 | 20.0 |
| 7.Trout IL-2B | 66.4 | 63.4 | 65.7 | 65.7 | 59.2 | 81.8 | | **96.4** | **98.5** | **74.8** | 20.7 | 24.8 | 27.0 | 24.2 | 20.3 | 25.9 | 20.6 | 22.3 | 17.7 | 19.5 | 20.2 |
| 8. Coho IL-2B | 66.4 | 62.0 | 64.5 | 65.0 | 59.9 | 81.2 | 97.8 | | **96.4** | **74.1** | 20.7 | 25.5 | 26.8 | 22.8 | 19.6 | 25.0 | 20.0 | 22.1 | 17.6 | 19.4 | 20.1 |
| 9. Chinook IL-2B | 66.4 | 63.4 | 65.7 | 65.7 | 59.9 | 82.5 | 99.3 | 97.1 | | **75.5** | 20.7 | 24.8 | 27.6 | 24.2 | 20.3 | 25.2 | 21.3 | 22.3 | 17.7 | 19.5 | 20.2 |
| 10. Char IL-2B | 67.1 | 63.4 | 65.2 | 65.7 | 63.3 | 84.1 | 81.2 | 80.4 | 81.2 | | 20.7 | 25.3 | 25.5 | 24.8 | 20.7 | 25.9 | 21.2 | 25.8 | 18.4 | 16.3 | 18.8 |
| 11. Fugu IL-2 | 47.7 | 47.0 | 45.6 | 45.0 | 47.0 | 44.3 | 40.9 | 40.3 | 40.3 | 34.9 | | **43.7** | **35.1** | 21.7 | 21.7 | 22.1 | 25.2 | 20.1 | 19.7 | 19.9 | 21.3 |
| 12. Tetraodon IL-2 | 52.1 | 51.4 | 48.6 | 52.8 | 49.0 | 47.9 | 47.9 | 45.8 | 47.9 | 44.4 | 64.4 | | **30.8** | 20.7 | 23.5 | 24.3 | 25.8 | 25.3 | 20.4 | 17.2 | 21.8 |
| 13. Stickleback IL-2 | 51.4 | 49.3 | 50.4 | 48.6 | 45.6 | 46.3 | 48.2 | 47.8 | 48.2 | 44.2 | 49.7 | 47.2 | | 22.8 | 17.1 | 24.8 | 25.5 | 23.1 | 19.9 | 22.0 | 23.0 |
| 14. Fugu IL-2L | 43.6 | 43.7 | 43.0 | 40.0 | 42.2 | 36.0 | 37.2 | 37.7 | 37.2 | 39.1 | 34.2 | 38.7 | 38.5 | | **38.7** | **29.7** | 20.1 | 21.0 | 20.1 | 18.6 | 21.5 |
| 15. Tetraodon IL-2L | 42.9 | 44.4 | 45.9 | 42.1 | 46.3 | 42.6 | 40.9 | 41.3 | 40.9 | 38.4 | 36.2 | 43.7 | 34.1 | 60.0 | | **27.3** | 21.4 | 20.8 | 18.7 | 17.0 | 18.3 |
| 16. Stickleback IL-2L | 44.3 | 41.5 | 45.9 | 45.0 | 38.1 | 45.6 | 43.8 | 44.2 | 43.8 | 41.3 | 38.3 | 42.3 | 40.7 | 44.3 | 41.5 | | 17.9 | 19.4 | 15.3 | 14.7 | 14.7 |
| 17. Carp IL-2A | 47.0 | 45.6 | 44.3 | 45.0 | 44.3 | 45.0 | 44.3 | 44.3 | 45.0 | 41.6 | 41.6 | 45.0 | 48.3 | 36.9 | 43.0 | 37.6 | | **51.0** | 19.5 | 17.8 | 19.0 |
| 18. Carp IL-2B | 50.0 | 48.6 | 47.9 | 49.3 | 45.6 | 40.8 | 43.7 | 43.7 | 44.4 | 44.4 | 38.3 | 47.2 | 45.1 | 34.5 | 39.4 | 38.7 | 67.8 | | 17.5 | 17.9 | 18.1 |
| 19. Human IL-2 | 37.9 | 34.0 | 31.4 | 33.3 | 38.6 | 37.3 | 37.9 | 37.3 | 37.9 | 35.3 | 36.6 | 38.6 | 34.6 | 34.0 | 38.6 | 32.7 | 37.3 | 36.6 | | **65.2** | **65.8** |
| 20. Cow IL-2 | 38.7 | 37.4 | 34.8 | 38.7 | 40.0 | 40.0 | 40.6 | 40.0 | 40.6 | 38.1 | 38.7 | 35.5 | 34.2 | 36.1 | 32.3 | 31.0 | 39.4 | 38.1 | 77.4 | | **51.3** |
| 21. Rat IL-2 | 36.8 | 36.8 | 38.7 | 32.9 | 36.8 | 38.7 | 41.3 | 40.6 | 41.3 | 38.7 | 40.6 | 36.8 | 38.7 | 39.4 | 32.9 | 34.2 | 37.4 | 36.8 | 78.1 | 68.4 | |
